# Viral maintenance and excretion dynamics of coronaviruses within an Egyptian rousette fruit bat maternal colony: considerations for spillover

**DOI:** 10.1038/s41598-023-42938-w

**Published:** 2023-09-22

**Authors:** Marike Geldenhuys, Noam Ross, Muriel Dietrich, John L. de Vries, Marinda Mortlock, Jonathan H. Epstein, Jacqueline Weyer, Janusz T. Pawęska, Wanda Markotter

**Affiliations:** 1 Centre for Viral Zoonoses, Department of Medical Virology, University of Pretoria, Pretoria, Gauteng South Africa; 2 EcoHealth Alliance, New York, USA; 3 UMR Processus Infectieux en Milieu Insulaire Tropical, Sainte-Clotilde, Reunion Island France; 4 Centre for Emerging Zoonotic and Parasitic Diseases, National Institute for Communicable Diseases of the National Health Laboratory Services, Johannesburg, Gauteng South Africa; 5 Department of Microbiology and Infectious Diseases, School of Pathology, University of Witwatersrand, Johannesburg, Gauteng South Africa

**Keywords:** Virology, Viral epidemiology, Viral reservoirs

## Abstract

Novel coronavirus species of public health and veterinary importance have emerged in the first two decades of the twenty-first century, with bats identified as natural hosts for progenitors of many coronaviruses. Targeted wildlife surveillance is needed to identify the factors involved in viral perpetuation within natural host populations, and drivers of interspecies transmission. We monitored a natural colony of Egyptian rousette bats at monthly intervals across two years to identify circulating coronaviruses, and to investigate shedding dynamics and viral maintenance within the colony. Three distinct lineages were detected, with different seasonal temporal excretion dynamics. For two lineages, the highest periods of coronavirus shedding were at the start of the year, when large numbers of bats were found in the colony. Highest peaks for a third lineage were observed towards the middle of the year. Among individual bat-level factors (age, sex, reproductive status, and forearm mass index), only reproductive status showed significant effects on excretion probability, with reproductive adults having lower rates of detection, though factors were highly interdependent. Analysis of recaptured bats suggests that viral clearance may occur within one month. These findings may be implemented in the development of risk reduction strategies for potential zoonotic coronavirus transmission.

## Introduction

Human population growth is accompanied by urban expansion, agricultural encroachment into natural landscapes, and increased pressure on husbandry practices^1^. These conditions result in more frequent contact between humans and animals (both domestic and wildlife species), increasing the rate at which zoonotic viruses spillover to new host populations^2,3^. The COVID-19 pandemic exemplifies emergence of new disease with global and lasting ramifications. This pandemic was preceded in recent history by the emergence of two other human coronaviruses, Severe Acute Respiratory Syndrome (SARS-CoV) and Middle East Respiratory Syndrome (MERS-CoV)^4^. Research suggests that both emergent and endemic human coronaviruses may have originated in animal reservoirs (domestic and wildlife)^5–9^. Coronaviruses are able to adapt to new host species as a result of recombination, mutational errors during replication, and increased opportunities for transmission to new hosts^10–12^. The latter are largely driven by human activities, such as disruption of natural ecosystems with livestock grazing, agriculture, and wildlife hunting and trade^3^.

Large genetic diversities of coronaviruses have been reported from various families of bats, rodents and birds^4,7,8,11^, with expanding species records being described for the *Alpha-* and *Betacoronavirus* genera^13,14^. A number of coronaviruses identified in bats were determined to be genetically similar to known human viruses (such as 229E, NL63, SARS-CoV and MERS-CoV), particularly among African bat species^8,15,16^. However, much of the identified viral diversity from African bat species remain unclassified^14^. Understanding of the disease ecology of bat coronaviruses that can be used to develop prevention and mitigation strategies to address the risk of spillover has been very limited globally^14,17–25^. Population size, density, roost type, and age composition (maturity) have been implicated as important factors involved in coronavirus prevalence and maintenance within bat populations^10,21,25^, though the impact of these factors on coronavirus maintenance are not in agreement by all studies. For example, though there has been a general consensus from research suggesting higher rates of coronavirus excretion from young bats, the involvement of reproductive activities (such as lactation or pregnancy) as a contributor to viral amplification or persistence within the colony is less certain^17,20^. Whether coronaviruses elicit significant effects to overall body condition has not universally been found to be true, and may depend on the coronavirus species under investigation^10,20^. Additionally, coronavirus maintenance in wild bats has mostly been described without differentiating among multiple but distinct coronaviruses that may be concurrently circulating within a population. Increased contact rates among dense host populations may also potentially result in a larger number of infected individuals, and may cause more viral shedding from a colony, increasing spillover risks. Understanding which factors are involved in viral maintenance within bat hosts and which drivers lead to viral amplification within a population are often based on assumptions concerning available literature derived from different viral families, host species, or ecologies. Context and virus-specific knowledge is essential in developing approaches to potentially disrupt transmission events.

*Rousetttus aegyptiacus* (Egyptian rousette fruit bat) is an ecologically important fruit bat species that provides essential ecosystem services (pollination and seed dispersal) with significant economic benefits^26,27^. Their range spans throughout sub-Saharan and Northern Africa, as well as the Middle East, South East Asia and the Western Palaearctic region^28^. Uniquely among fruit bats in Africa, these bats roost in caves and form large colonies (of up to thousands of individuals) and often co-roost with insectivorous bat species. *R. aegyptiacus* have also been identified as hosts of several viruses, including Marburg virus, paramyxoviruses (Sosuga virus and henipa-related viruses), Lagos bat lyssavirus, adeno-, rota-, and influenza A viruses^29–34^. Bats from the *Rousettus* genus have been reported to host two species from the *Nobecovirus* subgenus (*Betacoronavirus*), with sequences of *Rousettus bat coronavirus HKU9* (first reported from Asia) also described from *R. aegyptiacus.* HKU9 and genetically similar nobecoviruses have been highlighted as a coronavirus group to monitor for emergence due to possible spillover adaptation (from reported recombination and mutation), as they occur within multiple, widely distributed fruit bat species in the Old-World^9,14^. Using a spillover risk ranking tool, HKU9 and several similar African nobecoviruses have also been ranked among the top 40 viruses posing the greatest spillover risk^35^. Though these viruses are also present in African fruit bat species, their excretion dynamics within *R. aegyptiacus* populations have not been well described.

Here, we focused on investigating the excretion dynamics of coronaviruses circulating in a population of *R. aegyptiacus* from a two-year longitudinal study of a maternity colony in South Africa. The colony is located in a cave that is also inhabited by several other bat species (e.g., *Rhinolophus* spp., *Miniopterus* spp., and *Myotis* spp.), and wildlife (Cape porcupines and African rock pythons); with large-spotted genets and vervet monkeys frequently caught on camera traps outside the cave entrance^33^. Moreover, livestock (cattle and goats) from a nearby rural settlement freely roam the area, and have been encountered in the cave. The cave was previously used for traditional and religious practices by the surrounding community, though people still enter the cave. We identified the circulation of distinct coronavirus lineages and characterize the temporal, population, and individual factors associated with infection.

## Materials and methods

### Approvals, ethics, and biosafety

This study was conducted as part of a broader biosurveillance study targeting several zoonotic pathogens in bats in South Africa. Permission to conduct this research was approved by the Department of Agriculture, Land Reform and Rural Development, section  20 of the Animal Disease Act (Act No. 35 of 1984) under reference number 12/11/1/1/8. For sampling bats, provincial permits were obtained (Department of Economic Development, Environment and Tourism of the Limpopo Provincial Government: CPM/11064/2017 & CPM/19774/2018) as well as ethical approval from the following committees and performed according to approved protocols: the University of Pretoria’s Animal Research Ethics Committee, Faculty of Veterinary Science, Onderstepoort, Pretoria (EC054–14, AEC H009-18) and the University of Pretoria Research Ethics Committee, Faculty of Health Sciences, Gezina, Pretoria, South Africa and (REC 639/2018 and EC054-14). All methods were performed in accordance with the relevant guidelines and regulations as stipulated by the institutional ethical approval boards (conforming to ARRIVE guidelines).

Surveillance for coronaviruses was conducted at the *R. aegyptiacus* maternity colony located at the Matlapitsi cave (− 24°6′53.0532′′, 30°7′17.0472′′) bordering Lekgalameetse Nature Reserve in Limpopo Province, South Africa^33,36^. All sampling activities were performed with personal protective equipment (PPE), including Powered Air Purifying Respirators (PAPRs), coveralls, gum boots, double layer nitrile gloves and leather gloves. All equipment, PPE and sample containers were decontaminated with a 10% liquid bleach solution (5500 ppm hypochlorite solution).

### Sample collection

Surveillance was performed monthly between June 2017 and May 2019. Bats were caught at the cave entrance upon emergence using Austbat three-bank harp traps (Faunatech), with traps kept open for five hours each sampling night. All animals captured during this period were sexed and recorded on data sheets. Only a subset of a maximum of 80 bats were sampled per month, whereby equal proportions of population demographics by sex and age were randomly taken for sample collection (also representing a sufficient sample size that could reliably be targeted with the available manpower over the duration of the sampling nights). During months when certain population cohorts were absent from the colony (e.g., adult males), numbers of other cohort groups sampled were increased. Samples (blood, urine, oral and rectal swabs) were collected from *R. aegyptiacus* as described in Mortlock et al.^33^. Sample types used in this study include rectal swabs from individual bats as well as pooled fecal samples collected inside the cave. Small sterile swabs (VWR Critical Swab) lubricated with 1× sterile PBS (Lonza) were inserted into the rectum and rotated twice to collect rectal swab samples and immediately frozen in a dry shipper (MVE Vapor Shippers). During the day, fresh fecal samples (non-desiccated and deposited within the last 24 h) were collected inside the cave from rock surfaces (using sterile swabs to scoop up fecal only). Three fecal deposits (often spatially clustered) were pooled as one sample and immediately frozen in the dry shipper. Soft (pulpy) fecal samples derived from *Rousettus* were collected with certainty, as insectivorous species in the cave bats produce hard pellets (and do not intermingle in roosting spots with *Rousettus).* No studies have been performed on the age structure of the bats in the cave (e.g., younger bats located near the entrance), though fecal deposits were collected underneath roosting bats throughout the cave. Pooled fecal samples were collected in all 24 months; rectal swabs were only collected in 16 months (due to various logistical challenges faced, such as extremely poor weather conditions).

### Morphological measurements and mark-recapture methods

A set of demographic and morphological measurements were taken from each individual bat including sex, reproductive status, forearm measurements and weight of the bat. Female bats were recorded as ‘reproductively active’ via the observation of pregnancy, lactation, or tapered/sclerotized nipples; non-reproductive females (nulliparous) possessed non-sclerotized nipples and determined to not be pregnant via palpation of the abdomen. Males were recorded as either being scrotal (reproductive) or non-scrotal (non-reproductive). Age was estimated using forearm lengths, with a cut-off of 89 mm and above to designate ‘adult’ bats^37,38^. ‘Subadults’ were categorized as free-flying independent young bats with a forearm length of below 89 mm that have yet to reach sexual maturity via aforementioned criteria^37,38^. Bats deemed reproductively active (due to evidence of secondary sexual characteristics and reproduction) were categorized as ‘adults’ regardless of forearm length. Pups attached to female bats were not sampled due to concerns of separation. Each sampled bat also received a unique tattoo for mark-recapture^39^. Bats recaptured more than once during the same sampling trip per month were released and not sampled again.

### Viral RNA detection

RNA extraction and inactivation of samples were performed in a BSL3 laboratory, after which processing for nucleic acid testing was performed under BSL2 conditions. Fecal pools and rectal swabs were resuspended in 800 µl or 400 µl (respectively) 1× PBS (Lonza) and gently mixed. A total of 200 µl suspension was inactivated with an equal volume of 2× DNA/RNA shield solution (Zymo Research). Total RNA was extracted from inactivated material using the Quick-RNA™ Miniprep Plus Kit (Zymo Research). Complementary DNA was prepared as 20 μl randomly primed reactions using 100 ng random primers (Integrated DNA Technologies) and Superscript IV (Invitrogen) according to manufacturer’s recommendations. An in-house *Alpha*- and *Betacoronavirus* specific hemi-nested RT-PCR targeting the *RNA dependent RNA polymerase* (RdRp) gene was used as a surveillance assay as described in Geldenhuys et al.^40^, yielding a nested amplicon of 268 bp. After analyses of nested products on a 1.5% agarose gel (Lonza), all products of appropriate size were excised and purified with the Zymoclean™ Gel DNA Recovery Kit (Zymo Research). Purified amplicons were prepared for sequencing with the BigDye Terminator v3.1 Cycle Sequencing Kit (Thermo Fisher Scientific) according to manufacturer’s recommendations for 10 μl reactions and purified with the ethanol/EDTA/sodium acetate precipitation method. Sequencing was performed on the ABI 3500xl at the DNA Sequencing facility of the University of Pretoria. Sequences were submitted to Genbank with accession numbers: MZ547450-MZ547649.

### Phylogenetic analysis

Sequence manipulations and alignments were performed with CLUSTALW in the BioEdit sequence alignment editor (v7.2.5)^41^. Sequences of relevant coronavirus species and similar reference genomes identified from BLAST analyses of sequences from this study were collected from GenBank (NCBI). Estimations of pairwise similarities were performed with p-distance analyses in MEGA v7^42^. Phylogenetic analyses were performed with Bayesian phylogenetics using BEAST v.1.10.4^43^. CIPRES Science Gateway was used to run jModelTest2 and BEAST^44^. Maximum clade credibility trees were constructed with the general time reversible (GTR) model with gamma distribution and invariant sites. Bayesian MCMC chains were set to 25,000,000 states, sampling every 2500 steps, and convergence confirmed via an effective sample size (ESS) of > 200. Final trees were calculated in Tree Annotator with a 10% burn-in^45^. Trees were viewed and edited in Figtree v1.4.2.

### Statistical analysis

A multinomial generalized additive mixed model (GAMM) was used to jointly model the dynamics of all three viruses in both rectal swabs and pooled fecal samples^46^. We included overall and annual cyclic time-varying splines to account for temporal variation. These and model intercepts were stratified by sample type. For rectal swabs where samples could be attributed to individual bats, we included hierarchical splines to test whether dynamics varied by the sex or age of the bats^47^. We included a penalized random effect term to test if reproductive condition category (scrotal, lactating, or pregnant) affected probability of positivity in rectal swabs, as well as a nonlinear term for the effect of forearm mass index (FMI, forearm m/body mass kg) as per Meng et al.^48^ As sex, age, and reproductive condition were correlated with FMI, it was normalized within these categories to reduce collinearity. All terms were included in a single model as the analytical goal was to test and compare the effects of multiple factors^49,50^.

The GAMM initially fit with the restricted maximum likelihood estimation method using the mgcv package^51^ in R 4.2.1^52^, then used these estimates to initialize Metropolis–Hastings MCMC sampling of the model posterior. Spline wiggliness was penalized to reduce to linear effects in the absence of support for nonlinear effects. Convergence and efficiency of MCMC chains were checked using the R-hat and Effective Sample Size statistics^53^. Significance of nonlinear model terms was determined using Nychka criteria^54^, and reported model estimates and confidence intervals as means and 95% high-density intervals of posterior predictions. Both raw and model-estimated values of prevalence in pooled fecal samples were converted to individual-sample level using the Burrows estimator^55^, which corrects for upward bias in pooled sampling.

## Results

### Coronavirus presence and diversity detected

A total of 720 fecal pools and 710 rectal swabs were tested for coronavirus nucleic acid with a conventional RT-PCR assay^40^. This resulted in a combined 200 positive samples, 94 from rectal samples (raw detection rate 13.2%) and 106 from fecal pools (raw detection rate 14.7%, corrected to 5.2% when accounting for pooling). Model-estimated average (intercept) detection rate was 9.6% (CI 7.3–11.9%) for rectal swabs, and 3.3% (CI 2.5–4.1%) for fecal samples after correcting for pooling (Table S1). The detection rate among rectal swabs was not statistically different from that estimated for the overall uncorrected pooled fecal samples (Table S1). Datasets for samples containing coronavirus RNA are listed in supplementary dataset S1 and S2.

**Figure 1:**
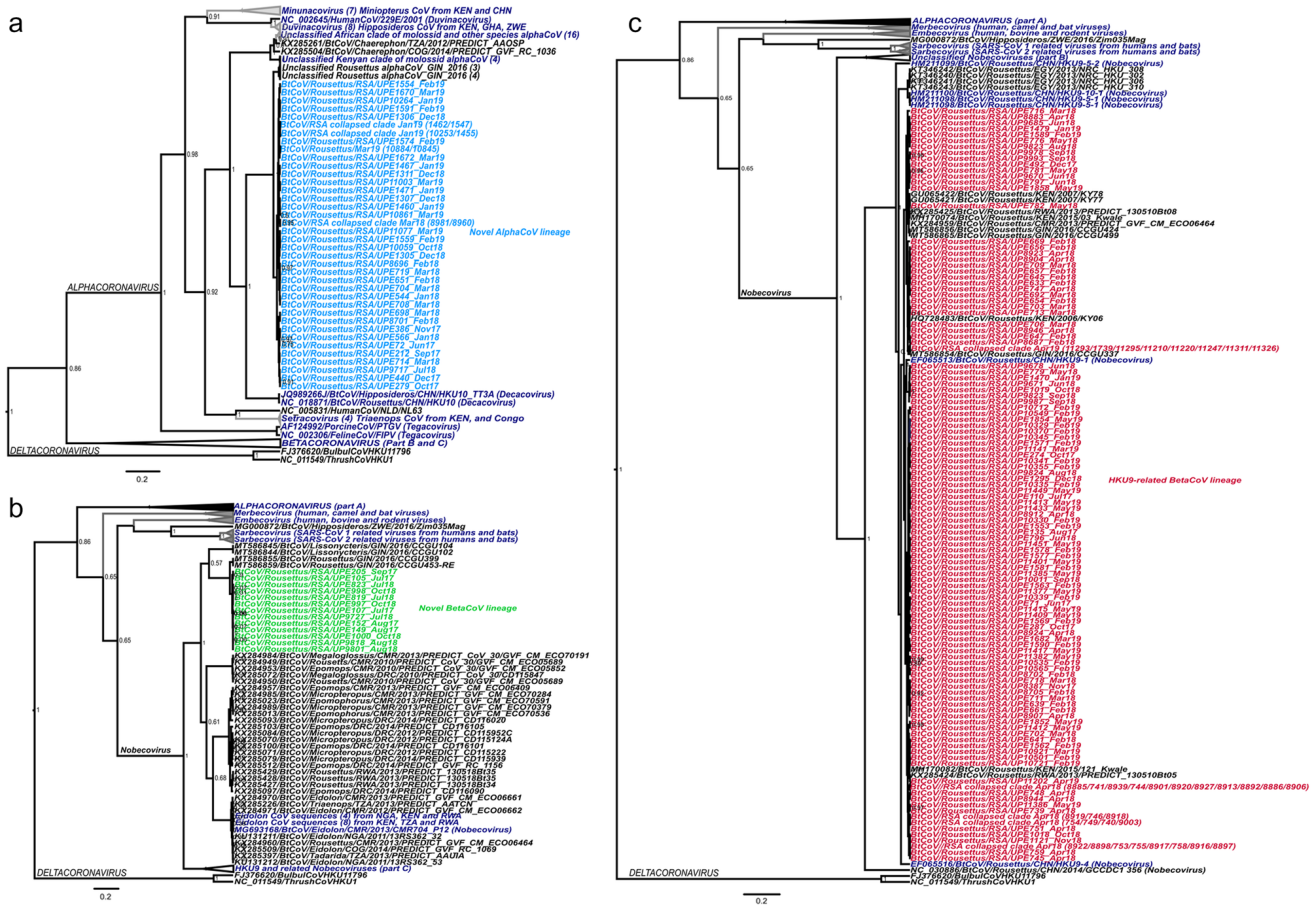
Bayesian phylogeny of the coronaviruses identified in the study. The phylogenetic tree was split into three parts for better visualization (with collapsed clades indicated). The *Alphacoronavirus* genus is shown in part A, the RouNobeCoV betacoronavirus identified in this study with related African sequences in part B, and the nobecoviruses related to the HKU9 species are shown in part C. Sequences in navy blue indicate reference species, specific subgenera or collapsed clades (to improve visualization). Sequences in light blue refer to the RouAlphaCoV, green to RouNobeCoV and red for the dominant HKU9-lineage sequences. Only posterior probabilities of greater than 0.5 are indicated. Three-letter country codes indicate sequence origins.

Three coronavirus lineages were identified to be circulating within the *Rousettus* colony following sequencing of the PCR positive samples (Fig. 1). Sequences (n = 42) of an alphacoronavirus (RouAlphaCoV) lineage shared 96–100% nucleotide identity in the short amplified conserved region of the RdRp gene (240 bp). These sequences were closely related to uncharacterized alphacoronaviruses recently reported in the same bat species from Guinea (93.8–96.2% nucleotide identity)^56^. They grouped with members of the *Decacovirus* subgenus (*Alphacoronavirus*) identified in Asian bat species (*Bat coronavirus HKU10* from *Rousettus leschenaulti* (NC_018871) and *Hipposideros pomona* (JQ989266)), sharing 78–79% nucleotide identities (Fig. 1). These *Rousettus* sequences likely belong to an undescribed alphacoronavirus species and may be a member of the *Decacovirus* subgenus.

Two *Nobecovirus* subgenus betacoronavirus lineages were detected. Sequences from the most abundant lineage (n = 145) were 94.9–100% identical at a nucleotide level (within the short-amplified region of the RdRp gene) and detected throughout the two years of surveillance. These sequences shared close similarities to members of the *Rousettus bat coronavirus HKU9* species (referred to hereafter as the HKU9-lineage, Fig. 1). Sequences similar to HKU9 have frequently been reported from several African fruit bats^56–61^, which are phylogenetically interspersed with the sequences from this study (Fig. 1). The detected HKU9-lineage sequences shared high (92–100%) nucleotide similarities to sequences (HQ728483, MT586856, GU065422, KT346240) from *Rousettus* hosts in other parts of Africa (Kenya, Guinea and Egypt); and between 94–98% identity to HKU9 strains from Asia^56–59^. The second betacoronavirus (RouNobeCoV) lineage identified only shared 74.2–76.7% identity to the detected HKU9-lineage sequences and 79.2–80.1% identity to an unclassified clade of coronaviruses originating from multiple African fruit bat host genera (*Megaloglossus*, *Epomops, Eidolon,* and *Rousettus*) (Fig. 1) from various African countries^56,60^. The closest sequences originate from *Rousettus aegyptiacus* in Guinea (MT586855) with similarities of 95.7–96.2%.

### Factors influencing coronavirus excretion dynamics

**Figure 2:**
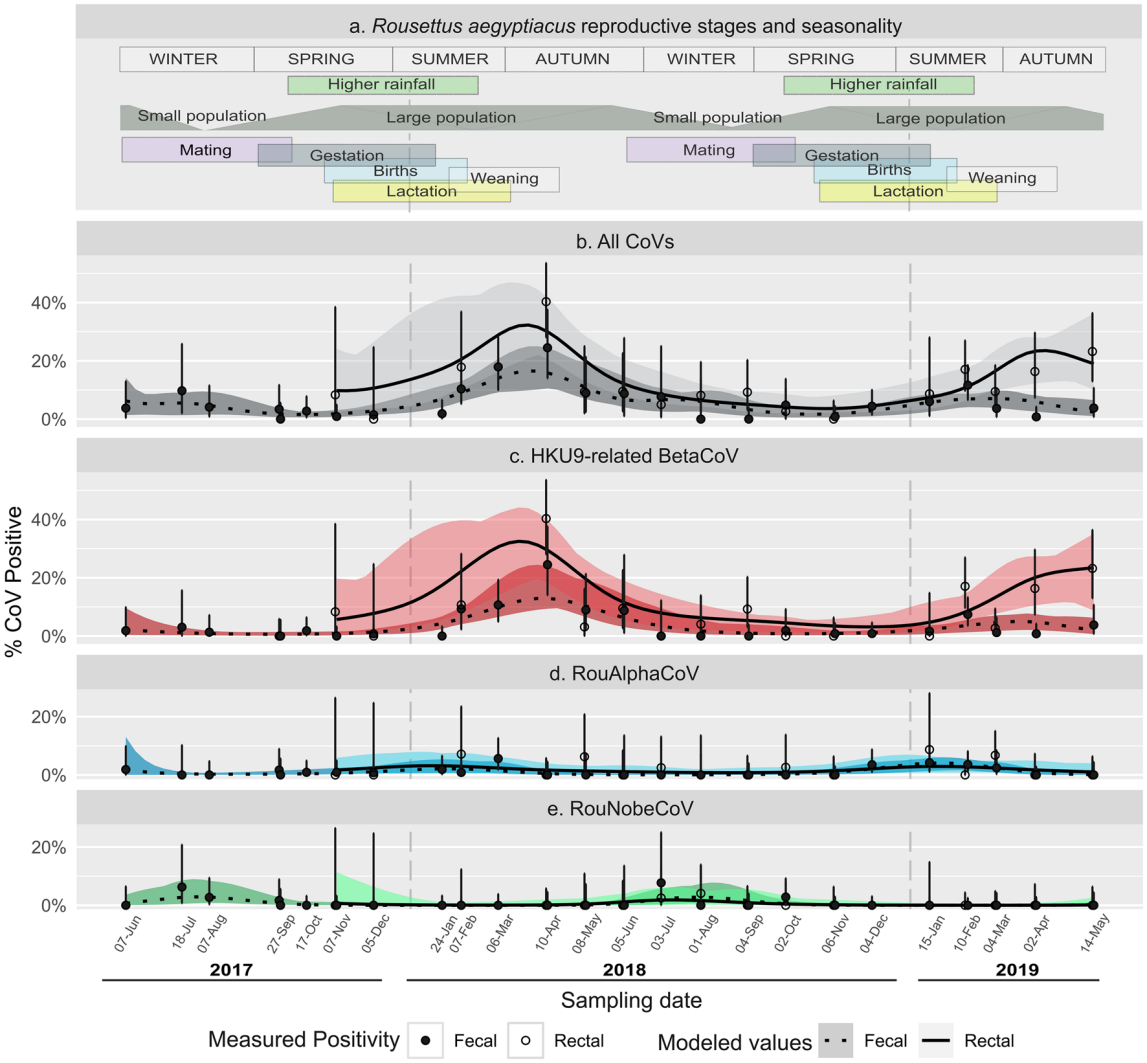
Reproductive cycle and coronavirus excretion dynamics in *Rousettus aegyptiacus.* (**a**) schematic representation of the timing of reproductive stages and general rainfall; (**b**) Total GAM-estimated coronavirus prevalence and environmental detection rate over time (individual rectal swabs—solid lines; pooling-corrected fecal samples—dotted lines), and raw positivity at specific sampling dates (rectal swabs—empty circles; pooling-corrected fecal samples—filled circles). Shaded areas and error bars areas depict 95% posterior intervals for model predictions and binomial intervals monthly samples, respectively. (**c**) Coronavirus detection dynamics for the HKU9-related lineage only, (**d**) detection of the RouAlphaCoV lineage and (**e**) detection of the RouNobeCoV lineage.

Coronavirus excretion varied considerably over time and between lineages, ranging from 0% of positive samples in monthly sampling events to over 40% of rectal swabs positive in April 2018, with smaller peaks observed in the first half of 2019 (Fig. 2). The HKU9-related lineage dominated positive results, accounting for 83% and 63% of the total detected coronaviruses from swabs and pooled fecal samples, respectively. To estimate temporal (long-term and seasonal) individual-level (age, sex, reproductive state, and body condition) effects on excretion, a multinomial, nonlinear generalized additive mixed model (GAMM) was fit to the observed positivity of each viral lineage in rectal swabs and fecal samples (the latter not linked to specific individual bat demographics). The estimated average prevalence of the dominant HKU9-related lineage was 7.7% (CI 5.7–9.7%) for individual rectal swabs with detection rate of 2.6% (CI 1.9–3.3%) for fecal samples, corrected for pooling. The estimated average prevalence of the RouAlphaCoV lineage was 1.7% (CI 1.0–2.6%) for individual rectal swabs and detection rate of 0.6% (CI 0.3–0.9%) for corrected fecal samples. The RouNobeCoV lineage was detected rarely (n = 13) and mostly from fecal pools (10 of 13). The estimated average prevalence of this lineage was 0.2% (CI 0.0–0.4%) for individual rectal swabs, and the detection rate for fecal samples was between 0 and 0.1%.

In Fig. 2 the observed and GAMM-predicted viral prevalence over time is overlaid with key events in the reproductive cycle of *R. aegyptiacus* in this maternity colony, as assembled from the literature^38^ and field observations. There are bats present at the maternal roost throughout the year, though the population fluctuates depending on rainfall and food availability. Mating occurs during winter and the start of spring (June to mid-September), where the roost is ‘re-colonized’^62^. Gestation lasts approximately four months with pups first seen around the end of October each year while peak parturition is seen in November–December^38,63^. The population is at its largest in summer/autumn at the start of the year (February-April), during which young bats are also becoming independent. At the start of winter, many bats in the population leave the maternity roost, with only a small population remaining in the roost over winter (June–August). These changes in colony populations are reflected in the demographic makeup of bats (Fig. S2).

**Figure 3:**
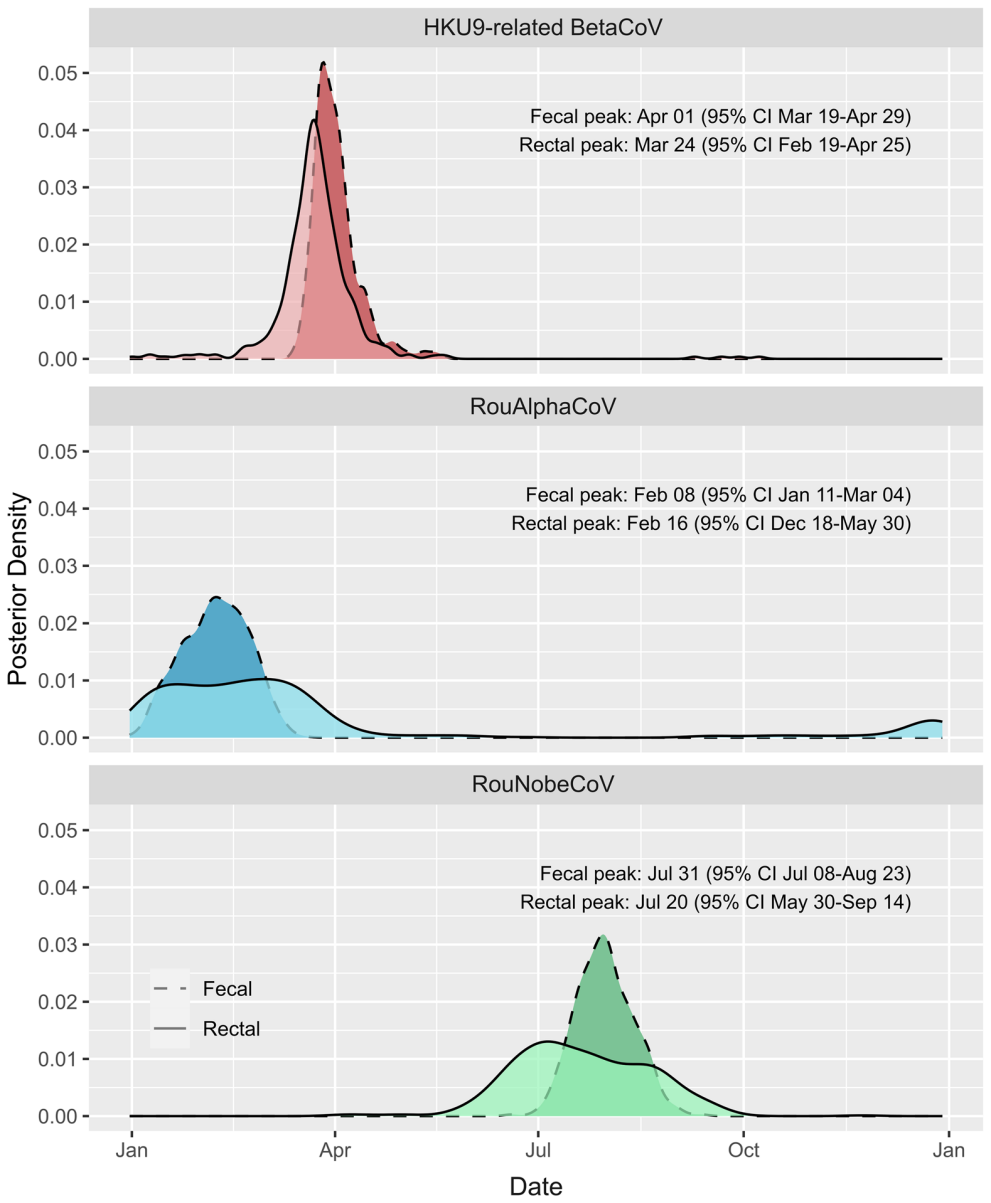
Estimates of peak seasonal dates of excretion for each coronavirus lineage. Plots show the GAMM posterior density for the date of highest rates of detection for fecal (dotted) and rectal (solid) samples. Rectal and fecal peak date distributions overlap within each lineage, while across lineages the peak date differs in > 95% of posterior samples for fecal samples of all lineages and overlaps only across rectal samples from the HKU9-related and RouAlphaCoV lineages.

All three lineages have significant seasonal effects (*p* < 0.05, Table S1), with distinct timings of peak prevalence at different times of the year (Figs. 3, S3). The HKU9-related lineage showed greatest positivity from mid-March to late-April in fecal samples, and mid-February to late-April in rectal samples. The RouAlphaCoV lineage prevalence peaked between mid-January and early-March in fecal samples, and mid-December to late-May in rectal samples. The RouNobeCoV peaked later in the year, with highest prevalence in fecal samples from early-July to late August and late-May to mid-September in rectal samples. Peak dates differed across all lineages (*p* < 0.05) except for rectal samples of the HKU9-related and RouAlphaCoV lineages. The wider range of estimates in rectal samples is attributable to the smaller number of positive detections. In addition, the HKU9-related lineage exhibited an overall interannual decline in positivity in fecal samples over the sampling period (*p* < 0.01; Table S1, Fig. S1).

**Figure 4:**
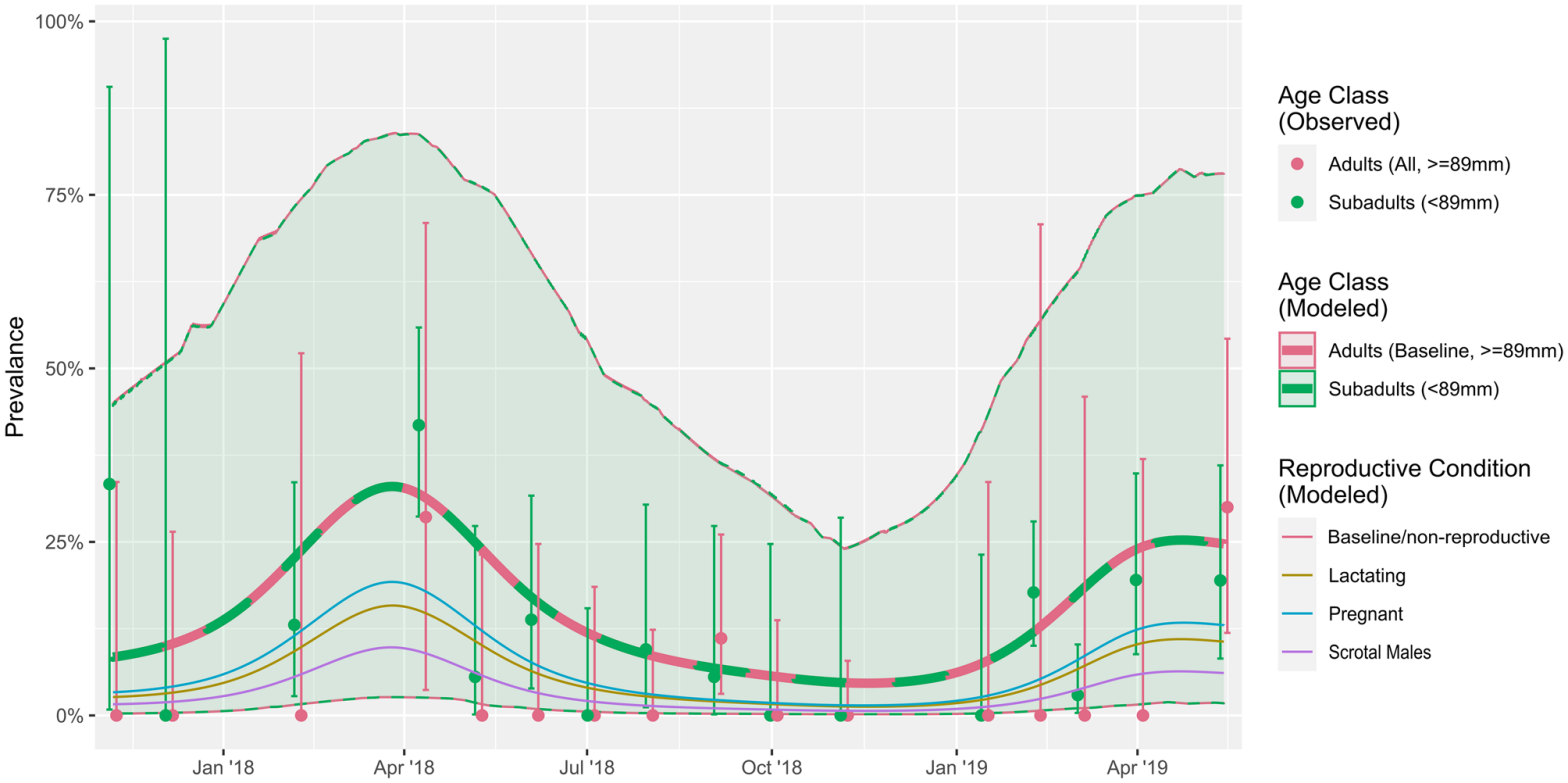
Effect of demographic factors on prevalence for the HKU9-related BetaCoV lineage. Points and whiskers represent observed and 95% intervals for observed prevalence on sampling dates for all adults (red) and subadults (green). The thick line and shaded area show modelled estimates of prevalence for adults and subadults over time. The overlap indicates no significant effect. Thin lines show modelled estimates of prevalence for adults, as modified by the effects of different reproductive conditions: lactating (brown), pregnant (blue) or scrotal (purple). All estimates are conditional on a mean within-group FMI.

Rectal swabs could be linked to individual bat traits allowing assessment of the effects of sex, age, reproductive status, and body condition on prevalence (for RouNobeCoV, there were too few positive samples to test these effects). Raw data prevalence estimates per demographic group showed that coronavirus RNA was more frequently detected in subadults than adults (10.85% and 2.39%, respectively) (Table 1). However, age, sex, and reproductive condition are strongly interdependent (Fig. S3). Only reproductive condition was a significant predictor of viral prevalence for the HKU9-related lineage (*p* < 0.05, Fig. 4, Table S1) in the context of all individual-level variables in the GAMM. Non-reproductive individuals (not reproductively active at the time of sampling) were consistently more likely to be positive than reproductively active individuals (scrotal, pregnant, or lactating).

**Table 1:** Coronavirus detection and proportion positives per demographic group.

Population group	Total present	Number of CoV sequences detected (RouAlphaCoV/ HKU9-related/ RouNobeCoV)	Proportion positive % per demographic subgroup (95% CI)	Overall positive % (95% CI)
Adults (overall)	259	17 (3/12/2)	6.56 (4.11–10.3%)	2.39 (1.40–3.81)
Adult females	136	13 (2/9/2)	9.56 (5.59–15.9)	1.83 (0.98–3.11)
*Non-pregnant*	70	10 (1/8/1)	14.3 (7.74–24.9)	1.41 (0.68–2.57)
*Pregnant*	51	3 (1/1/1)	5.88 (1.83–17.3)	0.42 (0.09–1.23)
*Lactating*	15	0	–	–
Adult males	123	4 (1/3/0)	3.25 (1.21–8.45)	0.56 (0.15–1.44)
*Non-scrotal*	39	2 (0/2/0)	5.13 (1.20–19.3)	0.28 (0.03–1.01)
*Scrotal*	84	2 (1/1/0)	2.38 (0.58–9.27)	0.28 (0.03–1.01)
Subadults (overall)	451	77 (10/66/1)	17.07 (13.7–20.8%)	10.85 (8.65–13.37)
*Females*	227	38 (5/33/0)	16.7 (12.4–22.2%)	5.35 (3.82–7.27)
*Males*	224	39 (5/33/1)	17.4 (13.0–23.0%)	5.49 (3.93–7.43)
Total	710	94 (13/78/3)	13.2 (10.9–15.9)

Forearm mass index (FMI) was used as a measure of body condition^48^, and average FMI for adult bats was 13.6 kg/m^2^ (5.7–20.3) and an average of 11.6 kg/m^2^ (5.8–16.7) for subadults (Table S2). As FMI was strongly determined by other variables, especially reproductive condition (Fig. S3), it was normalized within age, sex and reproductive condition classes for model-fitting. Normalized FMI was not found to have significant effects on prevalence of any virus lineage, though for both the HKU9-related BetaCoV and RouAlphaCoV lineages there was a downward trend (*p* = 0.17, *p* = 0.17).

### Recapture-directed estimates for duration of viral excretion

**Figure 5:**
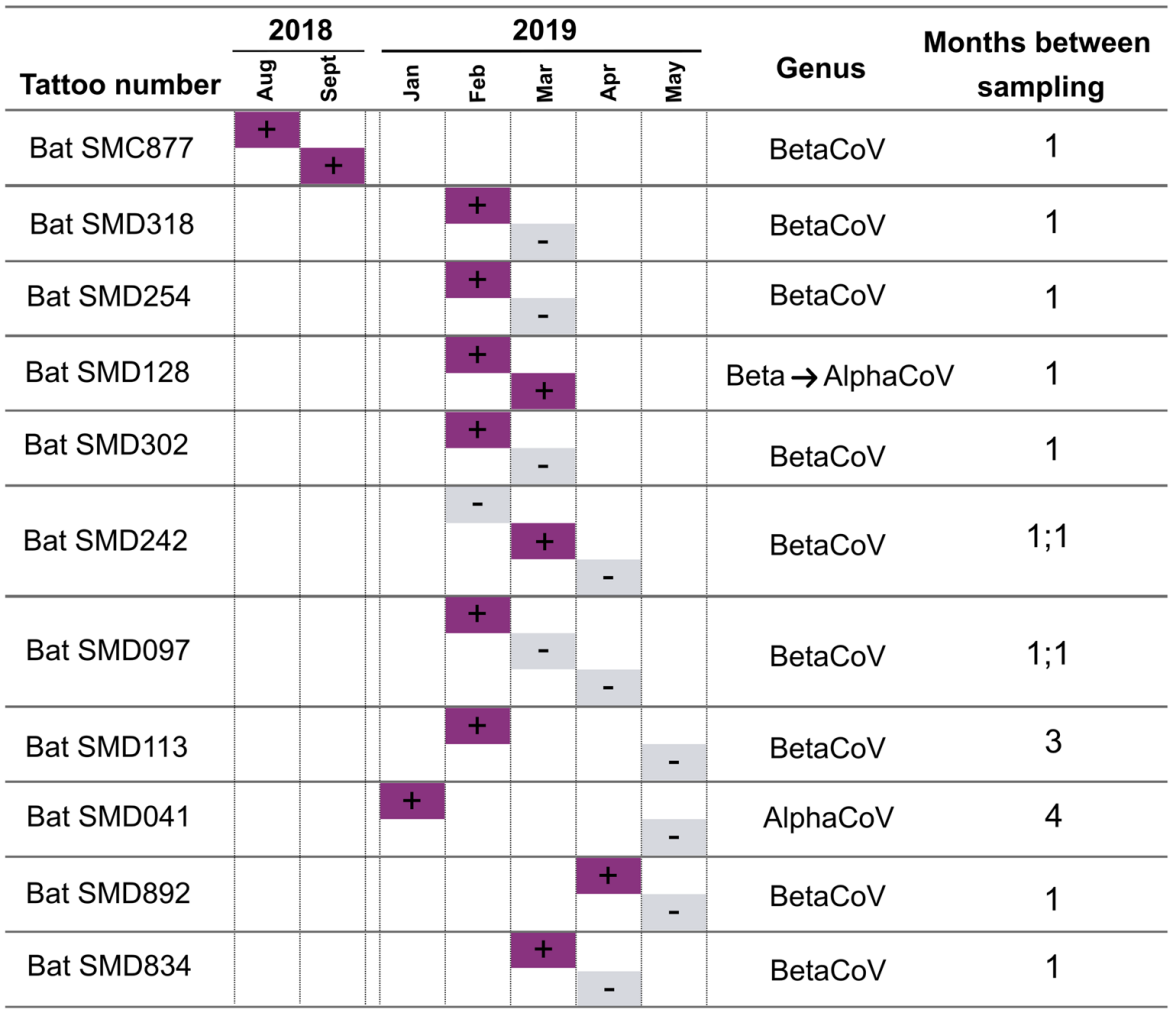
Details of the 11 bats excreting coronavirus RNA at their initial capture or second recapture, with subsequent recapture events. Bat tattoo numbers are provided as identity tags and the months captured indicated. Coronavirus RNA detected: purple/‘plus’ symbol; absence of coronavirus RNA: grey/‘minus’ symbols. The genus of detectable coronaviruses and months between recaptures are also indicated.

Lastly, recapture data was used to compare detectable duration of viral excretion. For estimation of possible viral clearance or persistent infections, bats need to be frequently recaptured and tested for coronavirus RNA, preferably shortly after the initial detection of excretion. A total of 75 bats (10.6%) from the 710 individuals sampled were recaptured. Among the recaptured bats, RNA was detected from 20 individuals. In nine recaptured bats, coronavirus RNA was detected only at their last recapture indicating bats were infected since their first capture. The remaining 11 bats represented individuals from which viral RNA was detected at their first or second capture, though not at subsequent captures (Fig. 5), which may be used to estimate possible viral clearance times. The shortest duration between recaptures was one month, with the longest being four months. Coronavirus RNA was only detected spanning more than one month in two bats—SMC877, excreting the same HKU9-related lineage a month apart, and SMD128, excreting RNA from the HKU9-related lineage, then RouAlphaCoV in the following month. It can be estimated that viral clearance may occur within one month (approximately four weeks), though some bats may take longer for viral clearance or persistently excrete viral RNA.

## Discussion and conclusion

The limited research available to identify and predict ecological or biological factors involved in the natural maintenance of bat coronaviruses was previously highlighted^14^. Knowledge of these factors can aid development of evidence-based strategies for possible reduction of potential risks for zoonotic transmission as alternatives to population culling, which have repeatedly been shown to have serious consequences for local ecology, environment, and public health^21,64,65^. The fruit bat species, *R. aegyptiacus,* is an important host for diverse viral families, including filoviruses, paramyxoviruses and coronaviruses^16^. In southern Africa, the species has one birthing cycle in contrast to central/northern parts of Africa, with two birthing pulses^21^.

Overall, coronavirus RNA was detected from a model-estimated 9.6% of rectal swabs and 3.3% of fecal pools (accounting for pooling correction). These results (and raw prevalence of 13.2% of rectal swabs and 5.2% of corrected fecal pools) are within the range reported by other similar longitudinal studies in Asia (4.2–18.5%)^20,22^, and comparable or higher (5.5–14.2%) than previous cross-sectional studies in *R. aegyptiacus*^56,59,60,66^. The study revealed the circulation of three lineages in the population within the sampling time frame, though no co-infections were identified. Co-circulation of alpha- and betacoronaviruses have previously been recorded for *R. aegyptiacus* and other fruit bats^20,56,66^ as well as other bat species^10^. By examining the excretion of the separate lineages, coronavirus excretion from the *Rousettus* population was determined to exhibit strong but distinct seasonality, with differences in timing between the viruses (Figs. 2, 3, S1). The dominant HKU9-related and RouAlphaCoV lineages both had peak excretion early in the year (Figs. 2, 3) coinciding generally with higher food abundance, and a larger population in the roost due to the presence of young bats becoming independent. The unclassified RouNobeCoV lineage was detected infrequently during our sampling time frame and only at later times of the year, coinciding with low food availability, mating, and early pregnancy within the population. These effects may be driven by seasonal changes in the environment, increased contact rates between many individuals occupying the same roost (for the HKU9-related and RouAlphaCoV lineages at the start of the year) or may be mediated by other factors relating to population demographics and virus-specific factors. Although coronavirus excretion was detected throughout the year, the findings suggest February-April is the time of greatest possible spillover risk due to high excretion. Within other populations of *R. aegyptiacus* in Africa, increased risk periods have been identified between November-February and April-September, and reflects the bi-annual bat birth pulses observed along the equator^21^. These differences would highlight the importance of investigating both varying host ecologies and viral excretion patterns among similar host species to evaluate factors associated with spillover risk.

Fecal samples collected beneath roosting bats (pooled) serve as a simple non-invasive alternative to swabbing bats in the hand for determining coronavirus excretion from a colony, since they are easily collected with minimal disturbance to the colony. They also provide a larger volume of material to enable an additional number of molecular assays to be performed (such as genome characterization) or for viral culture. However, since fecal pools cannot be associated with individuals, inferences of how individual host traits influence excretion or timing among demographic groups are limited and are investigated via individual bat capture and sampling. Moreover, rectal swabs from individuals also enable estimation of viral clearance in recaptured bats.

Higher coronavirus detection (from primary/raw data) was found among subadults than adults (10.9% compared to 2.4%, respectively). However, reproductive condition, rather than age, was the only significant individual-level predictor of prevalence when all effects were considered jointly, with non-reproductive individuals (at the time of sampling) having higher prevalence than reproductively active individuals (scrotal, pregnant, or lactating adults). This is likely because the interaction between age, sex, reproductive and body condition masks individual causal effects—subadults and non-reproductive adults are more alike in prevalence than reproductive adults. Targeted, more intense and longer sampling designed to disentangle these effects may in the future allow us to determine the role of reproductive stresses in driving the dynamics of these coronaviruses.

FMI may be considered a good measure of body mass quality among bats^48^, and in both adult males and females, a higher FMI among reproductively active individuals is generally linked to increased resource allocation for reproduction (such as increased weight of the foetus/milk during gestation). Physiological stresses of reproduction have previously been suggested as risk factors increasing susceptibility of bats to possible infection of diverse viruses^17,67,68^. No significant evidence of FMI and prevalence associations were found among these three viral lineages from *Rousettus* at this time, and further data may enhance our ability to measure these effects.

Very limited longitudinal surveillance studies among specific bat populations have investigated the maintenance of bat coronaviruses. Moreover, there is little consistency in statistical frameworks for these studies, contributing to challenges in comparing results. Factors influencing coronavirus excretion have been reported based on simple significance testing on individual variables (such as Chi-squared, Fishers-exact/2-tailed tests or Analysis of Variance (ANOVA) between two or more groups)^17,18,20^, and multivariate analyses using generalized linear (mixed) models (GLMs and GLMMs)^22,24^ or general additive mixed models (GAMMs)^23^. In this study, a multinomial GAMM^46^ was used to jointly model the dynamics of multiple viral lineages in different sample types while avoiding multiple-testing challenges, and used this to examine the seasonal, interannual, and individual variation in viral excretion. Like other longitudinal studies, differences in coronavirus prevalence between age-classes was found in raw data, but under the scrutiny of multivariate analyses these effects could not be disentangled from other co-varying factors.

It has been suggested in literature that an increase in coronavirus circulation among young bats (assumed across all coronavirus lineages) occurs towards the end of the weaning period, presumably due to a gradual loss of maternally-derived antibody protection^20,21^. Such infection dynamics within maternity colonies has been suggested for various bat hosts and coronavirus species^18,22,23^, as well as other viruses such as Marburg virus and novel paramyxoviruses^62,69^. Serological information on the duration of maternal-antibody protection, general immunity as well as possible cross-protection of different bat coronaviruses is a key research area with very limited data. Alternative circulation strategies, such as those described for viruses like Nipah virus, include non-seasonal cycles in bat colonies, driven by immune dynamics and viral re-introduction/recrudescence rather than seasonal cycles^70^. Under multivariate analyses, the findings from this longitudinal study (over approximately 2 years) determined that individual-level effects, particularly of age, could not be separated from other co-varying factors. Reproductive condition was the only individual-level predictor of viral prevalence (and only for the HKU9-related lineage). It is possible that additional age-prevalence data from multiple seasons will allow for more precise investigation of the causal hypotheses.

Generally, the mode of coronavirus infection in bats remains largely unclear. Specifically, if persistent infections with either chronic or intermittent shedding occur, or whether acute, transient infections lead to viral clearance. Research by Jeong et al.^19^ suggests a combination of both mechanisms may be contributing to population-level maintenance of bat coronaviruses. Intermittent shedding from persistent infections may cause pulses of viral amplification in the colony^19^, which can be monitored and allow continual re-seeding of the virus into the population. Moreover, the duration of bat coronavirus immunity for transient infections with clearance is currently unknown (due to a lack of serological studies). Viral lineages would rely on reinfection events to prevent viral extinction. Due to limited evidence, most research focusing on the disease ecology of bat coronaviruses rely on certain general assumptions, including that the majority of bat coronaviruses likely follow the same general trend of host infection. Using recapture data representing 10% of the bats sampled, a preliminary timeframe for potential viral clearance could be estimated to occur within at least four weeks. This is comparable both in the percentage of recaptures and clearance estimations of two weeks to four months from other studies^10,71^. Though the results may appear to indicate transient infections, persistent infection with intermittent shedding cannot be ruled out based on the small number of recaptured bats in this study (with a reinfection noted between viral genera as well as one bat excreting viral RNA for a longer than 4 weeks). As the results are based on the detection of viral nucleic acids, conclusions on whether identified nucleic acids represent excreted infectious viruses should be made with caution (as non-infectious remnants may also be shed).

This research has shown that factors associated with coronavirus maintenance among bat populations may not be universal among coronavirus species within bat populations. Two years of monthly surveillance has provided an indication of excretion dynamics from the colony, though only continued sampling at this location may determine if the high excretion times are consistent and whether specific lineages may be outcompeted. In addition, full genome characterization of these viral lineages (currently underway and not the focus of this research paper) will also enable investigations of possible recombination between lineages. A limitation of the study includes sufficient sample sizes per demographic cohort (sex, age, reproductive condition) per month, due to the natural variation among the host populations at certain times of year. Improved stratified sampling designs to ensure sufficient sizes per available demographic group can shed more light on the drivers of viral circulation. Use of FMI as a metric of estimating body condition and health has advantages and disadvantages^48,72^, and future studies will incorporate more approaches to estimating bat health status. Serological surveillance of coronaviruses in bats is severely lacking, largely due to limited biologics available for reliable assay development. This should be a priority research area to enable better understanding of coronavirus-associated immune protection or susceptibility. Bat models for experimental host-virus studies are also lacking, and may provide valuable data about infectious period, correlation between viral shedding and RNA detection, and viral transmission among individuals, that can be used to parameterize models of infection (and shedding) dynamics in wild populations.

Longitudinal surveillance studies, such as this study, provide valuable information regarding the circulation of viruses and active assessment of factors (seasons, reproductive stages or additional stressors) that affect viral maintenance. The Matlapitisi colony is an ideal interface to investigate viral excretion dynamics within a maternity roost, as the bats in the colony interact with other bat species^62^, terrestrial wildlife, domestic animals, and people (in the cave and surrounding areas). This study served as the groundwork for coronavirus surveillance at this site, identifying the diversity within this species and leading to a basic understanding of viral excretion patterns during the year. Future research will continue monitoring the viral dynamics at the colony over time, examine co-infection between different viral families, cross-species transmissions to other bats in the cave, assess land-use changes potentially impacting risk of spillover to livestock and people, and expand surveillance to include other animals and human populations living in close association with this population of bats. Studies that assess viral dynamics in free-ranging host populations living in close association with people and domestic animals can provide useful insights into the risk of spillover and may help develop interventions to protect both human and animal health.

### Supplementary Information

Supplementary Information.

## Data Availability

All data generated during this study are included in this published article (and its Supplementary Information files). All model code as well as diagnostic reports are available in the GitHub project code repository (https://github.com/ecohealthalliance/sabrenet-rousettus-dynamics/, with a version on Zenodo https://zenodo.org/record/7709716).
