# Supplementary material for: Viral maintenance and excretion dynamics of coronaviruses within an Egyptian rousette fruit bat maternal colony: considerations for spillover
Source: Sci Rep. 2023 Sep 22;13:15829. doi: 10.1038/s41598-023-42938-w (PMC10517123; doi:10.1038/s41598-023-42938-w)
Supplement: Supplementary file 1 — Supplementary Information. [file 41598_2023_42938_MOESM1_ESM.pdf]

Supplementary Information for

Viral maintenance and excretion dynamics of coronaviruses within an Egyptian rousette fruit bat maternal colony: considerations for spillover

Marike Geldenhuys[1], Noam Ross[2], Muriel Dietrich[3], John L. de Vries[1], Marinda Mortlock[1], Jonathan H. Epstein[1,2], Jacqueline Weyer[1,4,5], Janusz T. Paweska[1,4,5] and Wanda Markotter[1]*

*Corresponding author: wanda.markotter@up.ac.za

This PDF file includes:

- Tables S1 – S2
- Figures S1 – S2
- Legends for Datasets S1 – S2
- Datasets S1 and S2

Table S1: GAMM Model Summary.

Table S1A: GAMM Intercepts Summary

Multinomial Outcome	Model Term	Effect Type	Description	Effect	p-value
RouAlphaCoV	(Intercept)	Intercept	Prevalence at mean covariate values	-3.9 (-4.38, -3.42)	0***
HKU9-related Beta-CoV	(Intercept).1	Intercept	Prevalence at mean covariate values	-2.4 (-2.7, -2.1)	0***
RouNobeCoV	(Intercept).2	Intercept	Prevalence at mean covariate values	-6 (-7.34, -4.66)	0***

Table S1B: GAMM Smooths Summary

Model Term	Effect Type	Description	Knots/ Levels	Effective Degrees of Freedom	p-value	Minimum Effect Size	Maximum Effect Size	Evaluated for Rectal Samples Only	Evaluated on Dates with Bat Collection Only
Multinomial Outcome: RouAlphaCoV									
s(sample_type):dummy_rectal1	Random Effect	Random effect fecal or rectal baseline prevalence differing from intercept	2	0.00	0.5156	-0.00019 (-0.013, 0.013)	0 (0, 0)	Yes	Yes
s(day):sample_typeFecal	Thin Plate Spline	Long-term nonlinear trend of prevalence in fecal samples	5	2.57	0.1733	-1.2 (-3.4, 0.76)	2.4 (-0.28, 5)	No	No
s(day):sample_typeRectal	Thin Plate Spline	Long-term nonlinear trend of prevalence in rectal samples	5	1.00	0.6887	-0.15 (-1, 0.85)	0.15 (-0.85, 1)	Yes	Yes
s(day,gender_age):dummy_rectal1	Factor-Spline	Random nonlinear effects of trends in prevalence within gender/age groups varying from the common long-term trend	25	0.00	0.9056	-2e-04 (-0.0084, 0.0087)	0.00034 (-0.0085, 0.0087)	Yes	Yes
s(day_of_year):sample_typeFecal	Cyclic Cubic Spline	Annual nonlinear cyclic trend of prevalence in fecal samples	5	2.25	0.0000***	-2.6 (-4, -1.3)	1.9 (1, 2.6)	No	No

Model Term	Effect Type	Description	Knots/ Levels	Effective Degrees of Freedom	p-value	Minimum Effect Size	Maximum Effect Size	Evaluated for Rectal Samples Only	Evaluated on Dates with Bat Collection Only
s(day_of_year):sample_typeRectal	Cyclic Cubic Spline	Annual nonlinear cyclic trend of prevalence in rectal samples	5	1.35	0.0484*	-0.72 (-1.4, -0.027)	0.56 (-0.016, 1.1)	Yes	Yes
s(day_of_year,gender_age):dummy_rectal1	Factor-Spline	Random nonlinear effects of trends in prevalence within gender/age groups varying from the common annual cyclic trend	20	0.00	0.9000	-6e-04 (-0.0096, 0.0089)	0.00078 (-0.007, 0.0085)	Yes	Yes
s(fmi_normalized):dummy_rectal1	Thin Plate Spline	Nonlinear effect of bat FMI, normalized within age, sex, and reproductive calss, on prevalence	5	1.00	0.0744·	-2 (-3.9, 0.13)	2.4 (-0.15, 4.7)	Yes	Yes
s(reproductive_condition):dummy_repro1	Random Effect	Random effect of prevalence in bats with reproductive conditions (Pregnant, Lactating, Scrotal) varying from intercept	4	0.00	0.9113	-0.00059 (-0.013, 0.013)	0.00029 (-0.014, 0.015)	Yes	Yes
Multinomial Outcome: HKU9-related BetaCoV									
s.1(sample_type):dummy_rectal1	Random Effect	Random effect fecal or rectal baseline prevalence differing from intercept	2	0.00	0.4588	-7.7e-05 (-0.0055, 0.0052)	0 (0, 0)	No	No
s.1(day):sample_typeFecal	Thin Plate Spline	Long-term nonlinear trend of prevalence in fecal samples	5	2.34	0.0015**	-1.3 (-2, -0.5)	0.66 (0.29, 1.1)	No	No
s.1(day):sample_typeRectal	Thin Plate Spline	Long-term nonlinear trend of prevalence in rectal samples	5	2.63	0.0558·	-0.69 (-1.3, 0.0096)	0.8 (-0.35, 1.7)	Yes	Yes
s.1(day,gender_age):dummy_rectal1	Factor-Spline	Random nonlinear effects of trends in prevalence within gender/age groups varying	25	0.00	0.8664	-0.00023 (-0.0048, 0.005)	0.00014 (-0.0045, 0.0047)	Yes	Yes

Model Term	Effect Type	Description	Knots/ Levels	Effective Degrees of Freedom	p-value	Minimum Effect Size	Maximum Effect Size	Evaluated for Rectal Samples Only	Evaluated on Dates with Bat Collection Only
		from the common long-term trend							
s.1(day_of_year):sample_typeFecal	Cyclic Cubic Spline	Annual nonlinear cyclic trend of prevalence in fecal samples	5	2.41	0.0000***	-1.5 (-2.2, -0.8)	1.4 (0.95, 1.9)	No	No
s.1(day_of_year):sample_typeRectal	Cyclic Cubic Spline	Annual nonlinear cyclic trend of prevalence in rectal samples	5	2.07	0.0005***	-0.59 (-1.4, 0.09)	0.8 (0.22, 1.4)	Yes	Yes
s.1(day_of_year,gender_age):dummy_rectal1	Factor-Spline	Random nonlinear effects of trends in prevalence within gender/age groups varying from the common annual cyclic trend	20	0.00	0.8028	-0.00029 (-0.0075, 0.0071)	4e-04 (-0.0068, 0.0077)	Yes	Yes
s.1(fmi_normalized):dummy_rectal1	Thin Plate Spline	Nonlinear effect of bat FMI, normalized within age, sex, and reproductive class, on prevalence	5	1.00	0.1651	-0.92 (-2, 0.2)	1.1 (-0.27, 2.4)	Yes	Yes
s.1(reproductive_condition):dummy_repro1	Random Effect	Random effect of prevalence in bats with reproductive conditions (Pregnant, Lactating, Scrotal) varying from intercept	4	1.83	0.0254*	-1.6 (-3, -0.36)	0 (0, 0)	Yes	Yes
Multinomial Outcome: RouNobeCoV									
s.2(sample_type):dummy_rectal1	Random Effect	Random effect fecal or rectal baseline prevalence differing from intercept	2	0.00	0.7581	-3.3e-05 (-0.01, 0.01)	0 (0, 0)	Yes	Yes
s.2(day):sample_typeFecal	Thin Plate Spline	Long-term nonlinear trend of prevalence in fecal samples	5	1.00	0.9236	-0.045 (-1.6, 1.6)	0.029 (-1, 1)	No	No

Model Term	Effect Type	Description	Knots/ Levels	Effective Degrees of Freedom	p-value	Minimum Effect Size	Maximum Effect Size	Evaluated for Rectal Samples Only	Evaluated on Dates with Bat Collection Only
s.2(day):sample_typeRectal	Thin Plate Spline	Long-term nonlinear trend of prevalence in rectal samples	5	1.00	0.5572	-1.2 (-4.6, 1.9)	1.2 (-1.9, 4.5)	Yes	Yes
s.2(day_of_year):sample_typeFecal	Cyclic Cubic Spline	Annual nonlinear cyclic trend of prevalence in fecal samples	5	2.07	0.0000***	-3.3 (-4.6, -1.8)	4.3 (2.4, 6.1)	No	No
s.2(day_of_year):sample_typeRectal	Cyclic Cubic Spline	Annual nonlinear cyclic trend of prevalence in rectal samples	5	1.48	0.0363*	-1.6 (-2.9, -0.25)	2.2 (0.53, 3.8)	Yes	Yes

Table S2: Body Condition and Prevalence by demographic group (mean, median and range).

Subgroup	FMI (kg/m^2)	Mass (g)	Forearm (mm)	CoV prevalence
All Sampled Bats	12.3, 12, (5.72-20.3)	95.2, 87, (45-179)	87.4, 86.6, (71.1-101)	13.2% (10.8%-16.0%)
Adults (overall)	13.6, 13.4, (5.72-20.3)	119, 120, (51-179)	93.4, 93.2, (84.9-101)	6.6% (3.9%-10.3%)
Subadults (overall)	11.6, 11.6, (5.88-16.7)	81.7, 81, (45-127)	84, 84, (71.1-88.8)	17.1% (13.7%-20.9%)
Subadult – females	11.5, 11.4, (6.82-16.7)	80.8, 80, (51-127)	83.8, 83.6, (71.1-88.8)	16.7% (12.1%-22.2%)
Subadult – males	11.6, 11.7, (5.88-14.6)	82.7, 83, (45-114)	84.2, 84.5, (71.3-88.8)	17.4% (12.7%-23.0%)
Adult – female (non-pregnant/ lactating)	11.7, 12.1, (6.51-14.7)	101, 102, (53-134)	92.6, 92.2, (89-99.1)	14.3% (7.1%-24.7%)
Adult – female (lactating)	16.9, 16.8, (13.1-20.3)	147, 147, (121-179)	93.4, 93, (89.5-98.2)	0.0% (0.0%-21.8%)
Adults – female (pregnant)	14.7, 14.2, (8.71-19.2)	129, 126, (74-175)	93.5, 93.6, (88.6-101)	5.9% (1.2%-16.2%)
Adult – male (non-scrotal)	12, 12, (7.23-15.7)	100, 98, (69-153)	91.5, 90.9, (84.9-99.1)	5.1% (0.6%-17.3%)
Adult – males (scrotal)	14.5, 14.5, (5.72-17.8)	131, 133, (51-164)	95, 94.9, (89.9-101)	2.4% (0.3%-8.3%)

* FMI units from Meng et al. 2016, where bat mass in kg are divided by the forearm length in meters (squared) to provide a FMI ratio.

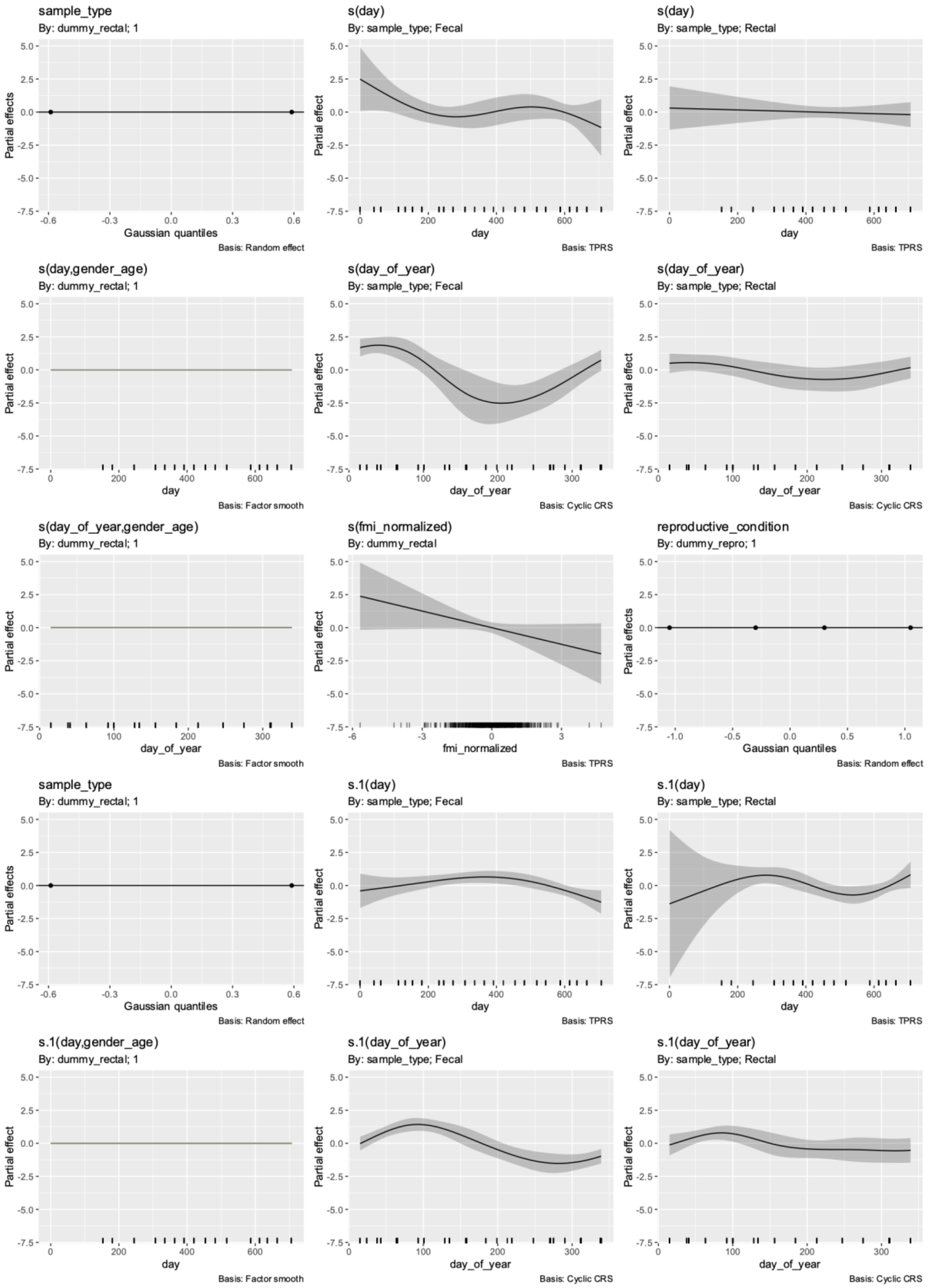

Figure S1: GAMM partial effects plots: Each plot shows the partial effect on the linear (estimation) scale of a single term in the multinomial GAMM. Note y-axis scales vary. Ticks on axis indicate variable data values for continuous variables. Random effects are plotted as Gaussian quantile plots. Smooth-factor interactions (hierarchical splines) show deviation of categorical splines from the overall trend. For single splines, shaded areas represent 95% Nychka (1988)-type intervals. Refer to Table S1 for description and significance of individual terms.

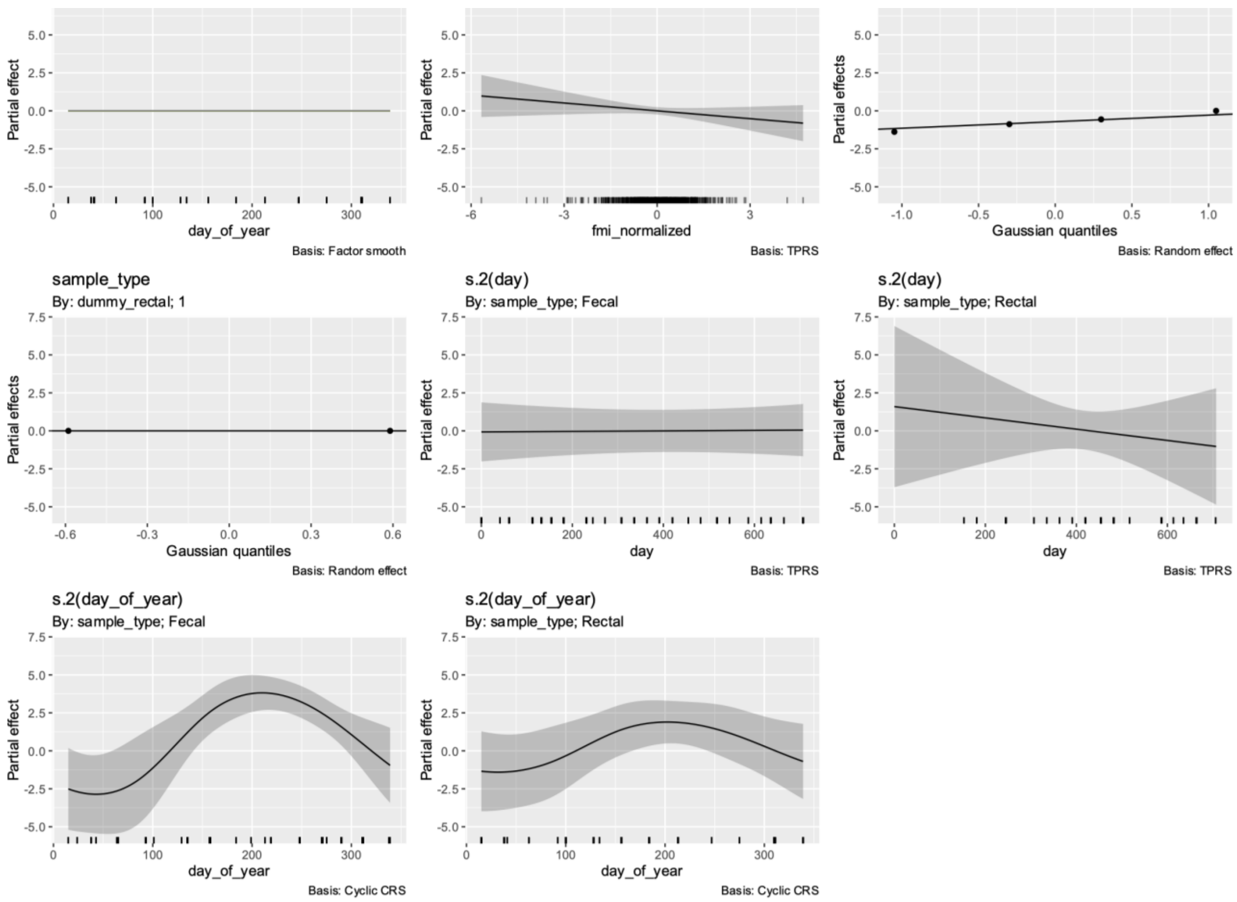

Figure S1 continued.

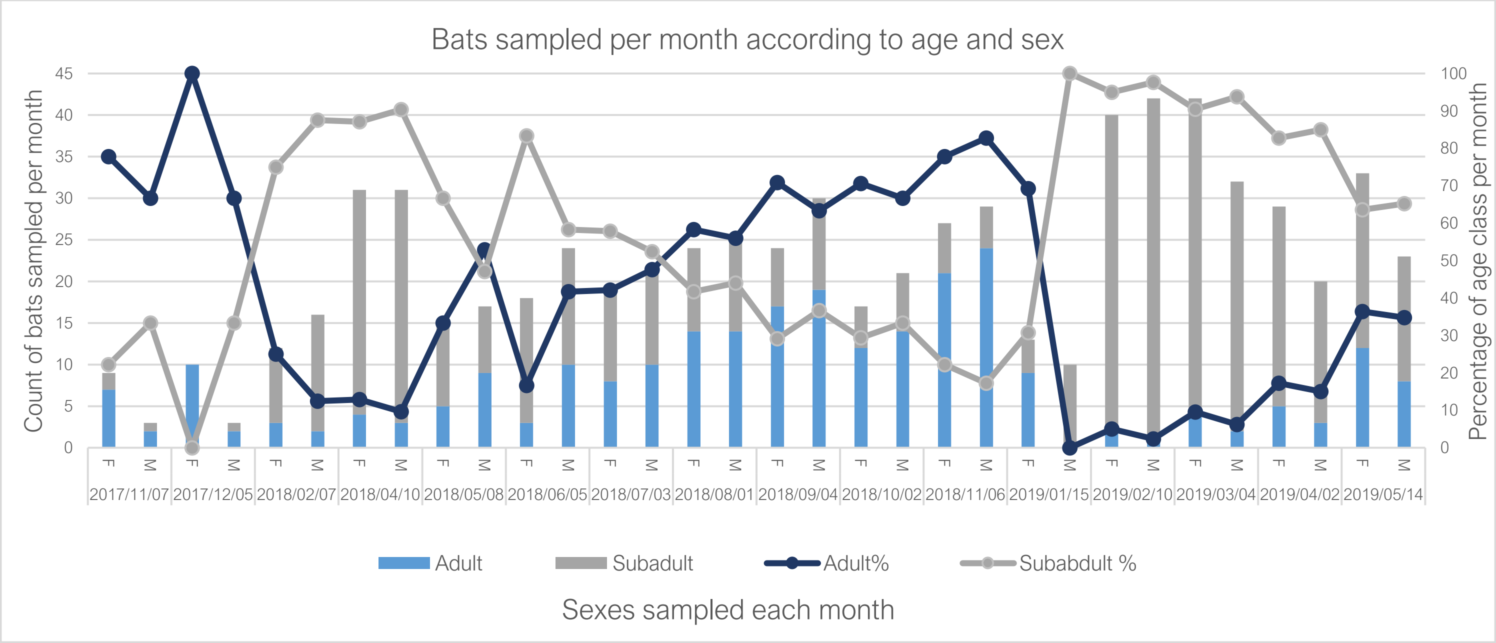

Figure S2: Summary of bat demographics (age and sex) sampled per month. The axis for bar graphs are represented on the left, depicting counts of bats sampled per month. The axis for the

line graph is on the right and depict the age class per sex according to the total of that class sampled per month as a percentage.

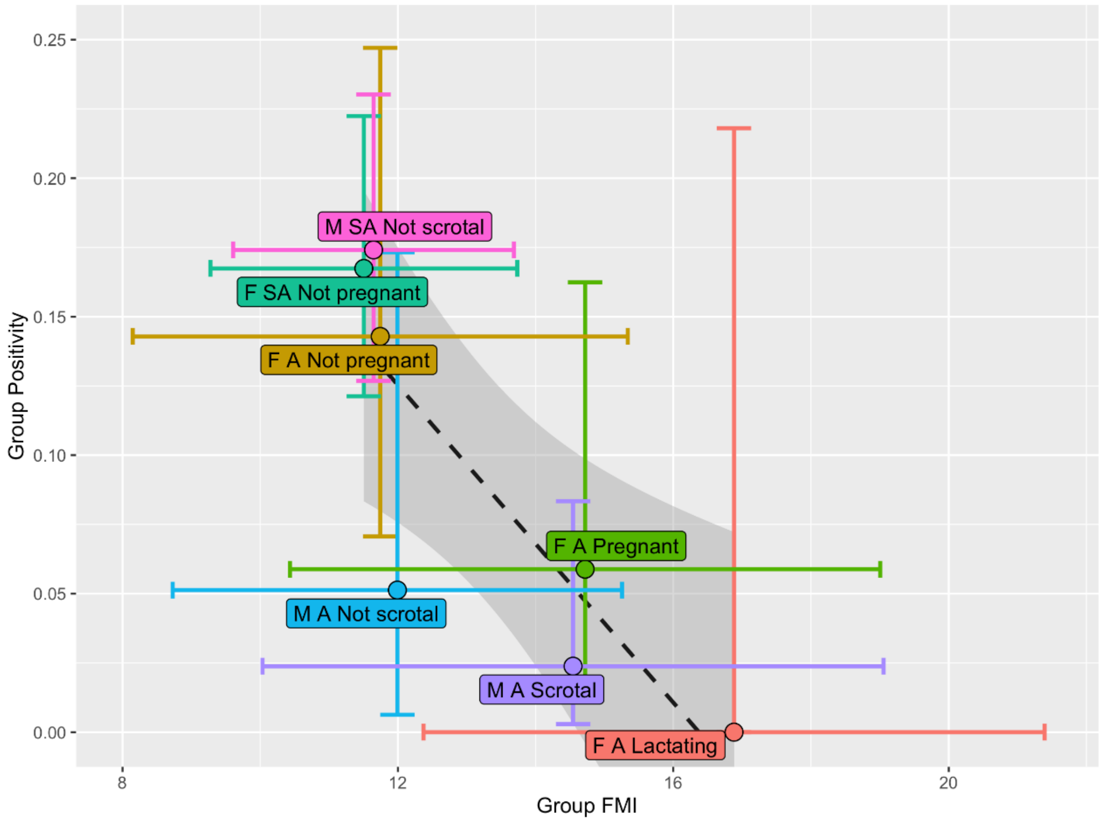

Figure S3: Co-variation of FMI with age, sex, and reproductive condition. Points and error bars represent mean and 95% intervals of FMI and seroprevalence amongst bats within demographic subgroups. Subgroup labels are F - Female, M - Male, A - Adult, SA - Subadult. Line and intervals represent simple linear trends among group means.

Dataset S1: Sampling information for fecal pools with accession numbers.

Dataset S2: Data for individual *Rousettus aegyptiacus* with accession numbers.

Dataset 1: Sample information of population fecal pools

Sample number	Sample type	Date collected	Coronavirus detected	Genus	Clade	Accession number	Strain
UPE 060	Faecal pool (x3)	7/6/2017	0				
UPE 061	Faecal pool (x3)	7/6/2017	0				
UPE 062	Faecal pool (x3)	7/6/2017	0				
UPE 063	Faecal pool (x3)	7/6/2017	0				
UPE 064	Faecal pool (x3)	7/6/2017	0				
UPE 065	Faecal pool (x3)	7/6/2017	0				
UPE 066	Faecal pool (x3)	7/6/2017	0				
UPE 067	Faecal pool (x3)	7/6/2017	0				
UPE 068	Faecal pool (x3)	7/6/2017	0				
UPE 069	Faecal pool (x3)	7/6/2017	0				
UPE 070	Faecal pool (x3)	7/6/2017	0				
**UPE 071**	Faecal pool (x3)	**7/6/2017**	**1**	**beta**	**2**	MZ547505	BtCoV/Rousettus/LP/RSA/UPE71/2017
**UPE 072**	Faecal pool (x3)	**7/6/2017**	**1**	**alpha**	**1**	MZ547456	BtCoV/Rousettus/LP/RSA/UPE72/2017
UPE 073	Faecal pool (x3)	7/6/2017	0				
UPE 074	Faecal pool (x3)	7/6/2017	0				
UPE 075	Faecal pool (x3)	7/6/2017	0				
UPE 076	Faecal pool (x3)	7/6/2017	0				
UPE 077	Faecal pool (x3)	7/6/2017	0				
UPE 101	Faecal pool (x3)	18/7/2017	0				
UPE 102	Faecal pool (x3)	18/7/2017	0				
UPE 103	Faecal pool (x3)	18/7/2017	0				
UPE 104	Faecal pool (x3)	18/7/2017	0				
**UPE 105**	Faecal pool (x3)	**18/7/2017**	**1**	**beta**	**3**	MZ547492	BtCoV/Rousettus/LP/RSA/UPE105/2017
UPE 106	Faecal pool (x3)	18/7/2017	0				
**UPE 107**	Faecal pool (x3)	**18/7/2017**	**1**	**beta**	**3**	MZ547493	BtCoV/Rousettus/LP/RSA/UPE107/2017
UPE 108	Faecal pool (x3)	18/7/2017	0				
UPE 109	Faecal pool (x3)	18/7/2017	0				
**UPE 110**	Faecal pool (x3)	**18/7/2017**	**1**	**beta**	**2**	MZ547506	BtCoV/Rousettus/LP/RSA/UPE110/2017
UPE 129	Faecal pool (x3)	18/7/2017	0				
UPE 134	Faecal pool (x3)	7/8/2017	0				
**UPE 135**	Faecal pool (x3)	**7/8/2017**	**1**	**beta**	**2**	MZ547507	BtCoV/Rousettus/LP/RSA/UPE135/2017
UPE 137	Faecal pool (x3)	7/8/2017	0				
UPE 138	Faecal pool (x3)	7/8/2017	0				
UPE 139	Faecal pool (x3)	7/8/2017	0				
UPE 140	Faecal pool (x3)	7/8/2017	0				
UPE 141	Faecal pool (x3)	7/8/2017	0				
UPE 142	Faecal pool (x3)	7/8/2017	0				
UPE 143	Faecal pool (x3)	7/8/2017	0				
UPE 144	Faecal pool (x3)	7/8/2017	0				
UPE 145	Faecal pool (x3)	7/8/2017	0				
UPE 146	Faecal pool (x3)	7/8/2017	0				
UPE 148	Faecal pool (x3)	7/8/2017	0				
**UPE 149**	Faecal pool (x3)	**7/8/2017**	**1**	**beta**	**3**	MZ547494	BtCoV/Rousettus/LP/RSA/UPE149/2017
UPE 150	Faecal pool (x3)	7/8/2017	0				
UPE 151	Faecal pool (x3)	7/8/2017	0				
**UPE 152**	Faecal pool (x3)	**7/8/2017**	**1**	**beta**	**3**	MZ547495	BtCoV/Rousettus/LP/RSA/UPE152/2017
UPE 153	Faecal pool (x3)	7/8/2017	0				
UPE 154	Faecal pool (x3)	7/8/2017	0				
UPE 155	Faecal pool (x3)	7/8/2017	0				
UPE 156	Faecal pool (x3)	7/8/2017	0				
UPE 157	Faecal pool (x3)	7/8/2017	0				
UPE 158	Faecal pool (x3)	7/8/2017	0				
UPE 159	Faecal pool (x3)	7/8/2017	0				
UPE 160	Faecal pool (x3)	7/8/2017	0				
**UPE 205**	Faecal pool (x3)	**27/9/2017**	**1**	**beta**	**3**	MZ547496	BtCoV/Rousettus/LP/RSA/UPE205/2017
UPE 206	Faecal pool (x3)	27/9/2017	0				
UPE 207	Faecal pool (x3)	27/9/2017	0				
UPE 208	Faecal pool (x3)	27/9/2017	0				
UPE 210	Faecal pool (x3)	27/9/2017	0				
UPE 211	Faecal pool (x3)	27/9/2017	0				
**UPE 212**	Faecal pool (x3)	**27/9/2017**	**1**	**alpha**	**1**	MZ547450	BtCoV/Rousettus/LP/RSA/UPE212/2017
UPE 213	Faecal pool (x3)	27/9/2017	0				
UPE 214	Faecal pool (x3)	27/9/2017	0				
UPE 215	Faecal pool (x3)	27/9/2017	0				
UPE 216	Faecal pool (x3)	27/9/2017	0				
UPE 217	Faecal pool (x3)	27/9/2017	0				
UPE 218	Faecal pool (x3)	27/9/2017	0				
UPE 219	Faecal pool (x3)	27/9/2017	0				
UPE 220	Faecal pool (x3)	27/9/2017	0				
UPE 221	Faecal pool (x3)	27/9/2017	0				
UPE 222	Faecal pool (x3)	27/9/2017	0				
UPE 223	Faecal pool (x3)	27/9/2017	0				
UPE 224	Faecal pool (x3)	27/9/2017	0				
UPE 225	Faecal pool (x3)	27/9/2017	0				
UPE 226	Faecal pool (x3)	28/9/2017	0				
UPE 227	Faecal pool (x3)	28/9/2017	0				

UPE 228	Faecal pool (x3)	28/9/2017	0				
UPE 229	Faecal pool (x3)	28/9/2017	0				
UPE 230	Faecal pool (x3)	28/9/2017	0				
UPE 231	Faecal pool (x3)	28/9/2017	0				
UPE 232	Faecal pool (x3)	28/9/2017	0				
UPE 233	Faecal pool (x3)	28/9/2017	0				
UPE 234	Faecal pool (x3)	28/9/2017	0				
UPE 235	Faecal pool (x3)	28/9/2017	0				
UPE 236	Faecal pool (x3)	28/9/2017	0				
UPE 237	Faecal pool (x3)	28/9/2017	0				
UPE 239	Faecal pool (x3)	28/9/2017	0				
UPE 240	Faecal pool (x3)	28/9/2017	0				
UPE 241	Faecal pool (x3)	28/9/2017	0				
UPE 242	Faecal pool (x3)	28/9/2017	0				
UPE 243	Faecal pool (x3)	28/9/2017	0				
UPE 244	Faecal pool (x3)	28/9/2017	0				
UPE 245	Faecal pool (x3)	28/9/2017	0				
UPE 246	Faecal pool (x3)	28/9/2017	0				
UPE 247	Faecal pool (x3)	28/9/2017	0				
**UPE 274**	Faecal pool (x3)	**17/10/2017**	**1**	**beta**	**2**	MZ547508	BtCoV/Rousettus/LP/RSA/UPE274/2017
UPE 275	Faecal pool (x3)	17/10/2017	0				
UPE 276	Faecal pool (x3)	17/10/2017	0				
UPE 277	Faecal pool (x3)	17/10/2017	0				
UPE 278	Faecal pool (x3)	17/10/2017	0				
**UPE 279**	Faecal pool (x3)	**17/10/2017**	**1**	**alpha**	**1**	MZ547455	BtCoV/Rousettus/LP/RSA/UPE279/2017
UPE 280	Faecal pool (x3)	17/10/2017	0				
UPE 281	Faecal pool (x3)	17/10/2017	0				
UPE 282	Faecal pool (x3)	17/10/2017	0				
UPE 283	Faecal pool (x3)	17/10/2017	0				
UPE 284	Faecal pool (x3)	17/10/2017	0				
UPE 285	Faecal pool (x3)	17/10/2017	0				
UPE 286	Faecal pool (x3)	17/10/2017	0				
**UPE 287**	Faecal pool (x3)	**17/10/2017**	**1**	**beta**	**2**	MZ547509	BtCoV/Rousettus/LP/RSA/UPE287/2017
UPE 288	Faecal pool (x3)	17/10/2017	0				
UPE 289	Faecal pool (x3)	17/10/2017	0				
UPE 291	Faecal pool (x3)	17/10/2017	0				
UPE 292	Faecal pool (x3)	17/10/2017	0				
UPE 293	Faecal pool (x3)	17/10/2017	0				
UPE 294	Faecal pool (x3)	17/10/2017	0				
UPE 295	Faecal pool (x3)	17/10/2017	0				
UPE 296	Faecal pool (x3)	17/10/2017	0				
UPE 298	Faecal pool (x3)	17/10/2017	0				
UPE 299	Faecal pool (x3)	17/10/2017	0				
UPE 300	Faecal pool (x3)	17/10/2017	0				
UPE 301	Faecal pool (x3)	17/10/2017	0				
UPE 302	Faecal pool (x3)	17/10/2017	0				
UPE 303	Faecal pool (x3)	17/10/2017	0				
UPE 304	Faecal pool (x3)	17/10/2017	0				
UPE 305	Faecal pool (x3)	17/10/2017	0				
UPE 306	Faecal pool (x3)	17/10/2017	0				
UPE 307	Faecal pool (x3)	17/10/2017	0				
UPE 308	Faecal pool (x3)	17/10/2017	0				
UPE 309	Faecal pool (x3)	17/10/2017	0				
UPE 310	Faecal pool (x3)	17/10/2017	0				
UPE 311	Faecal pool (x3)	17/10/2017	0				
UPE 312	Faecal pool (x3)	17/10/2017	0				
UPE 368	Faecal pool (x3)	8/11/2017	0				
UPE 369	Faecal pool (x3)	8/11/2017	0				
UPE 370	Faecal pool (x3)	8/11/2017	0				
UPE 371	Faecal pool (x3)	8/11/2017	0				
UPE 372	Faecal pool (x3)	8/11/2017	0				
UPE 373	Faecal pool (x3)	8/11/2017	0				
UPE 374	Faecal pool (x3)	8/11/2017	0				
UPE 375	Faecal pool (x3)	8/11/2017	0				
UPE 376	Faecal pool (x3)	8/11/2017	0				
UPE 377	Faecal pool (x3)	8/11/2017	0				
UPE 378	Faecal pool (x3)	8/11/2017	0				
UPE 379	Faecal pool (x3)	8/11/2017	0				
UPE 380	Faecal pool (x3)	8/11/2017	0				
UPE 381	Faecal pool (x3)	8/11/2017	0				
UPE 382	Faecal pool (x3)	8/11/2017	0				
UPE 383	Faecal pool (x3)	8/11/2017	0				
UPE 384	Faecal pool (x3)	8/11/2017	0				
UPE 385	Faecal pool (x3)	8/11/2017	0				
**UPE 386**	Faecal pool (x3)	**8/11/2017**	**1**	**alpha**	**1**	MZ547457	BtCoV/Rousettus/LP/RSA/UPE386/2017
UPE 387	Faecal pool (x3)	8/11/2017	0				
UPE 388	Faecal pool (x3)	8/11/2017	0				
UPE 389	Faecal pool (x3)	8/11/2017	0				
UPE 391	Faecal pool (x3)	8/11/2017	0				

UPE 392	Faecal pool (x3)	8/11/2017	0				
UPE 393	Faecal pool (x3)	8/11/2017	0				
UPE 394	Faecal pool (x3)	8/11/2017	0				
UPE 395	Faecal pool (x3)	8/11/2017	0				
UPE 396	Faecal pool (x3)	8/11/2017	0				
UPE 397	Faecal pool (x3)	8/11/2017	0				
UPE 398	Faecal pool (x3)	8/11/2017	0				
UPE 399	Faecal pool (x3)	8/11/2017	0				
UPE 400	Faecal pool (x3)	8/11/2017	0				
UPE 401	Faecal pool (x3)	8/11/2017	0				
UPE 402	Faecal pool (x3)	8/11/2017	0				
UPE 403	Faecal pool (x3)	8/11/2017	0				
UPE 404	Faecal pool (x3)	8/11/2017	0				
UPE 405	Faecal pool (x3)	8/11/2017	0				
UPE 425	Faecal pool (x3)	5/12/2017	0				
UPE 426	Faecal pool (x3)	5/12/2017	0				
UPE 427	Faecal pool (x3)	5/12/2017	0				
UPE 428	Faecal pool (x3)	5/12/2017	0				
UPE 429	Faecal pool (x3)	5/12/2017	0				
UPE 430	Faecal pool (x3)	5/12/2017	0				
UPE 431	Faecal pool (x3)	5/12/2017	0				
UPE 432	Faecal pool (x3)	5/12/2017	0				
UPE 433	Faecal pool (x3)	5/12/2017	0				
UPE 434	Faecal pool (x3)	5/12/2017	0				
UPE 435	Faecal pool (x3)	5/12/2017	0				
UPE 436	Faecal pool (x3)	5/12/2017	0				
UPE 437	Faecal pool (x3)	5/12/2017	0				
UPE 438	Faecal pool (x3)	5/12/2017	0				
UPE 439	Faecal pool (x3)	5/12/2017	0				
**UPE 440**	Faecal pool (x3)	**5/12/2017**	**1**	**alpha**	**1**	MZ547454	BtCoV/Rousettus/LP/RSA/UPE440/2017
UPE 441	Faecal pool (x3)	5/12/2017	0				
UPE 442	Faecal pool (x3)	5/12/2017	0				
UPE 443	Faecal pool (x3)	5/12/2017	0				
UPE 444	Faecal pool (x3)	5/12/2017	0				
UPE 445	Faecal pool (x3)	5/12/2017	0				
UPE 446	Faecal pool (x3)	5/12/2017	0				
UPE 447	Faecal pool (x3)	5/12/2017	0				
UPE 448	Faecal pool (x3)	5/12/2017	0				
UPE 449	Faecal pool (x3)	5/12/2017	0				
UPE 450	Faecal pool (x3)	5/12/2017	0				
UPE 451	Faecal pool (x3)	5/12/2017	0				
UPE 452	Faecal pool (x3)	5/12/2017	0				
UPE 453	Faecal pool (x3)	5/12/2017	0				
UPE 454	Faecal pool (x3)	5/12/2017	0				
UPE 455	Faecal pool (x3)	5/12/2017	0				
UPE 456	Faecal pool (x3)	5/12/2017	0				
UPE 457	Faecal pool (x3)	5/12/2017	0				
UPE 458	Faecal pool (x3)	5/12/2017	0				
UPE 459	Faecal pool (x3)	5/12/2017	0				
UPE 490	Faecal pool (x3)	5/12/2017	0				
UPE 491	Faecal pool (x3)	5/12/2017	0				
**UPE 492**	Faecal pool (x3)	**5/12/2017**	**1**	**beta**	**2**	MZ547510	BtCoV/Rousettus/LP/RSA/UPE492/2017
UPE 493	Faecal pool (x3)	5/12/2017	0				
UPE 494	Faecal pool (x3)	5/12/2017	0				
UPE 495	Faecal pool (x3)	5/12/2017	0				
UPE 496	Faecal pool (x3)	5/12/2017	0				
UPE 497	Faecal pool (x3)	5/12/2017	0				
UPE 498	Faecal pool (x3)	5/12/2017	0				
UPE 499	Faecal pool (x3)	5/12/2017	0				
UPE 538	Faecal pool (x3)	24/1/2018	0				
UPE 539	Faecal pool (x3)	24/1/2018	0				
UPE 540	Faecal pool (x3)	24/1/2018	0				
UPE 541	Faecal pool (x3)	24/1/2018	0				
UPE 542	Faecal pool (x3)	24/1/2018	0				
UPE 543	Faecal pool (x3)	24/1/2018	0				
**UPE 544**	Faecal pool (x3)	**24/1/2018**	**1**	**alpha**	**1**	MZ547471	BtCoV/Rousettus/LP/RSA/UPE544/2018
UPE 545	Faecal pool (x3)	24/1/2018	0				
UPE 546	Faecal pool (x3)	24/1/2018	0				
UPE 547	Faecal pool (x3)	24/1/2018	0				
UPE 548	Faecal pool (x3)	24/1/2018	0				
UPE 549	Faecal pool (x3)	24/1/2018	0				
UPE 550	Faecal pool (x3)	24/1/2018	0				
UPE 551	Faecal pool (x3)	24/1/2018	0				
UPE 552	Faecal pool (x3)	24/1/2018	0				
**UPE 553**	Faecal pool (x3)	**24/1/2018**	**1**	**beta**	**4**	**-**	**Rodent CoV**
UPE 554	Faecal pool (x3)	24/1/2018	0				
UPE 555	Faecal pool (x3)	24/1/2018	0				
**UPE 556**	Faecal pool (x3)	**24/1/2018**	0				
UPE 557	Faecal pool (x3)	24/1/2018	0				

UPE 558	Faecal pool (x3)	24/1/2018	0				
UPE 559	Faecal pool (x3)	24/1/2018	0				
UPE 560	Faecal pool (x3)	24/1/2018	0				
UPE 561	Faecal pool (x3)	24/1/2018	0				
UPE 562	Faecal pool (x3)	24/1/2018	0				
UPE 563	Faecal pool (x3)	24/1/2018	0				
UPE 564	Faecal pool (x3)	24/1/2018	0				
UPE 565	Faecal pool (x3)	24/1/2018	0				
UPE 566	Faecal pool (x3)	24/1/2018	**1**	**alpha**	**1**	MZ547458	BtCoV/Rousettus/LP/RSA/UPE566/2018
UPE 567	Faecal pool (x3)	24/1/2018	0				
UPE 568	Faecal pool (x3)	24/1/2018	0				
UPE 569	Faecal pool (x3)	24/1/2018	0				
UPE 570	Faecal pool (x3)	24/1/2018	0				
UPE 571	Faecal pool (x3)	24/1/2018	0				
UPE 572	Faecal pool (x3)	24/1/2018	0				
UPE 573	Faecal pool (x3)	24/1/2018	0				
UPE 574	Faecal pool (x3)	24/1/2018	0				
UPE 631	Faecal pool (x3)	7/2/2018	0				
UPE 632	Faecal pool (x3)	7/2/2018	0				
**UPE 633**	Faecal pool (x3)	**7/2/2018**	**1**	**beta**	**2**	MZ547511	BtCoV/Rousettus/LP/RSA/UPE633/2018
UPE 634	Faecal pool (x3)	7/2/2018	0				
UPE 635	Faecal pool (x3)	7/2/2018	0				
UPE 636	Faecal pool (x3)	7/2/2018	0				
UPE 637	Faecal pool (x3)	7/2/2018	0				
UPE 638	Faecal pool (x3)	7/2/2018	0				
**UPE 639**	Faecal pool (x3)	**7/2/2018**	**1**	**beta**	**2**	MZ547512	BtCoV/Rousettus/LP/RSA/UPE639/2018
UPE 640	Faecal pool (x3)	7/2/2018	0				
**UPE 641**	Faecal pool (x3)	**7/2/2018**	**1**	**beta**	**2**	MZ547576	BtCoV/Rousettus/LP/RSA/UPE641/2018
UPE 642	Faecal pool (x3)	7/2/2018	0				
UPE 643	Faecal pool (x3)	7/2/2018	0				
UPE 644	Faecal pool (x3)	7/2/2018	0				
**UPE 645**	Faecal pool (x3)	**7/2/2018**	**1**	**beta**	**2**	MZ547513	BtCoV/Rousettus/LP/RSA/UPE645/2018
UPE 646	Faecal pool (x3)	7/2/2018	0				
**UPE 647**	Faecal pool (x3)	**7/2/2018**	**1**	**beta**	**2**	MZ547514	BtCoV/Rousettus/LP/RSA/UPE647/2018
UPE 648	Faecal pool (x3)	7/2/2018	0				
UPE 649	Faecal pool (x3)	7/2/2018	0				
UPE 650	Faecal pool (x3)	7/2/2018	0				
**UPE 651**	Faecal pool (x3)	**7/2/2018**	**1**	**alpha**	**1**	MZ547472	BtCoV/Rousettus/LP/RSA/UPE651/2018
UPE 652	Faecal pool (x3)	7/2/2018	0				
UPE 653	Faecal pool (x3)	7/2/2018	0				
**UPE 654**	Faecal pool (x3)	**7/2/2018**	**1**	**beta**	**2**	MZ547515	BtCoV/Rousettus/LP/RSA/UPE654/2018
UPE 655	Faecal pool (x3)	7/2/2018	0				
**UPE 656**	Faecal pool (x3)	**7/2/2018**	**1**	**beta**	**2**	MZ547516	BtCoV/Rousettus/LP/RSA/UPE656/2018
**UPE 657**	Faecal pool (x3)	**7/2/2018**	**1**	**beta**	**2**	MZ547517	BtCoV/Rousettus/LP/RSA/UPE657/2018
UPE 658	Faecal pool (x3)	7/2/2018	0				
UPE 659	Faecal pool (x3)	7/2/2018	0				
UPE 660	Faecal pool (x3)	7/2/2018	0				
**UPE 661**	Faecal pool (x3)	**7/2/2018**	**1**	**beta**	**2**	MZ547518	BtCoV/Rousettus/LP/RSA/UPE661/2018
UPE 662	Faecal pool (x3)	7/2/2018	0				
UPE 663	Faecal pool (x3)	7/2/2018	0				
UPE 664	Faecal pool (x3)	7/2/2018	0				
UPE 665	Faecal pool (x3)	7/2/2018	0				
UPE 666	Faecal pool (x3)	7/2/2018	0				
UPE 667	Faecal pool (x3)	7/2/2018	0				
UPE 668	Faecal pool (x3)	7/2/2018	0				
**UPE 669**	Faecal pool (x3)	**7/2/2018**	**1**	**beta**	**2**	MZ547519	BtCoV/Rousettus/LP/RSA/UPE669/2018
UPE 691	Faecal pool (x3)	6/3/2018	0				
**UPE 692**	Faecal pool (x3)	**6/3/2018**	**1**	**beta**	**2**	MZ547520	BtCoV/Rousettus/LP/RSA/UPE692/2018
UPE 693	Faecal pool (x3)	6/3/2018	0				
UPE 694	Faecal pool (x3)	6/3/2018	0				
UPE 695	Faecal pool (x3)	6/3/2018	0				
UPE 696	Faecal pool (x3)	6/3/2018	0				
UPE 697	Faecal pool (x3)	6/3/2018	0				
**UPE 698**	Faecal pool (x3)	**6/3/2018**	**1**	**alpha**	**1**	MZ547453	BtCoV/Rousettus/LP/RSA/UPE698/2018
UPE 699	Faecal pool (x3)	6/3/2018	0				
UPE 700	Faecal pool (x3)	6/3/2018	0				
UPE 701	Faecal pool (x3)	6/3/2018	0				
**UPE 702**	Faecal pool (x3)	**6/3/2018**	**1**	**beta**	**2**	MZ547648	BtCoV/Rousettus/LP/RSA/UPE702/2018
**UPE 703**	Faecal pool (x3)	**6/3/2018**	**1**	**beta**	**2**	MZ547521	BtCoV/Rousettus/LP/RSA/UPE703/2018
**UPE 704**	Faecal pool (x3)	**6/3/2018**	**1**	**alpha**	**1**	MZ547473	BtCoV/Rousettus/LP/RSA/UPE704/2018
UPE 705	Faecal pool (x3)	6/3/2018	0				
**UPE 706**	Faecal pool (x3)	**6/3/2018**	**1**	**beta**	**2**	MZ547522	BtCoV/Rousettus/LP/RSA/UPE706/2018
UPE 707	Faecal pool (x3)	6/3/2018	0				
**UPE 708**	Faecal pool (x3)	**6/3/2018**	**1**	**alpha**	**1**	MZ547474	BtCoV/Rousettus/LP/RSA/UPE708/2018
**UPE 709**	Faecal pool (x3)	**6/3/2018**	**1**	**beta**	**2**	MZ547523	BtCoV/Rousettus/LP/RSA/UPE709/2018
UPE 710	Faecal pool (x3)	6/3/2018	0				
**UPE 711**	Faecal pool (x3)	**6/3/2018**	**1**	**beta**	**2**	MZ547524	BtCoV/Rousettus/LP/RSA/UPE711/2018
UPE 712	Faecal pool (x3)	6/3/2018	0				
**UPE 713**	Faecal pool (x3)	**6/3/2018**	**1**	**beta**	**2**	MZ547525	BtCoV/Rousettus/LP/RSA/UPE713/2018

**UPE 714**	Faecal pool (x3)	**6/3/2018**	**1**	**alpha**	**1**	MZ547459	BtCoV/Rousettus/LP/RSA/UPE714/2018
UPE 715	Faecal pool (x3)	6/3/2018	0				
**UPE 716**	Faecal pool (x3)	**6/3/2018**	**1**	**beta**	**2**	MZ547526	BtCoV/Rousettus/LP/RSA/UPE716/2018
UPE 717	Faecal pool (x3)	6/3/2018	0				
**UPE 718**	Faecal pool (x3)	**6/3/2018**	**1**	**beta**	**2**	MZ547527	BtCoV/Rousettus/LP/RSA/UPE718/2018
**UPE 719**	Faecal pool (x3)	**6/3/2018**	**1**	**alpha**	**1**	MZ547475	BtCoV/Rousettus/LP/RSA/UPE719/2018
UPE 720	Faecal pool (x3)	6/3/2018	0				
UPE 721	Faecal pool (x3)	6/3/2018	0				
UPE 734	Faecal pool (x3)	11/4/2018	0				
**UPE 735**	Faecal pool (x3)	**11/4/2018**	**1**	**beta**	**2**	MZ547528	BtCoV/Rousettus/LP/RSA/UPE735/2018
UPE 736	Faecal pool (x3)	11/4/2018	0				
UPE 737	Faecal pool (x3)	11/4/2018	0				
UPE 738	Faecal pool (x3)	11/4/2018	0				
**UPE 739**	Faecal pool (x3)	**11/4/2018**	**1**	**beta**	**2**	MZ547529	BtCoV/Rousettus/LP/RSA/UPE739/2018
**UPE 740**	Faecal pool (x3)	**11/4/2018**	**1**	**beta**	**2**	MZ547530	BtCoV/Rousettus/LP/RSA/UPE740/2018
**UPE 741**	Faecal pool (x3)	**11/4/2018**	**1**	**beta**	**2**	MZ547531	BtCoV/Rousettus/LP/RSA/UPE741/2018
UPE 742	Faecal pool (x3)	11/4/2018	0				
UPE 743	Faecal pool (x3)	11/4/2018	0				
**UPE 744**	Faecal pool (x3)	**11/4/2018**	**1**	**beta**	**2**	MZ547532	BtCoV/Rousettus/LP/RSA/UPE744/2018
**UPE 745**	Faecal pool (x3)	**11/4/2018**	**1**	**beta**	**2**	MZ547533	BtCoV/Rousettus/LP/RSA/UPE745/2018
**UPE 746**	Faecal pool (x3)	**11/4/2018**	**1**	**beta**	**2**	MZ547534	BtCoV/Rousettus/LP/RSA/UPE746/2018
**UPE 747**	Faecal pool (x3)	**11/4/2018**	**1**	**beta**	**2**	MZ547535	BtCoV/Rousettus/LP/RSA/UPE747/2018
**UPE 748**	Faecal pool (x3)	**11/4/2018**	**1**	**beta**	**2**	MZ547536	BtCoV/Rousettus/LP/RSA/UPE748/2018
**UPE 749**	Faecal pool (x3)	**11/4/2018**	**1**	**beta**	**2**	MZ547537	BtCoV/Rousettus/LP/RSA/UPE749/2018
UPE 750	Faecal pool (x3)	11/4/2018	0				
**UPE 751**	Faecal pool (x3)	**11/4/2018**	**1**	**beta**	**2**	MZ547538	BtCoV/Rousettus/LP/RSA/UPE751/2018
UPE 752	Faecal pool (x3)	11/4/2018	0				
UPE 753	Faecal pool (x3)	11/4/2018	0				
**UPE 754**	Faecal pool (x3)	**11/4/2018**	**1**	**beta**	**2**	MZ547539	BtCoV/Rousettus/LP/RSA/UPE754/2018
**UPE 755**	Faecal pool (x3)	**11/4/2018**	**1**	**beta**	**2**	MZ547540	BtCoV/Rousettus/LP/RSA/UPE755/2018
UPE 756	Faecal pool (x3)	11/4/2018	0				
UPE 757	Faecal pool (x3)	11/4/2018	0				
**UPE 758**	Faecal pool (x3)	**11/4/2018**	**1**	**beta**	**2**	MZ547541	BtCoV/Rousettus/LP/RSA/UPE758/2018
**UPE 759**	Faecal pool (x3)	**11/4/2018**	**1**	**beta**	**2**	MZ547542	BtCoV/Rousettus/LP/RSA/UPE759/2018
UPE 770	Faecal pool (x3)	9/5/2018	0				
UPE 771	Faecal pool (x3)	9/5/2018	0				
UPE 772	Faecal pool (x3)	9/5/2018	0				
UPE 773	Faecal pool (x3)	9/5/2018	0				
UPE 774	Faecal pool (x3)	9/5/2018	0				
UPE 775	Faecal pool (x3)	9/5/2018	0				
**UPE 776**	Faecal pool (x3)	**9/5/2018**	**1**	**beta**	**2**	MZ547543	BtCoV/Rousettus/LP/RSA/UPE776/2018
UPE 777	Faecal pool (x3)	9/5/2018	0				
UPE 778	Faecal pool (x3)	9/5/2018	0				
**UPE 779**	Faecal pool (x3)	**9/5/2018**	**1**	**beta**	**2**	MZ547544	BtCoV/Rousettus/LP/RSA/UPE779/2018
UPE 780	Faecal pool (x3)	9/5/2018	0				
**UPE 781**	Faecal pool (x3)	**9/5/2018**	**1**	**beta**	**2**	MZ547545	BtCoV/Rousettus/LP/RSA/UPE781/2018
**UPE 782**	Faecal pool (x3)	**9/5/2018**	**1**	**beta**	**2**	MZ547546	BtCoV/Rousettus/LP/RSA/UPE782/2018
UPE 783	Faecal pool (x3)	9/5/2018	0				
UPE 784	Faecal pool (x3)	9/5/2018	0				
UPE 785	Faecal pool (x3)	9/5/2018	0				
UPE 791	Faecal pool (x3)	6/6/2018	0				
UPE 792	Faecal pool (x3)	6/6/2018	0				
UPE 793	Faecal pool (x3)	6/6/2018	0				
UPE 794	Faecal pool (x3)	6/6/2018	0				
UPE 795	Faecal pool (x3)	6/6/2018	0				
**UPE 796**	Faecal pool (x3)	**6/6/2018**	**1**	**beta**	**2**	MZ547547	BtCoV/Rousettus/LP/RSA/UPE796/2018
**UPE 797**	Faecal pool (x3)	**6/6/2018**	**1**	**beta**	**2**	MZ547548	BtCoV/Rousettus/LP/RSA/UPE797/2018
UPE 811	Faecal pool (x3)	6/6/2018	0				
UPE 818	Faecal pool (x3)	3/7/2018	0				
**UPE 819**	Faecal pool (x3)	**3/7/2018**	**1**	**beta**	**3**	MZ547497	BtCoV/Rousettus/LP/RSA/UPE819/2018
UPE 820	Faecal pool (x3)	3/7/2018	0				
UPE 821	Faecal pool (x3)	3/7/2018	0				
UPE 822	Faecal pool (x3)	3/7/2018	0				
**UPE 823**	Faecal pool (x3)	**3/7/2018**	**1**	**beta**	**3**	MZ547498	BtCoV/Rousettus/LP/RSA/UPE823/2018
UPE 824	Faecal pool (x3)	3/7/2018	0				
UPE 825	Faecal pool (x3)	3/7/2018	0				
UPE 830	Faecal pool (x3)	3/7/2018	0				
UPE 831	Faecal pool (x3)	1/8/2018	0				
UPE 832	Faecal pool (x3)	1/8/2018	0				
UPE 833	Faecal pool (x3)	1/8/2018	0				
UPE 834	Faecal pool (x3)	1/8/2018	0				
UPE 835	Faecal pool (x3)	1/8/2018	0				
UPE 836	Faecal pool (x3)	1/8/2018	0				
UPE 837	Faecal pool (x3)	1/8/2018	0				
UPE 838	Faecal pool (x3)	1/8/2018	0				
UPE 904	Faecal pool (x3)	5/9/2018	0				
UPE 905	Faecal pool (x3)	5/9/2018	0				
UPE 906	Faecal pool (x3)	5/9/2018	0				
UPE 907	Faecal pool (x3)	5/9/2018	0				

UPE 908	Faecal pool (x3)	5/9/2018	0				
UPE 909	Faecal pool (x3)	5/9/2018	0				
UPE 910	Faecal pool (x3)	5/9/2018	0				
UPE 911	Faecal pool (x3)	5/9/2018	0				
UPE 912	Faecal pool (x3)	5/9/2018	0				
UPE 913	Faecal pool (x3)	5/9/2018	0				
UPE 914	Faecal pool (x3)	5/9/2018	0				
UPE 915	Faecal pool (x3)	5/9/2018	0				
UPE 916	Faecal pool (x3)	5/9/2018	0				
UPE 917	Faecal pool (x3)	5/9/2018	0				
UPE 918	Faecal pool (x3)	5/9/2018	0				
UPE 919	Faecal pool (x3)	5/9/2018	0				
UPE 920	Faecal pool (x3)	5/9/2018	0				
UPE 921	Faecal pool (x3)	5/9/2018	0				
UPE 922	Faecal pool (x3)	5/9/2018	0				
UPE 923	Faecal pool (x3)	5/9/2018	0				
UPE 924	Faecal pool (x3)	5/9/2018	0				
UPE 925	Faecal pool (x3)	5/9/2018	0				
UPE 926	Faecal pool (x3)	5/9/2018	0				
UPE 927	Faecal pool (x3)	5/9/2018	0				
UPE 928	Faecal pool (x3)	5/9/2018	0				
UPE 929	Faecal pool (x3)	5/9/2018	0				
UPE 930	Faecal pool (x3)	5/9/2018	0				
UPE 931	Faecal pool (x3)	5/9/2018	0				
UPE 932	Faecal pool (x3)	5/9/2018	0				
UPE 933	Faecal pool (x3)	5/9/2018	0				
UPE 995	Faecal pool (x3)	2/10/2018	0				
UPE 996	Faecal pool (x3)	2/10/2018	0				
**UPE 997**	Faecal pool (x3)	**2/10/2018**	**1**	**beta**	**3**	MZ547499	BtCoV/Rousettus/LP/RSA/UPE997/2018
**UPE 998**	Faecal pool (x3)	**2/10/2018**	**1**	**beta**	**3**	MZ547500	BtCoV/Rousettus/LP/RSA/UPE998/2018
UPE 999	Faecal pool (x3)	2/10/2018	0				
**UPE 1000**	Faecal pool (x3)	**2/10/2018**	**1**	**beta**	**3**	MZ547501	BtCoV/Rousettus/LP/RSA/UPE1000/2018
UPE 1001	Faecal pool (x3)	2/10/2018	0				
UPE 1002	Faecal pool (x3)	2/10/2018	0				
UPE 1003	Faecal pool (x3)	2/10/2018	0				
UPE 1004	Faecal pool (x3)	2/10/2018	0				
UPE 1005	Faecal pool (x3)	2/10/2018	0				
UPE 1006	Faecal pool (x3)	2/10/2018	0				
UPE 1007	Faecal pool (x3)	2/10/2018	0				
UPE 1008	Faecal pool (x3)	2/10/2018	0				
UPE 1009	Faecal pool (x3)	2/10/2018	0				
UPE 1010	Faecal pool (x3)	2/10/2018	0				
UPE 1011	Faecal pool (x3)	2/10/2018	0				
UPE 1012	Faecal pool (x3)	2/10/2018	0				
UPE 1013	Faecal pool (x3)	2/10/2018	0				
UPE 1014	Faecal pool (x3)	2/10/2018	0				
UPE 1015	Faecal pool (x3)	2/10/2018	0				
UPE 1016	Faecal pool (x3)	2/10/2018	0				
UPE 1017	Faecal pool (x3)	2/10/2018	0				
**UPE 1018**	Faecal pool (x3)	**2/10/2018**	**1**	**beta**	**2**	MZ547571	BtCoV/Rousettus/LP/RSA/UPE1018/2018
**UPE 1019**	Faecal pool (x3)	**2/10/2018**	**1**	**beta**	**2**	MZ547550	BtCoV/Rousettus/LP/RSA/UPE1019/2018
UPE 1020	Faecal pool (x3)	2/10/2018	0				
UPE 1021	Faecal pool (x3)	2/10/2018	0				
UPE 1022	Faecal pool (x3)	2/10/2018	0				
UPE 1023	Faecal pool (x3)	2/10/2018	0				
UPE 1024	Faecal pool (x3)	2/10/2018	0				
UPE 1025	Faecal pool (x3)	2/10/2018	0				
UPE 1026	Faecal pool (x3)	2/10/2018	0				
UPE 1027	Faecal pool (x3)	2/10/2018	0				
UPE 1028	Faecal pool (x3)	2/10/2018	0				
UPE 1029	Faecal pool (x3)	2/10/2018	0				
UPE 1030	Faecal pool (x3)	2/10/2018	0				
UPE 1094	Faecal pool (x3)	7/11/2018	0				
UPE 1095	Faecal pool (x3)	7/11/2018	0				
UPE 1096	Faecal pool (x3)	7/11/2018	0				
UPE 1097	Faecal pool (x3)	7/11/2018	0				
UPE 1098	Faecal pool (x3)	7/11/2018	0				
UPE 1099	Faecal pool (x3)	7/11/2018	0				
UPE 1100	Faecal pool (x3)	7/11/2018	0				
UPE 1101	Faecal pool (x3)	7/11/2018	0				
UPE 1102	Faecal pool (x3)	7/11/2018	0				
UPE 1103	Faecal pool (x3)	7/11/2018	0				
UPE 1104	Faecal pool (x3)	7/11/2018	0				
UPE 1105	Faecal pool (x3)	7/11/2018	0				
UPE 1106	Faecal pool (x3)	7/11/2018	0				
UPE 1107	Faecal pool (x3)	7/11/2018	0				
UPE 1108	Faecal pool (x3)	7/11/2018	0				
UPE 1109	Faecal pool (x3)	7/11/2018	0				
UPE 1110	Faecal pool (x3)	7/11/2018	0				

UPE 1111	Faecal pool (x3)	7/11/2018	0				
UPE 1112	Faecal pool (x3)	7/11/2018	0				
UPE 1113	Faecal pool (x3)	7/11/2018	0				
UPE 1114	Faecal pool (x3)	7/11/2018	0				
UPE 1115	Faecal pool (x3)	7/11/2018	0				
UPE 1116	Faecal pool (x3)	7/11/2018	0				
UPE 1117	Faecal pool (x3)	7/11/2018	0				
UPE 1118	Faecal pool (x3)	7/11/2018	0				
UPE 1119	Faecal pool (x3)	7/11/2018	0				
UPE 1120	Faecal pool (x3)	7/11/2018	0				
**UPE 1121**	Faecal pool (x3)	**7/11/2018**	**1**	**beta**	**2**	MZ547549	BtCoV/Rousettus/LP/RSA/UPE1121/2018
UPE 1122	Faecal pool (x3)	7/11/2018	0				
UPE 1123	Faecal pool (x3)	7/11/2018	0				
UPE 1124	Faecal pool (x3)	7/11/2018	0				
UPE 1125	Faecal pool (x3)	7/11/2018	0				
UPE 1126	Faecal pool (x3)	7/11/2018	0				
UPE 1127	Faecal pool (x3)	7/11/2018	0				
UPE 1128	Faecal pool (x3)	7/11/2018	0				
UPE 1129	Faecal pool (x3)	7/11/2018	0				
UPE 1130	Faecal pool (x3)	7/11/2018	0				
UPE 1131	Faecal pool (x3)	7/11/2018	0				
UPE 1132	Faecal pool (x3)	7/11/2018	0				
UPE 1133	Faecal pool (x3)	7/11/2018	0				
UPE 1134	Faecal pool (x3)	7/11/2018	0				
UPE 1287	Faecal pool (x3)	4/12/2018	0				
UPE 1288	Faecal pool (x3)	4/12/2018	0				
UPE 1289	Faecal pool (x3)	4/12/2018	0				
UPE 1290	Faecal pool (x3)	4/12/2018	0				
UPE 1291	Faecal pool (x3)	4/12/2018	0				
UPE 1292	Faecal pool (x3)	4/12/2018	0				
UPE 1293	Faecal pool (x3)	4/12/2018	0				
UPE 1294	Faecal pool (x3)	4/12/2018	0				
**UPE 1295**	Faecal pool (x3)	**4/12/2018**	**1**	**beta**	**2**	MZ547551	BtCoV/Rousettus/LP/RSA/UPE1295/2018
UPE 1296	Faecal pool (x3)	4/12/2018	0				
UPE 1297	Faecal pool (x3)	4/12/2018	0				
UPE 1298	Faecal pool (x3)	4/12/2018	0				
UPE 1299	Faecal pool (x3)	4/12/2018	0				
UPE 1300	Faecal pool (x3)	4/12/2018	0				
UPE 1301	Faecal pool (x3)	4/12/2018	0				
UPE 1302	Faecal pool (x3)	4/12/2018	0				
UPE 1303	Faecal pool (x3)	4/12/2018	0				
UPE 1304	Faecal pool (x3)	4/12/2018	0				
**UPE 1305**	Faecal pool (x3)	**4/12/2018**	**1**	**alpha**	**1**	MZ547476	BtCoV/Rousettus/LP/RSA/UPE1305/2018
**UPE 1306**	Faecal pool (x3)	**4/12/2018**	**1**	**alpha**	**1**	MZ547477	BtCoV/Rousettus/LP/RSA/UPE1306/2018
**UPE 1307**	Faecal pool (x3)	**4/12/2018**	**1**	**alpha**	**1**	MZ547478	BtCoV/Rousettus/LP/RSA/UPE1307/2018
UPE 1308	Faecal pool (x3)	4/12/2018	0				
UPE 1309	Faecal pool (x3)	4/12/2018	0				
UPE 1310	Faecal pool (x3)	4/12/2018	0				
**UPE 1311**	Faecal pool (x3)	**4/12/2018**	**1**	**alpha**	**1**	MZ547479	BtCoV/Rousettus/LP/RSA/UPE1311/2018
UPE 1313	Faecal pool (x3)	4/12/2018	0				
UPE 1314	Faecal pool (x3)	4/12/2018	0				
UPE 1315	Faecal pool (x3)	4/12/2018	0				
UPE 1316	Faecal pool (x3)	4/12/2018	0				
UPE 1317	Faecal pool (x3)	4/12/2018	0				
UPE 1318	Faecal pool (x3)	4/12/2018	0				
UPE 1319	Faecal pool (x3)	4/12/2018	0				
UPE 1320	Faecal pool (x3)	4/12/2018	0				
UPE 1321	Faecal pool (x3)	4/12/2018	0				
UPE 1322	Faecal pool (x3)	4/12/2018	0				
UPE 1323	Faecal pool (x3)	4/12/2018	0				
UPE 1324	Faecal pool (x3)	4/12/2018	0				
UPE 1325	Faecal pool (x3)	4/12/2018	0				
UPE 1326	Faecal pool (x3)	4/12/2018	0				
UPE 1446	Faecal pool (x3)	15/1/2019	0				
UPE 1447	Faecal pool (x3)	15/1/2019	0				
UPE 1448	Faecal pool (x3)	15/1/2019	0				
UPE 1449	Faecal pool (x3)	15/1/2019	0				
UPE 1450	Faecal pool (x3)	15/1/2019	0				
UPE 1451	Faecal pool (x3)	15/1/2019	0				
UPE 1452	Faecal pool (x3)	15/1/2019	0				
UPE 1453	Faecal pool (x3)	15/1/2019	0				
UPE 1454	Faecal pool (x3)	15/1/2019	0				
**UPE 1455**	Faecal pool (x3)	**15/1/2019**	**1**	**alpha**	**1**	MZ547480	BtCoV/Rousettus/LP/RSA/UPE1455/2019
UPE 1456	Faecal pool (x3)	15/1/2019	0				
UPE 1457	Faecal pool (x3)	15/1/2019	0				
UPE 1458	Faecal pool (x3)	15/1/2019	0				
UPE 1459	Faecal pool (x3)	15/1/2019	0				
**UPE 1460**	Faecal pool (x3)	**15/1/2019**	**1**	**alpha**	**1**	MZ547481	BtCoV/Rousettus/LP/RSA/UPE1460/2019
UPE 1461	Faecal pool (x3)	15/1/2019	0				

**UPE 1462**	Faecal pool (x3)	**15/1/2019**	**1**	**alpha**	**1**	MZ547482	BtCoV/Rousettus/LP/RSA/UPE1462/2019
UPE 1463	Faecal pool (x3)	15/1/2019	0				
UPE 1464	Faecal pool (x3)	15/1/2019	0				
UPE 1465	Faecal pool (x3)	15/1/2019	0				
UPE 1466	Faecal pool (x3)	15/1/2019	0				
**UPE 1467**	Faecal pool (x3)	**15/1/2019**	**1**	**alpha**	**1**	MZ547483	BtCoV/Rousettus/LP/RSA/UPE1467/2019
UPE 1468	Faecal pool (x3)	15/1/2019	0				
UPE 1469	Faecal pool (x3)	15/1/2019	0				
**UPE 1470**	Faecal pool (x3)	**15/1/2019**	**1**	**beta**	**2**	MZ547552	BtCoV/Rousettus/LP/RSA/UPE1470/2019
**UPE 1471**	Faecal pool (x3)	**15/1/2019**	**1**	**alpha**	**1**	MZ547484	BtCoV/Rousettus/LP/RSA/UPE1471/2019
UPE 1472	Faecal pool (x3)	15/1/2019	0				
UPE 1473	Faecal pool (x3)	15/1/2019	0				
UPE 1474	Faecal pool (x3)	15/1/2019	0				
UPE 1475	Faecal pool (x3)	15/1/2019	0				
UPE 1476	Faecal pool (x3)	15/1/2019	0				
UPE 1477	Faecal pool (x3)	15/1/2019	0				
UPE 1478	Faecal pool (x3)	15/1/2019	0				
**UPE 1479**	Faecal pool (x3)	**15/1/2019**	**1**	**beta**	**2**	MZ547553	BtCoV/Rousettus/LP/RSA/UPE1479/2019
UPE 1480	Faecal pool (x3)	15/1/2019	0				
UPE 1481	Faecal pool (x3)	15/1/2019	0				
UPE 1482	Faecal pool (x3)	15/1/2019	0				
UPE 1483	Faecal pool (x3)	15/1/2019	0				
UPE 1484	Faecal pool (x3)	15/1/2019	0				
UPE 1485	Faecal pool (x3)	15/1/2019	0				
UPE 1486	Faecal pool (x3)	15/1/2019	0				
**UPE 1547**	Faecal pool (x3)	**12/2/2019**	**1**	**alpha**	**1**	MZ547485	BtCoV/Rousettus/LP/RSA/UPE1547/2019
UPE 1548	Faecal pool (x3)	12/2/2019	0				
UPE 1549	Faecal pool (x3)	12/2/2019	0				
UPE 1550	Faecal pool (x3)	12/2/2019	0				
UPE 1551	Faecal pool (x3)	12/2/2019	0				
UPE 1552	Faecal pool (x3)	12/2/2019	0				
**UPE 1553**	Faecal pool (x3)	**12/2/2019**	**1**	**beta**	**2**	MZ547554	BtCoV/Rousettus/LP/RSA/UPE1553/2019
**UPE 1554**	Faecal pool (x3)	**12/2/2019**	**1**	**alpha**	**1**	MZ547486	BtCoV/Rousettus/LP/RSA/UPE1554/2019
UPE 1555	Faecal pool (x3)	12/2/2019	0				
UPE 1556	Faecal pool (x3)	12/2/2019	0				
UPE 1557	Faecal pool (x3)	12/2/2019	0				
UPE 1558	Faecal pool (x3)	12/2/2019	0				
**UPE 1559**	Faecal pool (x3)	**12/2/2019**	**1**	**alpha**	**1**	MZ547487	BtCoV/Rousettus/LP/RSA/UPE1559/2019
UPE 1560	Faecal pool (x3)	12/2/2019	0				
UPE 1561	Faecal pool (x3)	12/2/2019	0				
**UPE 1562**	Faecal pool (x3)	**12/2/2019**	**1**	**beta**	**2**	MZ547555	BtCoV/Rousettus/LP/RSA/UPE1562/2019
**UPE 1563**	Faecal pool (x3)	**12/2/2019**	**1**	**beta**	**2**	MZ547567	BtCoV/Rousettus/LP/RSA/UPE1563/2019
UPE 1564	Faecal pool (x3)	12/2/2019	0				
UPE 1565	Faecal pool (x3)	12/2/2019	0				
UPE 1566	Faecal pool (x3)	12/2/2019	0				
UPE 1567	Faecal pool (x3)	12/2/2019	0				
UPE 1568	Faecal pool (x3)	12/2/2019	0				
**UPE 1569**	Faecal pool (x3)	**12/2/2019**	**1**	**beta**	**2**	MZ547556	BtCoV/Rousettus/LP/RSA/UPE1569/2019
UPE 1570	Faecal pool (x3)	12/2/2019	0				
**UPE 1571**	Faecal pool (x3)	**12/2/2019**	**1**	**beta**	**2**	MZ547557	BtCoV/Rousettus/LP/RSA/UPE1571/2019
UPE 1572	Faecal pool (x3)	12/2/2019	0				
UPE 1573	Faecal pool (x3)	12/2/2019	0				
**UPE 1574**	Faecal pool (x3)	**12/2/2019**	**1**	**alpha**	**1**	MZ547488	BtCoV/Rousettus/LP/RSA/UPE1574/2019
UPE 1575	Faecal pool (x3)	12/2/2019	0				
UPE 1576	Faecal pool (x3)	12/2/2019	0				
**UPE 1577**	Faecal pool (x3)	**12/2/2019**	**1**	**beta**	**2**	MZ547572	BtCoV/Rousettus/LP/RSA/UPE1577/2019
**UPE 1578**	Faecal pool (x3)	**12/2/2019**	**1**	**beta**	**2**	MZ547558	BtCoV/Rousettus/LP/RSA/UPE1578/2019
UPE 1579	Faecal pool (x3)	12/2/2019	0				
UPE 1580	Faecal pool (x3)	12/2/2019	0				
**UPE 1581**	Faecal pool (x3)	**12/2/2019**	**1**	**beta**	**2**	MZ547559	BtCoV/Rousettus/LP/RSA/UPE1581/2019
UPE 1582	Faecal pool (x3)	12/2/2019	0				
UPE 1583	Faecal pool (x3)	12/2/2019	0				
UPE 1584	Faecal pool (x3)	12/2/2019	0				
UPE 1585	Faecal pool (x3)	12/2/2019	0				
UPE 1586	Faecal pool (x3)	12/2/2019	0				
UPE 1587	Faecal pool (x3)	12/2/2019	0				
UPE 1588	Faecal pool (x3)	12/2/2019	0				
**UPE 1589**	Faecal pool (x3)	**12/2/2019**	**1**	**beta**	**2**	MZ547560	BtCoV/Rousettus/LP/RSA/UPE1589/2019
**UPE 1590**	Faecal pool (x3)	**12/2/2019**	**1**	**beta**	**2**	MZ547561	BtCoV/Rousettus/LP/RSA/UPE1590/2019
**UPE 1591**	Faecal pool (x3)	**12/2/2019**	**1**	**alpha**	**1**	MZ547489	BtCoV/Rousettus/LP/RSA/UPE1591/2019
UPE 1592	Faecal pool (x3)	12/2/2019	0				
UPE 1593	Faecal pool (x3)	12/2/2019	0				
UPE 1594	Faecal pool (x3)	12/2/2019	0				
UPE 1663	Faecal pool (x3)	5/3/2019	0				
UPE 1664	Faecal pool (x3)	5/3/2019	0				
UPE 1665	Faecal pool (x3)	5/3/2019	0				
UPE 1666	Faecal pool (x3)	5/3/2019	0				
UPE 1667	Faecal pool (x3)	5/3/2019	0				
UPE 1668	Faecal pool (x3)	5/3/2019	0				

UPE 1669	Faecal pool (x3)	5/3/2019	0				
**UPE 1670**	Faecal pool (x3)	**5/3/2019**	**1**	**alpha**	**1**	MZ547490	BtCoV/Rousettus/LP/RSA/UPE1670/2019
UPE 1671	Faecal pool (x3)	5/3/2019	0				
**UPE 1672**	Faecal pool (x3)	**5/3/2019**	**1**	**alpha**	**1**	MZ547491	BtCoV/Rousettus/LP/RSA/UPE1672/2019
UPE 1673	Faecal pool (x3)	5/3/2019	0				
UPE 1674	Faecal pool (x3)	5/3/2019	0				
UPE 1675	Faecal pool (x3)	5/3/2019	0				
UPE 1676	Faecal pool (x3)	5/3/2019	0				
UPE 1677	Faecal pool (x3)	5/3/2019	0				
UPE 1678	Faecal pool (x3)	5/3/2019	0				
UPE 1679	Faecal pool (x3)	5/3/2019	0				
UPE 1680	Faecal pool (x3)	5/3/2019	0				
UPE 1681	Faecal pool (x3)	5/3/2019	0				
**UPE 1682**	Faecal pool (x3)	**5/3/2019**	**1**	**beta**	**2**	MZ547566	BtCoV/Rousettus/LP/RSA/UPE1682/2019
UPE 1683	Faecal pool (x3)	5/3/2019	0				
UPE 1684	Faecal pool (x3)	5/3/2019	0				
UPE 1685	Faecal pool (x3)	5/3/2019	0				
UPE 1686	Faecal pool (x3)	5/3/2019	0				
UPE 1687	Faecal pool (x3)	5/3/2019	0				
UPE 1688	Faecal pool (x3)	5/3/2019	0				
UPE 1689	Faecal pool (x3)	5/3/2019	0				
UPE 1690	Faecal pool (x3)	5/3/2019	0				
UPE 1725	Faecal pool (x3)	3/4/2019	0				
UPE 1726	Faecal pool (x3)	3/4/2019	0				
UPE 1727	Faecal pool (x3)	3/4/2019	0				
UPE 1728	Faecal pool (x3)	3/4/2019	0				
UPE 1729	Faecal pool (x3)	3/4/2019	0				
UPE 1730	Faecal pool (x3)	3/4/2019	0				
UPE 1731	Faecal pool (x3)	3/4/2019	0				
UPE 1732	Faecal pool (x3)	3/4/2019	0				
UPE 1733	Faecal pool (x3)	3/4/2019	0				
UPE 1734	Faecal pool (x3)	3/4/2019	0				
UPE 1735	Faecal pool (x3)	3/4/2019	0				
UPE 1736	Faecal pool (x3)	3/4/2019	0				
UPE 1737	Faecal pool (x3)	3/4/2019	0				
UPE 1738	Faecal pool (x3)	3/4/2019	0				
**UPE 1739**	Faecal pool (x3)	**3/4/2019**	**1**	**beta**	**2**	MZ547565	BtCoV/Rousettus/LP/RSA/UPE1739/2019
UPE 1740	Faecal pool (x3)	3/4/2019	0				
UPE 1741	Faecal pool (x3)	3/4/2019	0				
UPE 1742	Faecal pool (x3)	3/4/2019	0				
UPE 1743	Faecal pool (x3)	3/4/2019	0				
UPE 1744	Faecal pool (x3)	3/4/2019	0				
UPE 1745	Faecal pool (x3)	3/4/2019	0				
UPE 1746	Faecal pool (x3)	3/4/2019	0				
UPE 1747	Faecal pool (x3)	3/4/2019	0				
UPE 1748	Faecal pool (x3)	3/4/2019	0				
UPE 1749	Faecal pool (x3)	3/4/2019	0				
UPE 1750	Faecal pool (x3)	3/4/2019	0				
UPE 1751	Faecal pool (x3)	3/4/2019	0				
UPE 1752	Faecal pool (x3)	3/4/2019	0				
UPE 1753	Faecal pool (x3)	3/4/2019	0				
UPE 1754	Faecal pool (x3)	3/4/2019	0				
UPE 1755	Faecal pool (x3)	3/4/2019	0				
UPE 1756	Faecal pool (x3)	3/4/2019	0				
UPE 1757	Faecal pool (x3)	3/4/2019	0				
UPE 1758	Faecal pool (x3)	3/4/2019	0				
UPE 1759	Faecal pool (x3)	3/4/2019	0				
UPE 1760	Faecal pool (x3)	3/4/2019	0				
UPE 1761	Faecal pool (x3)	3/4/2019	0				
UPE 1762	Faecal pool (x3)	3/4/2019	0				
UPE 1763	Faecal pool (x3)	3/4/2019	0				
UPE 1764	Faecal pool (x3)	3/4/2019	0				
UPE 1765	Faecal pool (x3)	3/4/2019	0				
UPE 1766	Faecal pool (x3)	3/4/2019	0				
UPE 1767	Faecal pool (x3)	3/4/2019	0				
UPE 1833	Faecal pool (x3)	15/5/2019	0				
UPE 1834	Faecal pool (x3)	15/5/2019	0				
UPE 1835	Faecal pool (x3)	15/5/2019	0				
UPE 1836	Faecal pool (x3)	15/5/2019	0				
UPE 1837	Faecal pool (x3)	15/5/2019	0				
UPE 1838	Faecal pool (x3)	15/5/2019	0				
UPE 1839	Faecal pool (x3)	15/5/2019	0				
UPE 1840	Faecal pool (x3)	15/5/2019	0				
UPE 1841	Faecal pool (x3)	15/5/2019	0				
UPE 1842	Faecal pool (x3)	15/5/2019	0				
UPE 1843	Faecal pool (x3)	15/5/2019	0				
UPE 1844	Faecal pool (x3)	15/5/2019	0				
UPE 1845	Faecal pool (x3)	15/5/2019	0				
UPE 1846	Faecal pool (x3)	15/5/2019	0				

UPE 1847	Faecal pool (x3)	15/5/2019	0				
UPE 1848	Faecal pool (x3)	15/5/2019	0				
UPE 1849	Faecal pool (x3)	15/5/2019	0				
UPE 1850	Faecal pool (x3)	15/5/2019	0				
UPE 1851	Faecal pool (x3)	15/5/2019	0				
**UPE 1852**	Faecal pool (x3)	**15/5/2019**	**1**	**beta**	**2**	MZ547564	BtCoV/Rousettus/LP/RSA/UPE1852/2019
UPE 1853	Faecal pool (x3)	15/5/2019	0				
**UPE 1854**	Faecal pool (x3)	**15/5/2019**	**1**	**beta**	**2**	MZ547563	BtCoV/Rousettus/LP/RSA/UPE1854/2019
UPE 1855	Faecal pool (x3)	15/5/2019	0				
UPE 1856	Faecal pool (x3)	15/5/2019	0				
UPE 1857	Faecal pool (x3)	15/5/2019	0				
**UPE 1858**	Faecal pool (x3)	**15/5/2019**	**1**	**beta**	**2**	MZ547562	BtCoV/Rousettus/LP/RSA/UPE1858/2019
UPE 1859	Faecal pool (x3)	15/5/2019	0				

Dataset 2: Sample information of individual bat samples

UP	Tatoo	Sample type	Date collected	CoV detected	Genus	Clade	Accession number	Strain	Recapture bats infection status	Gender	Age	FA (mm)	Mass (g)	FMI (kg/m2)	Reproductive status	Reproductive condition	Teat assessment
UP 8375	SMC 540	Rectal swab	7/11/2017	0						M	A	91.5	145	17.319	Active	Scrotal	Not applicable
UP 8376	SMC 541	Rectal swab	7/11/2017	0						F	A	92.2	74	8.705	Active	Pregnant	Not recorded
UP 8377	SMC 542	Rectal swab	7/11/2017	0						F	A	88.8	142	18.008	Active	Pregnant	Non-sclerotized teats
UP 8378	SMC 543	Rectal swab	7/11/2017	0						F	SA	88.7	109	13.854	Not-active	Not pregnant	Non-sclerotized teats
UP 8379	SMC 544	Rectal swab	7/11/2017	0						F	A	94.8	137	15.244	Active	Pregnant	Not recorded
UP 8380	SMC 545	Rectal swab	7/11/2017	0						F	A	91.3	135	16.195	Active	Pregnant	Not recorded
UP 8381	SMC 546	Rectal swab	7/11/2017	0						M	A	90.9	110	13.313	Active	Not scrotal	Not applicable
UP 8382	SMC 547	Rectal swab	7/11/2017	0						F	A	97.6	175	18.371	Active	Pregnant	Not recorded
UP 8383	SMC 548	Rectal swab	7/11/2017	0						F	A	89.9	126	15.590	Active	Pregnant	Not recorded
UP 8385	SMC 550	Rectal swab	7/11/2017	0						F	A	88.6	151	19.236	Active	Pregnant	Non-sclerotized teats
UP 8387	SMC 552	Rectal swab	7/11/2017	1	Beta	2	MZ547578	BtCoV/Rousettus/LP/RSA/UP8387/2017		M	SA	88.8	112	14.203	Not-active	Not scrotal	Not applicable
UP 8388	SMC 553	Rectal swab	7/11/2017	0						F	SA	88.6	127	16.178	Not-active	Not pregnant	Non-sclerotized teats
UP 8558	SMC 575	Rectal swab	5/12/2017	0						F	A	92.1	132	15.562	Active	Lactating	Sclerotized teats
UP 8559	SMC 576	Rectal swab	5/12/2017	0						F	A	91.8	161	19.105	Active	Lactating	Sclerotized teats
UP 8560	SMC 577	Rectal swab	5/12/2017	0						M	A	98.7	153	15.706	Active	Not scrotal	Not applicable
UP 8561	SMC 578	Rectal swab	5/12/2017	0						F	A	97.2	179	18.946	Active	Lactating	Sclerotized teats
UP 8562	SMC 579	Rectal swab	5/12/2017	0						F	A	93.4	158	18.112	Active	Lactating	Sclerotized teats
UP 8563	SMC 580	Rectal swab	5/12/2017	0						F	A	93	176	20.349	Active	Lactating	Sclerotized teats
UP 8564	SMC 581	Rectal swab	5/12/2017	0						M	SA	86.5	97	12.964	Not-active	Not scrotal	Not applicable
UP 8565	SMC 582	Rectal swab	5/12/2017	0						F	A	96	127	13.780	Active	Lactating	Sclerotized teats
UP 8566	SMC 583	Rectal swab	5/12/2017	0						F	A	92.7	144	16.757	Active	Lactating	Sclerotized teats
UP 8567	SMC 584	Rectal swab	5/12/2017	0						F	A	92.3	156	18.311	Active	Lactating	Sclerotized teats
UP 8568	SMC 585	Rectal swab	5/12/2017	0						M	A	94.3	146	16.418	Active	Scrotal	Not applicable
UP 8569	SMC 586	Rectal swab	5/12/2017	0						F	A	95.3	121	13.323	Active	Lactating	Sclerotized teats
UP 8570	SMC 587	Rectal swab	5/12/2017	0						F	A	93.7	147	16.743	Active	Lactating	Sclerotized teats
UP 8685	SMC 622	Rectal swab	7/2/2018	0						M	SA	82.2	69	10.212	Not-active	Not scrotal	Not applicable
UP 8686	SMC 623	Rectal swab	7/2/2018	0						F	SA	82	66	9.816	Not-active	Not pregnant	Non-sclerotized teats
UP 8687	SMC 624	Rectal swab	7/2/2018	1	Beta	2	MZ547579	BtCoV/Rousettus/LP/RSA/UP8687/2018		M	SA	84	91	12.897	Not-active	Not scrotal	Not applicable
UP 8688	SMC 625	Rectal swab	7/2/2018	0						M	SA	80	81	12.656	Not-active	Not scrotal	Not applicable
UP 8689	SMC 626	Rectal swab	7/2/2018	0						M	A	99.1	71	7.230	Active	Not scrotal	Not applicable
UP 8690	SMC 627	Rectal swab	7/2/2018	0						F	SA	80.4	66	10.210	Not-active	Not pregnant	Non-sclerotized teats
UP 8691	SMC 628	Rectal swab	7/2/2018	0						F	SA	81.6	67	10.062	Not-active	Not pregnant	Non-sclerotized teats
UP 8692	SMC 629	Rectal swab	7/2/2018	0						M	SA	82.9	74	10.768	Not-active	Not scrotal	Not applicable
UP 8693	SMC 630	Rectal swab	7/2/2018	0						M	SA	86	72	9.735	Not-active	Not scrotal	Not applicable
UP 8695	SMC 632	Rectal swab	7/2/2018	0						F	SA	81.7	65	9.738	Not-active	Not pregnant	Non-sclerotized teats
UP 8696	SMC 633	Rectal swab	7/2/2018	1	Alpha	1	MZ547460	BtCoV/Rousettus/LP/RSA/UP8696/2018		M	A	95.2	135	14.896	Active	Scrotal	Not applicable
UP 8697	SMC 634	Rectal swab	7/2/2018	0						M	SA	83.6	81	11.590	Not-active	Not scrotal	Not applicable
UP 8698	SMC 635	Rectal swab	7/2/2018	0						F	A	93.2	110	12.664	Active	Not pregnant	Not recorded
UP 8699	SMC 636	Rectal swab	7/2/2018	0						F	SA	80.6	79	12.161	Not-active	Not pregnant	Non-sclerotized teats
UP 8700	SMC 637	Rectal swab	7/2/2018	0						F	A	94.2	118	13.298	Active	Not pregnant	Not recorded
UP 8701	SMC 638	Rectal swab	7/2/2018	1	Alpha	1	MZ547451	BtCoV/Rousettus/LP/RSA/UP8701/2018		F	A	91.4	98	11.731	Active	Not pregnant	Not recorded
UP 8702	SMC 639	Rectal swab	7/2/2018	1	Beta	2	MZ547575	BtCoV/Rousettus/LP/RSA/UP8702/2018		F	SA	76.2	63	10.850	Not-active	Not pregnant	Non-sclerotized teats
UP 8703	SMC 640	Rectal swab	7/2/2018	0						F	SA	81.5	85	12.797	Not-active	Not pregnant	Non-sclerotized teats
UP 8704	SMC 641	Rectal swab	7/2/2018	0						M	SA	82.8	84	12.252	Not-active	Not scrotal	Not applicable
UP 8705	SMC 642	Rectal swab	7/2/2018	1	Beta	2	MZ547580	BtCoV/Rousettus/LP/RSA/UP8705/2018		F	SA	85.1	70	9.666	Not-active	Not pregnant	Non-sclerotized teats
UP 8706	SMC 643	Rectal swab	7/2/2018	0						F	SA	86.6	83	11.067	Not-active	Not pregnant	Non-sclerotized teats
UP 8707	SMC 644	Rectal swab	7/2/2018	0						M	SA	87	77	10.173	Not-active	Not scrotal	Not applicable
UP 8709	SMC 646	Rectal swab	7/2/2018	0						M	SA	82.1	67	9.940	Not-active	Not scrotal	Not applicable
UP 8710	SMC 647	Rectal swab	7/2/2018	0						M	SA	84.3	73	10.272	Not-active	Not scrotal	Not applicable
UP 8711	SMC 648	Rectal swab	7/2/2018	0						M	SA	85.3	72	9.895	Not-active	Not scrotal	Not applicable
UP 8712	SMC 649	Rectal swab	7/2/2018	0						M	SA	83.4	70	10.064	Not-active	Not scrotal	Not applicable
UP 8713	SMC 650	Rectal swab	7/2/2018	0						M	SA	85.8	80	10.867	Not-active	Not scrotal	Not applicable
UP 8714	SMC 651	Rectal swab	7/2/2018	0						M	SA	87.9	86	11.131	Not-active	Not scrotal	Not applicable
UP 8881	SMC 706	Rectal swab	10/4/2018	0						F	SA	87.5	81	10.580	Not-active	Not pregnant	Non-sclerotized teats
UP 8882	SMC 707	Rectal swab	10/4/2018	0						F	SA	88.8	97	12.301	Not-active	Not pregnant	Non-sclerotized teats
UP 8883	SMC 708	Rectal swab	10/4/2018	1	Beta	2	MZ547581	BtCoV/Rousettus/LP/RSA/UP8883/2018		F	SA	78.2	82	13.409	Not-active	Not pregnant	Non-sclerotized teats
UP 8884	SMC 709	Rectal swab	10/4/2018	0						F	SA	80.9	68	10.390	Not-active	Not pregnant	Non-sclerotized teats
UP 8885	SMC 710	Rectal swab	10/4/2018	1	Beta	2	MZ547582	BtCoV/Rousettus/LP/RSA/UP8885/2018		M	SA	87.5	45	5.878	Not-active	Not scrotal	Not applicable
UP 8886	SMC 711	Rectal swab	10/4/2018	1	Beta	2	MZ547583	BtCoV/Rousettus/LP/RSA/UP8886/2018		F	SA	86.5	51	6.816	Not-active	Not pregnant	Non-sclerotized teats
UP 8887	SMC 712	Rectal swab	10/4/2018	0						M	SA	81.5	81	12.195	Not-active	Not scrotal	Not applicable
UP 8888	SMC 713	Rectal swab	10/4/2018	0						M	SA	88.7	94	11.948	Not-active	Not scrotal	Not applicable
UP 8889	SMC 714	Rectal swab	10/4/2018	0						F	SA	84.2	98	13.823	Not-active	Not pregnant	Non-sclerotized teats
UP 8891	SMC 715	Rectal swab	10/4/2018	0						M	SA	86.9	100	13.242	Not-active	Not scrotal	Not applicable

UP 8892	SMC 716	Rectal swab	10/4/2018	1	Beta	2	MZ547584	BtCoV/Rousettus/LP/RSA/UP8892/2018		F	SA	82	95	14.128	Not-active	Not pregnant	Non-sclerotized teats
UP 8893	SMC 717	Rectal swab	10/4/2018	0						F	SA	82.2	87	12.876	Not-active	Not pregnant	Non-sclerotized teats
UP 8894	SMC 718	Rectal swab	10/4/2018	0						F	SA	81.6	76	11.414	Not-active	Not pregnant	Non-sclerotized teats
UP 8895	SMC 719	Rectal swab	10/4/2018	1	Beta	2	MZ547585	BtCoV/Rousettus/LP/RSA/UP8895/2018		M	SA	86.2	76	10.228	Not-active	Not scrotal	Not applicable
UP 8896	SMC 720	Rectal swab	10/4/2018	0						M	SA	85.9	82	11.113	Not-active	Not scrotal	Not applicable
UP 8897	-	Rectal swab	10/4/2018	1	Beta	2	MZ547587	BtCoV/Rousettus/LP/RSA/UP8897/2018		F	SA	84.9	91	12.625	Not-active	Not pregnant	Non-sclerotized teats
UP 8898	-	Rectal swab	10/4/2018	1	Beta	2	MZ547586	BtCoV/Rousettus/LP/RSA/UP8898/2018		M	SA	81.8	75	11.209	Not-active	Not scrotal	Not applicable
UP 8899	-	Rectal swab	10/4/2018	0						F	SA	85.1	81	11.185	Not-active	Not pregnant	Non-sclerotized teats
UP 8900	-	Rectal swab	10/4/2018	0						F	SA	85.9	91	12.333	Not-active	Not pregnant	Non-sclerotized teats
UP 8901	-	Rectal swab	10/4/2018	1	Beta	2	MZ547589	BtCoV/Rousettus/LP/RSA/UP8901/2018		F	SA	84.8	91	12.655	Not-active	Not pregnant	Non-sclerotized teats
UP 8902	-	Rectal swab	10/4/2018	0						F	SA	85	94	13.010	Not-active	Not pregnant	Non-sclerotized teats
UP 8903	-	Rectal swab	10/4/2018	0						M	SA	84.5	85	11.904	Not-active	Not scrotal	Not applicable
UP 8904	-	Rectal swab	10/4/2018	1	Beta	2	MZ547588	BtCoV/Rousettus/LP/RSA/UP8904/2018		M	SA	86.7	95	12.638	Not-active	Not scrotal	Not applicable
UP 8905	-	Rectal swab	10/4/2018	0						M	SA	83.6	86	12.305	Not-active	Not scrotal	Not applicable
UP 8906	SMC 721	Rectal swab	10/4/2018	1	Beta	2	MZ547590	BtCoV/Rousettus/LP/RSA/UP8906/2018		F	SA	85.4	80	10.969	Not-active	Not pregnant	Non-sclerotized teats
UP 8907	SMC 722	Rectal swab	10/4/2018	1	Beta	2	MZ547591	BtCoV/Rousettus/LP/RSA/UP8907/2018		M	SA	84.8	71	9.873	Not-active	Not scrotal	Not applicable
UP 8908	-	Rectal swab	10/4/2018	0						M	SA	84.7	90	12.545	Not-active	Not scrotal	Not applicable
UP 8910	SMC 724	Rectal swab	10/4/2018	0						M	SA	85	102	14.118	Not-active	Not scrotal	Not applicable
UP 8912	SMC 726	Rectal swab	10/4/2018	1	Beta	2	MZ547592	BtCoV/Rousettus/LP/RSA/UP8912/2018		F	SA	86	87	11.763	Not-active	Not pregnant	Non-sclerotized teats
UP 8913	SMC 727	Rectal swab	10/4/2018	1	Beta	2	MZ547593	BtCoV/Rousettus/LP/RSA/UP8913/2018		M	SA	86.4	101	13.530	Not-active	Not scrotal	Not applicable
UP 8914	SMC 728	Rectal swab	10/4/2018	0						M	A	89.5	92	11.485	Active	Not scrotal	Not applicable
UP 8915	SMC 729	Rectal swab	10/4/2018	0						M	SA	81.9	78	11.629	Not-active	Not scrotal	Not applicable
UP 8916	SMC 730	Rectal swab	10/4/2018	1	Beta	2	MZ547596	BtCoV/Rousettus/LP/RSA/UP8916/2018		M	SA	82	72	10.708	Not-active	Not scrotal	Not applicable
UP 8917	SMC 731	Rectal swab	10/4/2018	1	Beta	2	MZ547568	BtCoV/Rousettus/LP/RSA/UP8917/2018		M	SA	84.8	82	11.403	Not-active	Not scrotal	Not applicable
UP 8918	SMC 732	Rectal swab	10/4/2018	1	Beta	2	MZ547594	BtCoV/Rousettus/LP/RSA/UP8918/2018		M	SA	88.2	100	12.855	Not-active	Not scrotal	Not applicable
UP 8919	SMC 733	Rectal swab	10/4/2018	1	Beta	2	MZ547595	BtCoV/Rousettus/LP/RSA/UP8919/2018		M	SA	83	76	11.032	Not-active	Not scrotal	Not applicable
UP 8920	SMC 734	Rectal swab	10/4/2018	1	Beta	2	MZ547597	BtCoV/Rousettus/LP/RSA/UP8920/2018		M	SA	83.4	81	11.645	Not-active	Not scrotal	Not applicable
UP 8921	SMC 735	Rectal swab	10/4/2018	0						M	SA	80.4	78	12.067	Not-active	Not scrotal	Not applicable
UP 8922	SMC 736	Rectal swab	10/4/2018	1	Beta	2	MZ547598	BtCoV/Rousettus/LP/RSA/UP8922/2018		M	SA	82	71	10.559	Not-active	Not scrotal	Not applicable
UP 8923	SMC 737	Rectal swab	10/4/2018	1	Beta	2	MZ547599	BtCoV/Rousettus/LP/RSA/UP8923/2018		M	SA	86.7	94	12.505	Not-active	Not scrotal	Not applicable
UP 8924	SMC 738	Rectal swab	10/4/2018	1	Beta	2	MZ547600	BtCoV/Rousettus/LP/RSA/UP8924/2018		F	SA	84.7	79	11.012	Not-active	Not pregnant	Non-sclerotized teats
UP 8925	SMC 739	Rectal swab	10/4/2018	0						F	SA	87.5	84	10.971	Not-active	Not pregnant	Non-sclerotized teats
UP 8926	SMC 740	Rectal swab	10/4/2018	0						F	SA	88.6	93	11.847	Not-active	Not pregnant	Non-sclerotized teats
UP 8927	SMC 741	Rectal swab	10/4/2018	1	Beta	2	MZ547601	BtCoV/Rousettus/LP/RSA/UP8927/2018		F	SA	88.2	95	12.212	Not-active	Not pregnant	Non-sclerotized teats
UP 8928	SMC 742	Rectal swab	10/4/2018	0						F	SA	83.1	79	11.440	Not-active	Not pregnant	Non-sclerotized teats
UP 8930	SMC 743	Rectal swab	10/4/2018	0						M	A	91.7	136	16.173	Active	Scrotal	Not applicable
UP 8938	SMC 744	Rectal swab	10/4/2018	0						F	SA	77	78	13.156	Not-active	Not pregnant	Non-sclerotized teats
UP 8939	SMC 745	Rectal swab	10/4/2018	1	Beta	2	MZ547602	BtCoV/Rousettus/LP/RSA/UP8939/2018		F	A	89.4	93	11.636	Active	Not pregnant	Non-sclerotized teats
UP 8940	SMC 746	Rectal swab	10/4/2018	0						M	SA	88.5	93	11.874	Not-active	Not scrotal	Not applicable
UP 8942	SMC 747	Rectal swab	10/4/2018	0						M	SA	80.1	77	12.001	Not-active	Not scrotal	Not applicable
UP 8943	SMC 748	Rectal swab	10/4/2018	0						F	SA	87.8	90	11.675	Not-active	Not pregnant	Non-sclerotized teats
UP 8944	SMC 749	Rectal swab	10/4/2018	1	Beta	2	MZ547603	BtCoV/Rousettus/LP/RSA/UP8944/2018		F	A	91	85.5	10.325	Active	Not pregnant	Non-sclerotized teats
UP 8945	SMC 750	Rectal swab	10/4/2018	0						M	SA	88.8	92	11.667	Not-active	Not scrotal	Not applicable
UP 8946	SMC 751	Rectal swab	10/4/2018	1	Beta	2	MZ547604	BtCoV/Rousettus/LP/RSA/UP8946/2018		F	SA	87.2	90	11.836	Not-active	Not pregnant	Non-sclerotized teats
UP 8947	SMC 752	Rectal swab	10/4/2018	0						F	SA	81	75	11.431	Not-active	Not pregnant	Non-sclerotized teats
UP 8948	SMC 753	Rectal swab	10/4/2018	0						F	SA	88.4	91	11.645	Not-active	Not pregnant	Non-sclerotized teats
UP 8949	SMC 754	Rectal swab	10/4/2018	0						M	SA	87.2	85	11.179	Not-active	Not scrotal	Not applicable
UP 8950	SMC 755	Rectal swab	10/4/2018	0						F	SA	83	86	12.484	Not-active	Not pregnant	Non-sclerotized teats
UP 8951	SMC 756	Rectal swab	10/4/2018	0						M	SA	81.7	84	12.584	Not-active	Not scrotal	Not applicable
UP 8952	SMC 757	Rectal swab	10/4/2018	0						F	A	94.4	128	14.364	Active	Not pregnant	Sclerotized teats
UP 8953	SMC 758	Rectal swab	10/4/2018	0						M	A	97.7	152	15.924	Active	Scrotal	Not applicable
UP 8954	SMC 759	Rectal swab	10/4/2018	0						F	A	98.2	126	13.066	Active	Lactating	Sclerotized teats
UP 8959	-	Rectal swab	8/5/2018	0						M	SA	81	74	11.279	Not-active	Not scrotal	Not applicable
UP 8960	-	Rectal swab	8/5/2018	1	Alpha	1	MZ547461	BtCoV/Rousettus/LP/RSA/UP8960/2018		F	SA	83.7	73	10.420	Not-active	Not pregnant	Non-sclerotized teats
UP 8962	-	Rectal swab	8/5/2018	0						F	A	93.5	120	13.726	Active	Not pregnant	Sclerotized teats
UP 8963	-	Rectal swab	8/5/2018	0						F	SA	86.1	88	11.871	Not-active	Not pregnant	Non-sclerotized teats
UP 8967	SMC 760	Rectal swab	8/5/2018	0						F	SA	82.4	86	12.666	Not-active	Not pregnant	Non-sclerotized teats
UP 8969	SMC 762	Rectal swab	8/5/2018	0						F	SA	88.2	87	11.184	Not-active	Not pregnant	Non-sclerotized teats
UP 8972	SMC 764	Rectal swab	8/5/2018	0						F	SA	87.9	94	12.166	Not-active	Not pregnant	Non-sclerotized teats
UP 8973	SMC 765	Rectal swab	8/5/2018	0						F	A	93.3	113	12.981	Active	Not pregnant	Sclerotized teats
UP 8974	SMC 766	Rectal swab	8/5/2018	0						F	SA	85.1	80	11.047	Not-active	Not pregnant	Non-sclerotized teats
UP 8975	SMC 767	Rectal swab	8/5/2018	0						F	SA	84.1	78	11.028	Not-active	Not pregnant	Non-sclerotized teats
UP 8976	SMC 768	Rectal swab	8/5/2018	0						M	A	96.2	148	15.992	Active	Scrotal	Not applicable
UP 8978	SMC 770	Rectal swab	8/5/2018	0						F	A	90.2	114	14.012	Active	Not pregnant	Sclerotized teats
UP 8981	-	Rectal swab	8/5/2018	1	Alpha	1	MZ547462	BtCoV/Rousettus/LP/RSA/UP8981/2018		M	SA	86.7	90	11.973	Not-active	Not scrotal	Not applicable
UP 8989	SMC 778	Rectal swab	8/5/2018	0						M	SA	88	97	12.526	Not-active	Not scrotal	Not applicable
UP 8991	SMC 780	Rectal swab	8/5/2018	0						M	SA	87.4	86	11.258	Not-active	Not scrotal	Not applicable
UP 8992	SMC 781	Rectal swab	8/5/2018	0						M	SA	84.8	92	12.794	Not-active	Not scrotal	Not applicable
UP 8993	SMC 782	Rectal swab	8/5/2018	0						M	SA	82	76	11.303	Not-active	Not scrotal	Not applicable

UP 8994	SMC 783	Rectal swab	8/5/2018	0						M	SA	88.6	87	11.083	Not-active	Not scrotal	Not applicable
UP 8998	SMC 787	Rectal swab	8/5/2018	0						M	A	96.1	160	17.325	Active	Scrotal	Not applicable
UP 9000	SMC 789	Rectal swab	8/5/2018	0						M	A	92	148	17.486	Active	Scrotal	Not applicable
UP 9003	SMC 792	Rectal swab	8/5/2018	1	Beta	2	MZ547605	BtCoV/Rousettus/LP/RSA/UP9003/2018		M	SA	81	70	10.669	Not-active	Not scrotal	Not applicable
UP 9004	SMC 793	Rectal swab	8/5/2018	0						F	A	90	85	10.494	Active	Not pregnant	Non-sclerotized teats
UP 9005	SMC 794	Rectal swab	8/5/2018	0						F	A	90.1	81	9.978	Active	Not pregnant	Non-sclerotized teats
UP 9007	SMC 796	Rectal swab	8/5/2018	0						F	SA	86.6	81	10.801	Not-active	Not pregnant	Non-sclerotized teats
UP 9008	SMC 797	Rectal swab	8/5/2018	0						F	SA	83	85	12.339	Not-active	Not pregnant	Non-sclerotized teats
UP 9009	SMC 798	Rectal swab	8/5/2018	0						F	SA	81.4	80	12.074	Not-active	Not pregnant	Non-sclerotized teats
UP 9010	SMC 799	Rectal swab	8/5/2018	0						M	A	94.5	146	16.349	Active	Scrotal	Not applicable
UP 9011	SMC 800	Rectal swab	8/5/2018	0						M	A	97.5	138	14.517	Active	Scrotal	Not applicable
UP 9012	SMC 801	Rectal swab	8/5/2018	0						M	A	90.5	146	17.826	Active	Scrotal	Not applicable
UP 9023	SMC 802	Rectal swab	8/5/2018	0						M	A	91	110	13.283	Active	Scrotal	Not applicable
UP 9024	SMC 803	Rectal swab	8/5/2018	0						M	A	96.5	115	12.349	Active	Scrotal	Not applicable
UP 9025	SMC 804	Rectal swab	8/5/2018	0						M	A	96.4	139	14.958	Active	Scrotal	Not applicable
UP 9653	-	Rectal swab	5/6/2018	0						M	SA	83.9	78	11.081	Not-active	Not scrotal	Not applicable
UP 9654	-	Rectal swab	5/6/2018	0						M	SA	86	87	11.763	Not-active	Not scrotal	Not applicable
UP 9655	-	Rectal swab	5/6/2018	0						M	SA	85.6	84	11.464	Not-active	Not scrotal	Not applicable
UP 9656	-	Rectal swab	5/6/2018	0						M	SA	87	91	12.023	Not-active	Not scrotal	Not applicable
UP 9657	-	Rectal swab	5/6/2018	0						M	SA	83.3	72	10.376	Not-active	Not scrotal	Not applicable
UP 9658	SMC 760 (m)	Rectal swab	5/6/2018	0						M	SA	83	76	11.032	Not-active	Not scrotal	Not applicable
UP 9659	SMC 761 (m)	Rectal swab	5/6/2018	0						M	SA	85.9	85	11.519	Not-active	Not scrotal	Not applicable
UP 9660	SMC 762 (m)	Rectal swab	5/6/2018	0						M	A	94.8	150	16.691	Active	Scrotal	Not applicable
UP 9661	SMC 763 (m)	Rectal swab	5/6/2018	0						M	SA	87.7	92	11.962	Not-active	Not scrotal	Not applicable
UP 9662	SMC 764 (m)	Rectal swab	5/6/2018	0						M	A	89.1	86	10.833	Active	Not scrotal	Not applicable
UP 9663	SMC 765 (m)	Rectal swab	5/6/2018	0						M	SA	86.9	91	12.050	Not-active	Not scrotal	Not applicable
UP 9664	SMC 766 (m)	Rectal swab	5/6/2018	0						M	SA	86.4	91	12.190	Not-active	Not scrotal	Not applicable
UP 9665	-	Rectal swab	5/6/2018	0						M	SA	84.9	87	12.070	Not-active	Not scrotal	Not applicable
UP 9666	SMC 767 (m)	Rectal swab	5/6/2018	0						M	SA	85.5	89	12.175	Not-active	Not scrotal	Not applicable
UP 9667	SMC 768 (m)	Rectal swab	5/6/2018	0						M	SA	85.3	80	10.995	Not-active	Not scrotal	Not applicable
UP 9668	-	Rectal swab	5/6/2018	0						F	SA	83.8	68	9.683	Not-active	Not pregnant	Non-sclerotized teats
UP 9669	-	Rectal swab	5/6/2018	0						F	SA	81.7	67	10.038	Not-active	Not pregnant	Non-sclerotized teats
UP 9670	-	Rectal swab	5/6/2018	1	Beta	2	MZ547606	BtCoV/Rousettus/LP/RSA/UP9670/2018		F	SA	82.7	81	11.843	Not-active	Not pregnant	Non-sclerotized teats
UP 9671	-	Rectal swab	5/6/2018	1	Beta	2	MZ547607	BtCoV/Rousettus/LP/RSA/UP9671/2018		F	SA	81.9	71	10.585	Not-active	Not pregnant	Non-sclerotized teats
UP 9672	-	Rectal swab	5/6/2018	0						F	SA	83.9	82	11.649	Not-active	Not pregnant	Non-sclerotized teats
UP 9673	SMC 769 (m)	Rectal swab	5/6/2018	0						M	SA	86	88	11.898	Not-active	Not scrotal	Not applicable
UP 9674	SMC 770 (m)	Rectal swab	5/6/2018	0						F	SA	87.4	88	11.520	Not-active	Not pregnant	Non-sclerotized teats
UP 9675	-	Rectal swab	5/6/2018	0						F	A	91.4	95	11.372	Active	Not pregnant	Non-sclerotized teats
UP 9676	SMC 772 (m)	Rectal swab	5/6/2018	0						F	SA	85.2	83	11.434	Not-active	Not pregnant	Non-sclerotized teats
UP 9677	SMC 773 (m)	Rectal swab	5/6/2018	0						F	SA	87.7	83	10.791	Not-active	Not pregnant	Non-sclerotized teats
UP 9678	SMC 774 (m)	Rectal swab	5/6/2018	1	Beta	2	MZ547608	BtCoV/Rousettus/LP/RSA/UP9678/2018		F	SA	84.3	78	10.976	Not-active	Not pregnant	Non-sclerotized teats
UP 9679	SMC 775 (m)	Rectal swab	5/6/2018	0						F	SA	86.1	76	10.252	Not-active	Not pregnant	Non-sclerotized teats
UP 9680	SMC 776 (m)	Rectal swab	5/6/2018	0						F	A	96.2	114	12.318	Active	Not pregnant	Sclerotized teats
UP 9681	SMC 777 (m)	Rectal swab	5/6/2018	0						F	SA	83	75	10.887	Not-active	Not pregnant	Non-sclerotized teats
UP 9682	SMC 778 (m)	Rectal swab	5/6/2018	0						F	SA	83.3	72	10.376	Not-active	Not pregnant	Non-sclerotized teats
UP 9683	SMC 779 (m)	Rectal swab	5/6/2018	0						F	SA	83.5	78	11.187	Not-active	Not pregnant	Non-sclerotized teats
UP 9684	SMC 780 (m)	Rectal swab	5/6/2018	0						F	SA	81.6	75	11.264	Not-active	Not pregnant	Non-sclerotized teats
UP 9685	SMC 781 (m)	Rectal swab	5/6/2018	1	Beta	2	MZ547609	BtCoV/Rousettus/LP/RSA/UP9685/2018		F	SA	85.3	74	10.170	Not-active	Not pregnant	Non-sclerotized teats
UP 9686	SMC 782 (m)	Rectal swab	5/6/2018	0						M	A	99.5	105	10.606	Active	Scrotal	Not applicable
UP 9687	SMC 783 (m)	Rectal swab	5/6/2018	0						M	A	97.4	164	17.287	Active	Scrotal	Not applicable
UP 9688	SMC 785 (m)	Rectal swab	5/6/2018	0						M	A	92	144	17.013	Active	Scrotal	Not applicable
UP 9692	SMC 804	Rectal swab	5/6/2018	0						M	A	91.7	149	17.719	Active	Scrotal	Not applicable
UP 9693	SMC 805	Rectal swab	5/6/2018	0						M	A	94.9	149	16.545	Active	Scrotal	Not applicable
UP 9694	SMC 806	Rectal swab	5/6/2018	0						M	A	94	153	17.316	Active	Scrotal	Not applicable
UP 9695	SMC 807	Rectal swab	5/6/2018	0						M	A	93	141	16.302	Active	Scrotal	Not applicable
UP 9696	SMC 808	Rectal swab	5/6/2018	0						M	A	95.9	158	17.180	Active	Scrotal	Not applicable
UP 9697	SMC 809	Rectal swab	5/6/2018	0						F	A	95.8	115	12.530	Active	Not pregnant	Sclerotized teats
UP 9700	SMC 810	Rectal swab	3/7/2018	0						M	A	95.5	124	13.596	Active	Scrotal	Not applicable
UP 9701	-	Rectal swab	3/7/2018	0						M	SA	86.4	75	10.047	Not-active	Not scrotal	Not applicable
UP 9704	-	Rectal swab	3/7/2018	0						M	SA	86.9	91	12.050	Not-active	Not scrotal	Not applicable
UP 9705	-	Rectal swab	3/7/2018	0						M	SA	84.5	84	11.764	Not-active	Not scrotal	Not applicable
UP 9706	-	Rectal swab	3/7/2018	0						F	SA	74.2	69	12.533	Not-active	Not pregnant	Non-sclerotized teats
UP 9707	-	Rectal swab	3/7/2018	0						M	SA	85.6	85	11.600	Not-active	Not scrotal	Not applicable
UP 9708	SMC 811	Rectal swab	3/7/2018	0						M	A	91.3	90	10.797	Active	Not scrotal	Not applicable
UP 9709	SMC 812	Rectal swab	3/7/2018	0						M	A	92.4	109	12.767	Active	Not scrotal	Not applicable
UP 9712	-	Rectal swab	3/7/2018	0						F	SA	85.2	79	10.883	Not-active	Not pregnant	Non-sclerotized teats
UP 9713	-	Rectal swab	3/7/2018	0						F	SA	78.6	66	10.683	Not-active	Not pregnant	Non-sclerotized teats
UP 9714	-	Rectal swab	3/7/2018	0						F	SA	84.6	79	11.038	Not-active	Not pregnant	Non-sclerotized teats
UP 9715	-	Rectal swab	3/7/2018	0						F	SA	83.6	77	11.017	Not-active	Not pregnant	Non-sclerotized teats

UP 9716	SMC 813	Rectal swab	3/7/2018	0						F	SA	85.6	80	10.918	Not-active	Not pregnant	Non-sclerotized teats
UP 9717	SMC 814	Rectal swab	3/7/2018	1	Alpha	1	MZ547452	BtCoV/Rousettus/LP/RSA/UP9717/2018		F	SA	83.6	78	11.160	Not-active	Not pregnant	Non-sclerotized teats
UP 9718	-	Rectal swab	3/7/2018	0						M	SA	83.8	69	9.826	Not-active	Not scrotal	Not applicable
UP 9719	SMC 815	Rectal swab	3/7/2018	0						M	A	94.3	124	13.944	Active	Not scrotal	Not applicable
UP 9720	SMC 816	Rectal swab	3/7/2018	0						F	A	91.3	115	13.796	Active	Not pregnant	Sclerotized teats
UP 9721	SMC 817	Rectal swab	3/7/2018	0						M	SA	86.8	91	12.078	Not-active	Not scrotal	Not applicable
UP 9722	SMC 818	Rectal swab	3/7/2018	0						M	SA	82.5	71	10.432	Not-active	Not scrotal	Not applicable
UP 9723	SMC 819	Rectal swab	3/7/2018	0						M	SA	88.3	79	10.132	Not-active	Not scrotal	Not applicable
UP 9724	SMC 820	Rectal swab	3/7/2018	0						F	SA	84.8	79	10.986	Not-active	Not pregnant	Non-sclerotized teats
UP 9725	SMC 821	Rectal swab	3/7/2018	0						M	A	97.1	133	14.106	Active	Scrotal	Not applicable
UP 9726	SMC 822	Rectal swab	3/7/2018	0						M	SA	81.1	71	10.795	Not-active	Not scrotal	Not applicable
UP 9727	SMC 823	Rectal swab	3/7/2018	1	Beta	3	MZ547502	BtCoV/Rousettus/LP/RSA/UP9727/2018		M	SA	79.1	77	12.307	Not-active	Not scrotal	Not applicable
UP 9728	SMC 824	Rectal swab	3/7/2018	0						M	SA	83.3	64	9.223	Not-active	Not scrotal	Not applicable
UP 9729	SMC 825	Rectal swab	3/7/2018	0						F	SA	87.1	79	10.413	Not-active	Not pregnant	Non-sclerotized teats
UP 9730	SMC 826	Rectal swab	3/7/2018	0						F	SA	82.5	77	11.313	Not-active	Not pregnant	Non-sclerotized teats
UP 9731	SMC 827	Rectal swab	3/7/2018	0						F	SA	82.4	75	11.046	Not-active	Not pregnant	Non-sclerotized teats
UP 9732	SMC 828	Rectal swab	3/7/2018	0						M	A	95	69	7.645	Active	Not scrotal	Not applicable
UP 9733	SMC 829	Rectal swab	3/7/2018	0						F	A	91.3	61	7.318	Active	Not pregnant	Sclerotized teats
UP 9734	SMC 830	Rectal swab	3/7/2018	0						F	A	90.2	53	6.514	Active	Not pregnant	Sclerotized teats
UP 9735	SMC 831	Rectal swab	3/7/2018	0						F	A	94.6	66	7.375	Active	Not pregnant	Sclerotized teats
UP 9736	SMC 832	Rectal swab	3/7/2018	0						F	A	92	69	8.152	Active	Not pregnant	Sclerotized teats
UP 9737	SMC 833	Rectal swab	3/7/2018	0						F	A	93.6	68	7.762	Active	Not pregnant	Sclerotized teats
UP 9738	SMC 834	Rectal swab	3/7/2018	0						F	A	95.4	62	6.812	Active	Not pregnant	Sclerotized teats
UP 9739	SMC 835	Rectal swab	3/7/2018	0						F	A	93.9	78	8.846	Active	Not pregnant	Sclerotized teats
UP 9740	SMC 836	Rectal swab	3/7/2018	0						M	A	91.6	85	10.130	Active	Scrotal	Not applicable
UP 9741	SMC 837	Rectal swab	3/7/2018	0						M	A	96.7	59	6.310	Active	Scrotal	Not applicable
UP 9742	SMC 838	Rectal swab	3/7/2018	0						M	A	94.4	51	5.723	Active	Scrotal	Not applicable
UP 9743	SMC 839	Rectal swab	3/7/2018	0						M	A	96.5	61	6.551	Active	Scrotal	Not applicable
UP 9758	-	Rectal swab	1/8/2018	0						F	SA	87.4	88	11.520	Not-active	Not pregnant	Non-sclerotized teats
UP 9759	-	Rectal swab	1/8/2018	0						M	SA	85.9	94	12.739	Not-active	Not scrotal	Not applicable
UP 9760	SMC 840	Rectal swab	1/8/2018	0						M	A	96.3	129	13.910	Active	Scrotal	Not applicable
UP 9761	SMC 841	Rectal swab	1/8/2018	0						F	A	92.6	112	13.062	Active	Pregnant	Sclerotized teats
UP 9772	-	Rectal swab	1/8/2018	0						M	A	89.6	95	11.833	Active	Not scrotal	Not applicable
UP 9773	SMC 842	Rectal swab	1/8/2018	0						M	A	99	133	13.570	Active	Scrotal	Not applicable
UP 9775	-	Rectal swab	1/8/2018	0						F	SA	82.1	64	9.495	Not-active	Not pregnant	Non-sclerotized teats
UP 9776	-	Rectal swab	1/8/2018	0						F	A	90.3	83	10.179	Active	Not pregnant	Non-sclerotized teats
UP 9779	-	Rectal swab	1/8/2018	0						M	SA	88.8	88	11.160	Not-active	Not scrotal	Not applicable
UP 9782	-	Rectal swab	1/8/2018	0						M	SA	86.8	82	10.884	Not-active	Not scrotal	Not applicable
UP 9783	SMC 844	Rectal swab	1/8/2018	0						M	SA	88.5	89	11.363	Not-active	Not scrotal	Not applicable
UP 9784	SMC 845	Rectal swab	1/8/2018	0						F	A	98.6	124	12.755	Active	Not pregnant	Sclerotized teats
UP 9785	SMC 846	Rectal swab	1/8/2018	0						F	A	95.9	123	13.374	Active	Pregnant	Sclerotized teats
UP 9786	SMC 847	Rectal swab	1/8/2018	0						F	A	93.9	96	10.888	Active	Not pregnant	Sclerotized teats
UP 9787	SMC 848	Rectal swab	1/8/2018	0						F	A	92.1	106	12.496	Active	Not pregnant	Sclerotized teats
UP 9788	SMC 849	Rectal swab	1/8/2018	0						F	A	93.9	117	13.270	Active	Not pregnant	Sclerotized teats
UP 9789	SMC 850	Rectal swab	1/8/2018	0						M	A	95.8	123	13.402	Active	Scrotal	Not applicable
UP 9790	SMC 851	Rectal swab	1/8/2018	0						M	A	96	124	13.455	Active	Scrotal	Not applicable
UP 9791	SMC 852	Rectal swab	1/8/2018	0						M	A	97.6	124	13.017	Active	Scrotal	Not applicable
UP 9792	SMC 853	Rectal swab	1/8/2018	0						M	A	97.6	126	13.227	Active	Not scrotal	Not applicable
UP 9793	SMC 854	Rectal swab	1/8/2018	0						M	A	98.1	132	13.716	Active	Scrotal	Not applicable
UP 9796	SMC 855	Rectal swab	1/8/2018	0						F	A	94.9	113	12.547	Active	Not pregnant	Sclerotized teats
UP 9797	SMC 856	Rectal swab	1/8/2018	0						F	A	92.9	119	13.788	Active	Pregnant	Sclerotized teats
UP 9798	SMC 857	Rectal swab	1/8/2018	0						M	SA	85.4	86	11.792	Not-active	Not scrotal	Not applicable
UP 9799	SMC 858	Rectal swab	1/8/2018	0						M	A	96.7	125	13.368	Active	Scrotal	Not applicable
UP 9800	SMC 859	Rectal swab	1/8/2018	0						F	A	91.8	120	14.240	Active	Pregnant	Sclerotized teats
UP 9801	SMC 860	Rectal swab	1/8/2018	1	Beta	3	MZ547503	BtCoV/Rousettus/LP/RSA/UP9801/2018		F	A	91	107	12.921	Active	Pregnant	Sclerotized teats
UP 9802	SMC 861	Rectal swab	1/8/2018	0						F	A	90.3	127	15.575	Active	Pregnant	Sclerotized teats
UP 9808	SMC 862	Rectal swab	1/8/2018	0						M	A	89	85	10.731	Active	Not scrotal	Not applicable
UP 9809	SMC 863	Rectal swab	1/8/2018	0						F	SA	85.2	73	10.056	Not-active	Not pregnant	Non-sclerotized teats
UP 9810	SMC 864	Rectal swab	1/8/2018	0						F	SA	84.1	76	10.745	Not-active	Not pregnant	Non-sclerotized teats
UP 9811	SMC 865	Rectal swab	1/8/2018	0						F	SA	88.6	90	11.465	Not-active	Not pregnant	Non-sclerotized teats
UP 9812	SMC 866	Rectal swab	1/8/2018	0						F	SA	88.3	85	10.902	Not-active	Not pregnant	Non-sclerotized teats
UP 9813	SMC 867	Rectal swab	1/8/2018	0						F	SA	88	72	9.298	Not-active	Not pregnant	Non-sclerotized teats
UP 9814	SMC 868	Rectal swab	1/8/2018	0						F	SA	85.1	87	12.013	Not-active	Not pregnant	Non-sclerotized teats
UP 9815	SMC 869	Rectal swab	1/8/2018	0						F	A	89.2	80	10.054	Active	Not pregnant	Non-sclerotized teats
UP 9816	SMC 870	Rectal swab	1/8/2018	0						F	SA	80.7	78	11.977	Not-active	Not pregnant	Non-sclerotized teats
UP 9817	SMC 871	Rectal swab	1/8/2018	0						F	SA	84	81	11.480	Not-active	Not pregnant	Non-sclerotized teats
UP 9818	SMC 872	Rectal swab	1/8/2018	1	Beta	3	MZ547504	BtCoV/Rousettus/LP/RSA/UP9818/2018		F	A	98.4	116	11.980	Active	Not pregnant	Sclerotized teats
UP 9819	SMC 873	Rectal swab	1/8/2018	0						M	SA	83.2	77	11.124	Not-active	Not scrotal	Not applicable
UP 9820	SMC 874	Rectal swab	1/8/2018	0						M	SA	87.3	83	10.891	Not-active	Not scrotal	Not applicable

UP 9821	SMC 875	Rectal swab	1/8/2018	0						M	A	91.2	85	10.219	Active	Not scrotal	Not applicable
UP 9822	SMC 876	Rectal swab	1/8/2018	0						M	SA	84.7	80	11.151	Not-active	Not scrotal	Not applicable
UP 9823	SMC 877	Rectal swab	1/8/2018	1	Beta	2	MZ547610	BtCoV/Rousettus/LP/RSA/UP9823/2018	Positive	M	SA	81.4	75	11.319	Not-active	Not scrotal	Not applicable
UP 9824	SMC 878	Rectal swab	1/8/2018	1	Beta	2	MZ547612	BtCoV/Rousettus/LP/RSA/UP9824/2018		M	SA	86.8	87	11.547	Not-active	Not scrotal	Not applicable
UP 9825	SMC 879	Rectal swab	1/8/2018	0						M	A	92	86	10.161	Active	Not scrotal	Not applicable
UP 9826	SMC 880	Rectal swab	1/8/2018	0						M	SA	85.1	84	11.599	Not-active	Not scrotal	Not applicable
UP 9827	SMC 881	Rectal swab	1/8/2018	0						M	A	94.9	120	13.324	Active	Scrotal	Not applicable
UP 9828	SMC 882	Rectal swab	1/8/2018	0						M	A	92.7	122	14.197	Active	Scrotal	Not applicable
UP 9823 R	SMC 877	Rectal swab	4/9/2018	1	Beta	2	MZ547611	BtCoV/Rousettus/LP/RSA/UP9823R/2018	Positive/ positive	M	SA	83.4	75	10.783	Not-active	Not scrotal	Not applicable
UP 9971	SMC 883	Rectal swab	4/9/2018	0						F	A	99	121	12.346	Active	Not pregnant	Sclerotized teats
UP 9972	SMC 887	Rectal swab	4/9/2018	0						F	A	94.1	123	13.891	Active	Pregnant	Sclerotized teats
UP 9973	SMC 884	Rectal swab	4/9/2018	0						M	SA	85.2	86	11.847	Not-active	Not scrotal	Not applicable
UP 9974	SMC 885	Rectal swab	4/9/2018	0						F	A	92	116	13.705	Active	Pregnant	Sclerotized teats
UP 9975	SMC 886	Rectal swab	4/9/2018	0						F	A	89.2	89	11.186	Active	Not pregnant	Non-sclerotized teats
UP 9976	SMC 888	Rectal swab	4/9/2018	0						F	SA	87.2	82	10.784	Not-active	Not pregnant	Non-sclerotized teats
UP 9977	SMC 889	Rectal swab	4/9/2018	0						F	SA	88.2	89	11.441	Not-active	Not pregnant	Non-sclerotized teats
UP 9978	SMC 890	Rectal swab	4/9/2018	1	Beta	2	MZ547569	BtCoV/Rousettus/LP/RSA/UP9978/2018		F	A	94.5	118	13.214	Active	Pregnant	Sclerotized teats
UP 9979	SMC 891	Rectal swab	4/9/2018	0						F	A	96.7	123	13.154	Active	Pregnant	Sclerotized teats
UP 9980	SMC 892	Rectal swab	4/9/2018	0						M	A	97.7	143	14.981	Active	Scrotal	Not applicable
UP 9981	SMC 893	Rectal swab	4/9/2018	0						M	SA	87	84	11.098	Not-active	Not scrotal	Not applicable
UP 9982	SMC 894	Rectal swab	4/9/2018	0						F	SA	88.7	92	11.693	Not-active	Not pregnant	Non-sclerotized teats
UP 9984	SMC 895	Rectal swab	4/9/2018	0						M	SA	86.9	85	11.256	Not-active	Not scrotal	Not applicable
UP 9985	SMC 896	Rectal swab	4/9/2018	0						F	A	99.5	126	12.727	Active	Pregnant	Sclerotized teats
UP 9986	SMC 897	Rectal swab	4/9/2018	0						F	A	90.3	98	12.019	Active	Not pregnant	Non-sclerotized teats
UP 9987	SMC 898	Rectal swab	4/9/2018	1	Beta	2	MZ547613	BtCoV/Rousettus/LP/RSA/UP9987/2018		F	A	90.8	90	10.916	Active	Not pregnant	Non-sclerotized teats
UP 9988	SMC 899	Rectal swab	4/9/2018	0						F	A	93.7	109	12.415	Active	Pregnant	Sclerotized teats
UP 9989	SMC 900	Rectal swab	4/9/2018	0						F	SA	80.3	67	10.391	Not-active	Not pregnant	Non-sclerotized teats
UP 9990	SMC 901	Rectal swab	4/9/2018	0						F	A	95.3	115	12.662	Active	Pregnant	Sclerotized teats
UP 9991	SMC 902	Rectal swab	4/9/2018	0						F	A	89.5	97	12.109	Active	Not pregnant	Non-sclerotized teats
UP 9992	SMC 903	Rectal swab	4/9/2018	0						F	A	97.3	122	12.886	Active	Pregnant	Sclerotized teats
UP 9993	SMC 904	Rectal swab	4/9/2018	1	Beta	2	MZ547614	BtCoV/Rousettus/LP/RSA/UP9993/2018		F	A	89.8	90	11.161	Active	Not pregnant	Non-sclerotized teats
UP 9994	SMC 905	Rectal swab	4/9/2018	0						F	A	90.7	95	11.548	Active	Not pregnant	Non-sclerotized teats
UP 9995	SMC 906	Rectal swab	4/9/2018	0						M	A	94.7	125	13.938	Active	Scrotal	Not applicable
UP 9996	SMC 907	Rectal swab	4/9/2018	0						F	SA	87.1	82	10.809	Not-active	Not pregnant	Non-sclerotized teats
UP 9997	SMC 908	Rectal swab	4/9/2018	0						M	A	93	113	13.065	Active	Scrotal	Not applicable
UP 9998	SMC 909	Rectal swab	4/9/2018	0						F	A	93.6	113	12.898	Active	Pregnant	Sclerotized teats
UP 9999	SMC 910	Rectal swab	4/9/2018	0						M	A	97.9	136	14.190	Active	Scrotal	Not applicable
UP 10000	SMC 911	Rectal swab	4/9/2018	0						M	A	91.4	101	12.090	Active	Not scrotal	Not applicable
UP 10001	SMC 912	Rectal swab	4/9/2018	0						M	A	91	117	14.129	Active	Scrotal	Not applicable
UP 10002	SMC 913	Rectal swab	4/9/2018	0						M	A	94.7	128	14.273	Active	Not scrotal	Not applicable
UP 10003	SMC 914	Rectal swab	4/9/2018	0						M	A	96.1	121	13.102	Active	Scrotal	Not applicable
UP 10004	SMC 915	Rectal swab	4/9/2018	0						M	A	94.5	117	13.102	Active	Scrotal	Not applicable
UP 10005	SMC 916	Rectal swab	4/9/2018	0						M	A	92.9	125	14.484	Active	Scrotal	Not applicable
UP 10006	SMC 917	Rectal swab	4/9/2018	0						M	A	92.4	118	13.821	Active	Scrotal	Not applicable
UP 10007	SMC 918	Rectal swab	4/9/2018	0						M	A	99	125	12.754	Active	Scrotal	Not applicable
UP 10008	SMC 919	Rectal swab	4/9/2018	0						M	A	94.9	128	14.213	Active	Scrotal	Not applicable
UP 10009	SMC 920	Rectal swab	4/9/2018	0						M	A	96.1	128	13.860	Active	Not scrotal	Not applicable
UP 10010	SMC 921	Rectal swab	4/9/2018	0					Negative	M	SA	84.5	74	10.364	Not-active	Not scrotal	Not applicable
UP 10011	SMC 922	Rectal swab	4/9/2018	1	Beta	2	MZ547615	BtCoV/Rousettus/LP/RSA/UP10011/2018		M	A	89	84	10.605	Active	Not scrotal	Not applicable
UP 10012	SMC 923	Rectal swab	4/9/2018	0						F	A	89	88	11.110	Active	Not pregnant	Non-sclerotized teats
UP 10013	SMC 924	Rectal swab	4/9/2018	0						F	SA	88.4	85	10.877	Not-active	Not pregnant	Non-sclerotized teats
UP 10014	SMC 925	Rectal swab	4/9/2018	0						M	SA	87	94	12.419	Not-active	Not scrotal	Not applicable
UP 10019	SMC 926	Rectal swab	4/9/2018	0						F	SA	87.4	81	10.604	Not-active	Not pregnant	Non-sclerotized teats
UP 10020	SMC 927	Rectal swab	4/9/2018	0						M	SA	87.3	104	13.646	Not-active	Not scrotal	Not applicable
UP 10021	SMC 928	Rectal swab	4/9/2018	0						M	SA	86	98	13.250	Not-active	Not scrotal	Not applicable
UP 10022	SMC 929	Rectal swab	4/9/2018	0						M	A	90.2	100	12.291	Active	Not scrotal	Not applicable
UP 10023	SMC 930	Rectal swab	4/9/2018	0						M	A	91.9	97	11.485	Active	Not scrotal	Not applicable
UP 10024	SMC 931	Rectal swab	4/9/2018	0						M	SA	84.5	87	12.184	Not-active	Not scrotal	Not applicable
UP 10025	SMC 932	Rectal swab	4/9/2018	0						M	A	101.3	137	13.351	Active	Scrotal	Not applicable
UP 10026	SMC 933	Rectal swab	4/9/2018	0						M	A	93.4	94	10.775	Active	Not scrotal	Not applicable
UP 10027	SMC 934	Rectal swab	4/9/2018	0						M	SA	88.3	86	11.030	Not-active	Not scrotal	Not applicable
UP 10028	SMC 935	Rectal swab	4/9/2018	0						M	SA	84.5	77	10.784	Not-active	Not scrotal	Not applicable
UP 10010	SMC 921	Rectal swab	2/10/2018	0					Negative	M	SA	85.1	90	12.427	Not-active	Not scrotal	Not applicable
UP 10044	SMC 936	Rectal swab	2/10/2018	0						F	A	93.8	124	14.093	Active	Pregnant	Not recorded
UP 10045	SMA 276	Rectal swab	2/10/2018	0						F	A	94	146	16.523	Active	Pregnant	Not recorded
UP 10046	SMC 937	Rectal swab	2/10/2018	0						F	A	89.4	95	11.886	Active	Not pregnant	Non-sclerotized teats
UP 10047	SMC 938	Rectal swab	2/10/2018	0						M	A	98.5	138	14.224	Active	Scrotal	Not applicable
UP 10048	SMC 939	Rectal swab	2/10/2018	0						M	A	99.5	133	13.434	Active	Scrotal	Not applicable
UP 10049	SMC 940	Rectal swab	2/10/2018	0						F	A	97	132	14.029	Active	Pregnant	Not recorded

UP 10050	SMC 941	Rectal swab	2/10/2018	0						F	A	95.7	133	14.522	Active	Pregnant	Not recorded
UP 10051	SMC 942	Rectal swab	2/10/2018	0						F	A	99.4	112	11.336	Active	Pregnant	Not recorded
UP 10052	SMC 943	Rectal swab	2/10/2018	0						F	A	101	141	13.822	Active	Pregnant	Not recorded
UP 10053	SMC 944	Rectal swab	2/10/2018	0						M	A	89.3	103	12.916	Active	Not scrotal	Not applicable
UP 10054	SMC 945	Rectal swab	2/10/2018	0						M	A	92.9	116	13.441	Active	Scrotal	Not applicable
UP 10055	SMC 946	Rectal swab	2/10/2018	0						M	A	97.3	137	14.471	Active	Scrotal	Not applicable
UP 10056	SMC 947	Rectal swab	2/10/2018	0						F	A	92.9	116	13.441	Active	Pregnant	Not recorded
UP 10057	SMC 948	Rectal swab	2/10/2018	0						F	A	90.2	110	13.520	Active	Pregnant	Not recorded
UP 10058	SMC 949	Rectal swab	2/10/2018	0						F	A	93.7	136	15.490	Active	Pregnant	Not recorded
UP 10059	SMC 950	Rectal swab	2/10/2018	1	Alpha	1	MZ547463	BtCoV/Rousettus/LP/RSA/UP10059/2018		F	A	90.8	94	11.401	Active	Pregnant	Non-sclerotized teats
UP 10060	SMC 951	Rectal swab	2/10/2018	0						F	A	89.9	108	13.363	Active	Pregnant	Not recorded
UP 10061	SMC 952	Rectal swab	2/10/2018	0						M	SA	86.8	84	11.149	Not-active	Not scrotal	Not applicable
UP 10062	SMC 953	Rectal swab	2/10/2018	0						M	A	98	135	14.057	Active	Scrotal	Not applicable
UP 10063	SMC 954	Rectal swab	2/10/2018	0						M	A	84.9	86	11.931	Active	Not scrotal	Not applicable
UP 10064	SMC 955	Rectal swab	2/10/2018	0						M	SA	86.7	88	11.707	Not-active	Not scrotal	Not applicable
UP 10065	SMC 956	Rectal swab	2/10/2018	0						F	SA	84.8	73	10.152	Not-active	Not pregnant	Non-sclerotized teats
UP 10066	SMC 957	Rectal swab	2/10/2018	0						F	SA	83.4	73	10.495	Not-active	Not pregnant	Non-sclerotized teats
UP 10067	SMC 958	Rectal swab	2/10/2018	0						M	A	89.7	92	11.434	Active	Not scrotal	Not applicable
UP 10068	SMC 959	Rectal swab	2/10/2018	0						M	SA	88.4	93	11.901	Not-active	Not scrotal	Not applicable
UP 10069	SMC 960	Rectal swab	2/10/2018	0						M	A	91.2	132	15.870	Active	Scrotal	Not applicable
UP 10070	SMC 961	Rectal swab	2/10/2018	0						M	A	93.4	131	15.017	Active	Scrotal	Not applicable
UP 10071	SMC 962	Rectal swab	2/10/2018	0						M	A	92.8	98	11.380	Active	Not scrotal	Not applicable
UP 10072	SMC 963	Rectal swab	2/10/2018	0						M	A	92.5	124	14.492	Active	Scrotal	Not applicable
UP 10073	SMC 964	Rectal swab	2/10/2018	0						F	SA	85.5	78	10.670	Not-active	Not pregnant	Non-sclerotized teats
UP 10074	SMC 965	Rectal swab	2/10/2018	0						M	SA	84.6	102	14.251	Not-active	Not scrotal	Not applicable
UP 10075	SMC 966	Rectal swab	2/10/2018	0						M	SA	86.1	93	12.545	Not-active	Not scrotal	Not applicable
UP 10076	SMC 967	Rectal swab	2/10/2018	0						F	SA	88.7	87	11.058	Not-active	Not pregnant	Non-sclerotized teats
UP 10077	SMC 968	Rectal swab	2/10/2018	0						M	SA	84.5	89	12.465	Not-active	Not scrotal	Not applicable
UP 10078	SMC 969	Rectal swab	2/10/2018	0						F	SA	83	79	11.468	Not-active	Not pregnant	Non-sclerotized teats
UP 10079	SMC 970	Rectal swab	2/10/2018	0						M	A	92.1	102	12.025	Active	Not scrotal	Not applicable
UP 9012	SMC 801	Rectal swab	2/10/2018	0					Negative	M	A	90.2	129	15.855	Active	Scrotal	Not applicable
UP 10109	SMC 971	Rectal swab	6/11/2018	0						F	A	94.2	140	15.777	Active	Lactating	Sclerotized teats
UP 10110	SMC 972	Rectal swab	6/11/2018	0					Negative	F	A	91.5	157	18.752	Active	Pregnant	Not recorded
UP 10111	SMC 973	Rectal swab	6/11/2018	0						F	A	91.4	135	16.160	Active	Lactating	Sclerotized teats
UP 10114	SMC 975	Rectal swab	6/11/2018	0						F	A	92.3	148	17.372	Active	Pregnant	Not recorded
UP 10115	SMC 976	Rectal swab	6/11/2018	0						F	A	89.4	122	15.265	Active	Pregnant	Not recorded
UP 10116	SMC 977	Rectal swab	6/11/2018	0						F	A	93.9	134	15.198	Active	Pregnant	Not recorded
UP 10118	SMC 979	Rectal swab	6/11/2018	0						F	A	90.2	150	18.436	Active	Pregnant	Not recorded
UP 10119	SMC 980	Rectal swab	6/11/2018	0						F	A	94.5	150	16.797	Active	Pregnant	Not recorded
UP 10120	SMC 981	Rectal swab	6/11/2018	0						F	A	95	111	12.299	Active	Pregnant	Not recorded
UP 10121	SMC 982	Rectal swab	6/11/2018	0						M	SA	87.9	96	12.425	Not-active	Not scrotal	Not applicable
UP 10122	SMC 983	Rectal swab	6/11/2018	0						F	A	92.2	112	13.175	Active	Not pregnant	Sclerotized teats
UP 10123	SMC 984	Rectal swab	6/11/2018	0						M	SA	85.9	85	11.519	Not-active	Not scrotal	Not applicable
UP 10124	SMC 985	Rectal swab	6/11/2018	0						F	SA	87.3	97	12.728	Not-active	Not pregnant	Non-sclerotized teats
UP 10125	SMC 986	Rectal swab	6/11/2018	0						F	A	94.6	147	16.426	Active	Pregnant	Not recorded
UP 10126	SMC 987	Rectal swab	6/11/2018	0						F	SA	87.8	86	11.156	Not-active	Not pregnant	Non-sclerotized teats
UP 10127	SMC 988	Rectal swab	6/11/2018	0						F	A	90.6	131	15.959	Active	Pregnant	Sclerotized teats
UP 10128	SMC 989	Rectal swab	6/11/2018	0						M	A	90.3	99	12.141	Active	Not scrotal	Not applicable
UP 10129	SMC 990	Rectal swab	6/11/2018	0						F	A	95.1	142	15.701	Active	Pregnant	Sclerotized teats
UP 10130	SMC 991	Rectal swab	6/11/2018	0						M	A	94.6	141	15.756	Active	Scrotal	Not applicable
UP 10131	SMC 992	Rectal swab	6/11/2018	0						M	A	99.7	139	13.984	Active	Scrotal	Not applicable
UP 10132	SMC 993	Rectal swab	6/11/2018	0						F	SA	87.9	103	13.331	Not-active	Not pregnant	Non-sclerotized teats
UP 10133	SMC 994	Rectal swab	6/11/2018	0						F	A	97	157	16.686	Active	Pregnant	Not recorded
UP 10137	SMC 995	Rectal swab	6/11/2018	0						M	A	96.7	124	13.261	Active	Scrotal	Not applicable
UP 10138	SMC 996	Rectal swab	6/11/2018	0						M	A	97.1	143	15.167	Active	Scrotal	Not applicable
UP 10141	SMC 998	Rectal swab	6/11/2018	0						F	A	91.2	102	12.263	Active	Not pregnant	Not recorded
UP 10142	SMC 999	Rectal swab	6/11/2018	0						M	SA	86.2	95	12.785	Not-active	Not scrotal	Not applicable
UP 10143	SMD 001	Rectal swab	6/11/2018	0						M	A	94.3	128	14.394	Active	Scrotal	Not applicable
UP 10144	SMD 002	Rectal swab	6/11/2018	0						M	A	90.1	135	16.630	Active	Scrotal	Not applicable
UP 10145	SMD 003	Rectal swab	6/11/2018	0						F	A	91.3	97	11.637	Active	Not pregnant	Not recorded
UP 10146	SMD 004	Rectal swab	6/11/2018	0						F	A	94	108	12.223	Active	Not pregnant	Not recorded
UP 10147	SMD 005	Rectal swab	6/11/2018	0						M	A	100.1	151	15.070	Active	Scrotal	Not applicable
UP 10148	SMD 006	Rectal swab	6/11/2018	0						M	A	99.3	149	15.111	Active	Scrotal	Not applicable
UP 10149	SMD 007	Rectal swab	6/11/2018	0						M	A	95	143	15.845	Active	Scrotal	Not applicable
UP 10151	SMD 009	Rectal swab	6/11/2018	0						M	A	94.2	131	14.763	Active	Scrotal	Not applicable
UP 10152	SMD 010	Rectal swab	6/11/2018	0						M	A	90.6	128	15.594	Active	Scrotal	Not applicable
UP 10153	SMD 011	Rectal swab	6/11/2018	0						M	A	92.5	133	15.544	Active	Scrotal	Not applicable
UP 10154	SMD 012	Rectal swab	6/11/2018	0						M	A	96.4	146	15.711	Active	Scrotal	Not applicable
UP 10155	SMD 013	Rectal swab	6/11/2018	0						M	A	98.9	150	15.336	Active	Scrotal	Not applicable

UP 10156	SMD 014	Rectal swab	6/11/2018	0						F	SA	88.3	100	12.826	Not-active	Not pregnant	Non-sclerotized teats
UP 10157	SMD 015	Rectal swab	6/11/2018	0						F	SA	88.6	106	13.503	Not-active	Not pregnant	Non-sclerotized teats
UP 10158	SMD 016	Rectal swab	6/11/2018	0						M	A	91.5	116	13.855	Active	Not scrotal	Not applicable
UP 10159	SMD 017	Rectal swab	6/11/2018	0						M	A	89	109	13.761	Active	Not scrotal	Not applicable
UP 10160	SMD 018	Rectal swab	6/11/2018	0						F	SA	87.4	94	12.306	Not-active	Not pregnant	Non-sclerotized teats
UP 10161	SMD 019	Rectal swab	6/11/2018	0						M	A	91.1	104	12.531	Active	Not scrotal	Not applicable
UP 10162	SMD 020	Rectal swab	6/11/2018	0						F	A	88.6	130	16.561	Active	Pregnant	Non-sclerotized teats
UP 10163	SMD 021	Rectal swab	6/11/2018	0						F	A	93.5	151	17.272	Active	Pregnant	Not recorded
UP 10164	SMD 022	Rectal swab	6/11/2018	0						M	A	94.5	156	17.469	Active	Scrotal	Not applicable
UP 10165	SMD 023	Rectal swab	6/11/2018	0						M	A	92.8	125	14.515	Active	Scrotal	Not applicable
UP 10166	SMD 024	Rectal swab	6/11/2018	0						M	A	89.1	113	14.234	Active	Not scrotal	Not applicable
UP 10167	SMD 025	Rectal swab	6/11/2018	0						M	SA	86.2	87	11.709	Not-active	Not scrotal	Not applicable
UP 10168	SMD 026	Rectal swab	6/11/2018	0						F	A	100.7	173	17.060	Active	Pregnant	Not recorded
UP 10169	SMD 027	Rectal swab	6/11/2018	0						M	A	92.1	122	14.383	Active	Scrotal	Not applicable
UP 10170	SMD 028	Rectal swab	6/11/2018	0						M	SA	88.5	114	14.555	Not-active	Not scrotal	Not applicable
UP 10171	SMD 029	Rectal swab	6/11/2018	0						F	A	91.4	128	15.322	Active	Pregnant	Not recorded
UP 10172	SMD 030	Rectal swab	6/11/2018	0						M	A	90.6	130	15.838	Active	Scrotal	Not applicable
UP 9012	SMC 801	Rectal swab	6/11/2018	0					Negative/ negative	M	A	89.9	130	16.085	Active	Scrotal	Not applicable
UP 10253	SMD 031	Rectal swab	15/1/2019	1	Alpha	1	MZ547464	BtCoV/Rousettus/LP/RSA/UP10253/2019		M	SA	75	59	10.489	Not-active	Not scrotal	Not applicable
UP 10254	SMD 032	Rectal swab	15/1/2019	0						M	SA	77.6	73	12.123	Not-active	Not scrotal	Not applicable
UP 10255	SMD 033	Rectal swab	15/1/2019	0						M	SA	81.6	81	12.165	Not-active	Not scrotal	Not applicable
UP 10256	SMD 034	Rectal swab	15/1/2019	0						M	SA	81.7	64	9.588	Not-active	Not scrotal	Not applicable
UP 10257	SMD 035	Rectal swab	15/1/2019	0						M	SA	79.3	68	10.813	Not-active	Not scrotal	Not applicable
UP 10258	SMD 036	Rectal swab	15/1/2019	0						F	A	93.8	114	12.957	Active	Not pregnant	Not recorded
UP 10259	SMD 037	Rectal swab	15/1/2019	0						F	A	93.2	105	12.088	Active	Not pregnant	Sclerotized teats
UP 10260	SMD 038	Rectal swab	15/1/2019	0						F	SA	81	65	9.907	Not-active	Not pregnant	Non-sclerotized teats
UP 10261	SMD 039	Rectal swab	15/1/2019	0						F	A	90.8	100	12.129	Active	Not pregnant	Not recorded
UP 10262	-	Rectal swab	15/1/2019	0						F	A	92.7	104	12.102	Active	Not pregnant	Not recorded
UP 10263	SMD 040	Rectal swab	15/1/2019	0						F	A	89.3	103	12.916	Active	Not pregnant	Not recorded
UP 10264	SMD 041	Rectal swab	15/1/2019	1	Alpha	1	MZ547465	BtCoV/Rousettus/LP/RSA/UP10264/2019	Positive	M	SA	80.1	56	8.728	Not-active	Not scrotal	Not applicable
UP 10265	SMD 042	Rectal swab	15/1/2019	0						M	SA	83.7	82	11.705	Not-active	Not scrotal	Not applicable
UP 10266	SMD 043	Rectal swab	15/1/2019	0						F	SA	79	62	9.934	Not-active	Not pregnant	Non-sclerotized teats
UP 10267	SMD 044	Rectal swab	15/1/2019	0						F	A	89.8	107	13.269	Active	Not pregnant	Not recorded
UP 10268	SMD 045	Rectal swab	15/1/2019	0					Negative	F	SA	78.8	68	10.951	Not-active	Not pregnant	Non-sclerotized teats
UP 10269	SMD 0451	Rectal swab	15/1/2019	0						F	A	92	112	13.233	Active	Not pregnant	Sclerotized teats
UP 10270	SMD 046	Rectal swab	15/1/2019	0						M	SA	77	65	10.963	Not-active	Not scrotal	Not applicable
UP 10271	SMD 048	Rectal swab	15/1/2019	0					Negative	M	SA	83.4	76	10.927	Not-active	Not scrotal	Not applicable
UP 10272	SMD 047	Rectal swab	15/1/2019	0					Negative	F	SA	80.5	60	9.259	Not-active	Not pregnant	Non-sclerotized teats
UP 10273	SMD 049	Rectal swab	15/1/2019	0						F	A	93.6	114	13.012	Active	Not pregnant	Not recorded
UP 10274	SMD 050	Rectal swab	15/1/2019	0						F	A	93.1	109	12.576	Active	Not pregnant	Not recorded
UP 10275	SMD 051	Rectal swab	15/1/2019	0					Negative	M	SA	80.3	73	11.321	Not-active	Not scrotal	Not applicable
UP 10297	SMD 053	Rectal swab	10/2/2019	0						M	SA	80.5	74	11.419	Not-active	Not scrotal	Not applicable
UP 10300	SMD 056	Rectal swab	10/2/2019	0					Negative	F	SA	81	77	11.736	Not-active	Not pregnant	Non-sclerotized teats
UP 10305	SMD 061	Rectal swab	10/2/2019	0					Negative	F	SA	78.3	79	12.886	Not-active	Not pregnant	Non-sclerotized teats
UP 10306	SMD 062	Rectal swab	10/2/2019	0					Negative	F	SA	80	82	12.813	Not-active	Not pregnant	Non-sclerotized teats
UP 10311	SMD 067	Rectal swab	10/2/2019	0					Negative	F	SA	79.8	79	12.406	Not-active	Not pregnant	Non-sclerotized teats
UP 10318	SMD 077	Rectal swab	10/2/2019	0					Negative	M	SA	79.4	73	11.579	Not-active	Not scrotal	Not applicable
UP 10326	SMD 085	Rectal swab	10/2/2019	0						F	SA	82.2	78	11.544	Not-active	Not pregnant	Non-sclerotized teats
UP 10327	SMD 086	Rectal swab	10/2/2019	0						M	SA	80.7	89	13.666	Not-active	Not scrotal	Not applicable
UP 10328	SMD 087	Rectal swab	10/2/2019	0						F	SA	82.5	86	12.635	Not-active	Not pregnant	Non-sclerotized teats
UP 10329	-	Rectal swab	10/2/2019	1	Beta	2	MZ547574	BtCoV/Rousettus/LP/RSA/UP10329/2019		F	SA	83.1	79	11.440	Not-active	Not pregnant	Non-sclerotized teats
UP 10330	-	Rectal swab	10/2/2019	1	Beta	2	MZ547620	BtCoV/Rousettus/LP/RSA/UP10330/2019		M	SA	84	76	10.771	Not-active	Not scrotal	Not applicable
UP 10331	SMD 089	Rectal swab	10/2/2019	0						M	SA	87	90	11.891	Not-active	Not scrotal	Not applicable
UP 10332	SMD 090	Rectal swab	10/2/2019	0						M	SA	80.4	75	11.602	Not-active	Not scrotal	Not applicable
UP 10333	SMD 091	Rectal swab	10/2/2019	0						F	SA	81.7	82	12.285	Not-active	Not pregnant	Non-sclerotized teats
UP 10334	SMD 092	Rectal swab	10/2/2019	0						M	SA	80.5	84	12.962	Not-active	Not scrotal	Not applicable
UP 10335	SMD 093	Rectal swab	10/2/2019	1	Beta	2	MZ547649	BtCoV/Rousettus/LP/RSA/UP10335/2019		M	SA	82.4	83	12.224	Not-active	Not scrotal	Not applicable
UP 10336	SMD 094	Rectal swab	10/2/2019	0					Negative	M	SA	82	77	11.452	Not-active	Not scrotal	Not applicable
UP 10337	SMD 095	Rectal swab	10/2/2019	0						F	SA	78.2	70	11.447	Not-active	Not pregnant	Non-sclerotized teats
UP 10338	SMD 096	Rectal swab	10/2/2019	0					Negative	F	SA	80.5	78	12.037	Not-active	Not pregnant	Non-sclerotized teats
UP 10339	SMD 097	Rectal swab	10/2/2019	1	Beta	2	MZ547616	BtCoV/Rousettus/LP/RSA/UP10339/2019	Positive	M	SA	77.1	73	12.280	Not-active	Not scrotal	Not applicable
UP 10340	SMD 098	Rectal swab	10/2/2019	0						F	SA	82.7	75	10.966	Not-active	Not pregnant	Non-sclerotized teats
UP 10341	SMD 099	Rectal swab	10/2/2019	1	Beta	2	MZ547617	BtCoV/Rousettus/LP/RSA/UP10341/2019		M	SA	79.7	79	12.437	Not-active	Not scrotal	Not applicable
UP 10342	SMD 100	Rectal swab	10/2/2019	0					Negative	M	SA	85.4	84	11.518	Not-active	Not scrotal	Not applicable
UP 10343	SMD 101	Rectal swab	10/2/2019	0						M	SA	82.1	83	12.314	Not-active	Not scrotal	Not applicable
UP 10345	SMD 103	Rectal swab	10/2/2019	1	Beta	2	MZ547618	BtCoV/Rousettus/LP/RSA/UP10345/2019		F	SA	82.5	80	11.754	Not-active	Not pregnant	Non-sclerotized teats
UP 10346	SMD 104	Rectal swab	10/2/2019	0					Negative	M	SA	83	81	11.758	Not-active	Not scrotal	Not applicable
UP 10355	SMD 113	Rectal swab	10/2/2019	1	Beta	2	MZ547619	BtCoV/Rousettus/LP/RSA/UP10355/2019	Positive	M	SA	85.7	83	11.301	Not-active	Not scrotal	Not applicable
UP 10364	SMD 122	Rectal swab	10/2/2019	0					Negative	F	SA	79.3	76	12.086	Not-active	Not pregnant	Non-sclerotized teats

UP 10367	SMD 125	Rectal swab	10/2/2019	0					Negative	F	SA	81.7	74	11.086	Not-active	Not pregnant	Non-sclerotized teats
UP 10370	SMD 128	Rectal swab	10/2/2019	1	Beta	2	MZ547621	BtCoV/Rousettus/LP/RSA/UP10370/2019	Positive	M	SA	83.6	88	12.591	Not-active	Not scrotal	Not applicable
UP 10374	SMD 132	Rectal swab	10/2/2019	0					Negative	M	SA	83.7	88	12.561	Not-active	Not scrotal	Not applicable
UP 10381	SMD 140	Rectal swab	10/2/2019	0					Negative	M	SA	71.3	54	10.622	Not-active	Not scrotal	Not applicable
UP 10392	SMD 146	Rectal swab	10/2/2019	0					Negative	M	SA	85.9	89	12.062	Not-active	Not scrotal	Not applicable
UP 10399	SMD 153	Rectal swab	10/2/2019	0						F	SA	85	80	11.073	Not-active	Not pregnant	Non-sclerotized teats
UP 10420	SMD 174	Rectal swab	10/2/2019	0					Negative	M	SA	79.2	75	11.957	Not-active	Not scrotal	Not applicable
UP 10424	SMD 178	Rectal swab	10/2/2019	0						M	SA	80.6	78	12.007	Not-active	Not scrotal	Not applicable
UP 10434	SMD 188	Rectal swab	10/2/2019	0					Negative	M	SA	83.5	86	12.335	Not-active	Not scrotal	Not applicable
UP 10440	SMD 194	Rectal swab	10/2/2019	0					Negative	F	SA	86.5	91	12.162	Not-active	Not pregnant	Non-sclerotized teats
UP 10444	SMD 198	Rectal swab	10/2/2019	0					Negative	M	SA	86.4	80	10.717	Not-active	Not scrotal	Not applicable
UP 10451	SMD 205	Rectal swab	10/2/2019	0					Negative	F	SA	83.7	72	10.277	Not-active	Not pregnant	Non-sclerotized teats
UP 10452	SMD 206	Rectal swab	10/2/2019	0					Negative	F	SA	82.5	71	10.432	Not-active	Not pregnant	Non-sclerotized teats
UP 10473	SMD 227	Rectal swab	10/2/2019	0					Negative	F	SA	82.6	76	11.139	Not-active	Not pregnant	Non-sclerotized teats
UP 10488	SMD 242	Rectal swab	10/2/2019	0					Negative	F	SA	83.2	81	11.701	Not-active	Not pregnant	Non-sclerotized teats
UP 10496	SMD 249	Rectal swab	10/2/2019	0					Negative	M	SA	80.9	80	12.223	Not-active	Not scrotal	Not applicable
UP 10497	SMD 250	Rectal swab	10/2/2019	0					Negative	M	SA	80.4	75	11.602	Not-active	Not scrotal	Not applicable
UP 10501	SMD 254	Rectal swab	10/2/2019	1	Beta	2	MZ547645	BtCoV/Rousettus/LP/RSA/UP10501/2019	Positive	F	SA	86.7	82	10.909	Not-active	Not pregnant	Non-sclerotized teats
UP 10507	SMD 260	Rectal swab	10/2/2019	0					Negative	F	SA	81.6	81	12.165	Not-active	Not pregnant	Non-sclerotized teats
UP 10528	SMD 281	Rectal swab	10/2/2019	0					Negative	M	SA	82.6	82	12.019	Not-active	Not scrotal	Not applicable
UP 10531	SMD 284	Rectal swab	10/2/2019	0						F	A	90.9	153	18.517	Active	Lactating	Sclerotized teats
UP 10532	SMD 285	Rectal swab	10/2/2019	0						F	A	89.5	150	18.726	Active	Lactating	Sclerotized teats
UP 10535	SMD 288	Rectal swab	10/2/2019	1	Beta	2	MZ547570	BtCoV/Rousettus/LP/RSA/UP10535/2019		F	SA	83.7	87	12.418	Not-active	Not pregnant	Non-sclerotized teats
UP 10537	SMD 290	Rectal swab	10/2/2019	0						F	SA	81	82	12.498	Not-active	Not pregnant	Non-sclerotized teats
UP 10548	SMD 301	Rectal swab	10/2/2019	0					Negative	F	SA	81.1	77	11.707	Not-active	Not pregnant	Non-sclerotized teats
UP 10549	SMD 302	Rectal swab	10/2/2019	1	Beta	2	MZ547622	BtCoV/Rousettus/LP/RSA/UP10549/2019	Positive	F	SA	81.9	89	13.269	Not-active	Not pregnant	Non-sclerotized teats
UP 10553	SMD 306	Rectal swab	10/2/2019	0					Negative	M	SA	77.3	68	11.380	Not-active	Not scrotal	Not applicable
UP 10557	SMD 310	Rectal swab	10/2/2019	0					Negative	M	SA	83.5	81	11.617	Not-active	Not scrotal	Not applicable
UP 10565	SMD 318	Rectal swab	10/2/2019	1	Beta	2	MZ547623	BtCoV/Rousettus/LP/RSA/UP10565/2019	Positive	F	SA	80.5	75	11.574	Not-active	Not pregnant	Non-sclerotized teats
UP 10570	SMD 323	Rectal swab	10/2/2019	0					Negative	F	SA	80.8	84	12.866	Not-active	Not pregnant	Non-sclerotized teats
UP 10593	SMD 346	Rectal swab	10/2/2019	0					Negative	M	SA	83.8	82	11.677	Not-active	Not scrotal	Not applicable
UP 10617	SMD 370	Rectal swab	10/2/2019	0					Negative	M	SA	81.2	79	11.982	Not-active	Not scrotal	Not applicable
UP 10622	SMD 375	Rectal swab	10/2/2019	0					Negative	F	SA	81.4	77	11.621	Not-active	Not pregnant	Non-sclerotized teats
UP 10657	SMD 409	Rectal swab	10/2/2019	0					Negative	F	SA	85.9	80	10.842	Not-active	Not pregnant	Non-sclerotized teats
UP 10670	SMD 422	Rectal swab	10/2/2019	0						M	SA	82.1	89	13.204	Not-active	Not scrotal	Not applicable
UP 10678	SMD 430	Rectal swab	10/2/2019	0					Negative	M	SA	83.8	81	11.534	Not-active	Not scrotal	Not applicable
UP 10687	SMD 439	Rectal swab	10/2/2019	0					Negative	F	SA	77.7	85	14.079	Not-active	Not pregnant	Non-sclerotized teats
UP 10697	SMD 462	Rectal swab	10/2/2019	0					Negative	M	A	92.1	116	13.675	Active	Scrotal	Not applicable
UP 10705	SMD 454	Rectal swab	10/2/2019	0					Negative	F	SA	86.1	85	11.466	Not-active	Not pregnant	Non-sclerotized teats
UP 10709	SMD 450	Rectal swab	10/2/2019	0						M	SA	83.6	79	11.304	Not-active	Not scrotal	Not applicable
UP 10710	SMD 449	Rectal swab	10/2/2019	0						M	SA	84.5	81	11.344	Not-active	Not scrotal	Not applicable
UP 10711	SMD 448	Rectal swab	10/2/2019	0						F	SA	82.5	88	12.929	Not-active	Not pregnant	Non-sclerotized teats
UP 10712	SMD 464	Rectal swab	10/2/2019	1	Beta	2	MZ547624	BtCoV/Rousettus/LP/RSA/UP10712/2019		F	SA	80.1	71	11.066	Not-active	Not pregnant	Non-sclerotized teats
UP 10713	SMD 465	Rectal swab	10/2/2019	0						F	SA	83.3	79	11.385	Not-active	Not pregnant	Non-sclerotized teats
UP 10714	SMD 466	Rectal swab	10/2/2019	0						M	SA	87.6	90	11.728	Not-active	Not scrotal	Not applicable
UP 10715	SMD 467	Rectal swab	10/2/2019	0						M	SA	83.3	82	11.817	Not-active	Not scrotal	Not applicable
UP 10716	SMD 468	Rectal swab	10/2/2019	0						M	SA	83.2	84	12.135	Not-active	Not scrotal	Not applicable
UP 10717	SMD 469	Rectal swab	10/2/2019	0						F	SA	82.2	81	11.988	Not-active	Not pregnant	Non-sclerotized teats
UP 10718	SMD 470	Rectal swab	10/2/2019	0					Negative	F	SA	82.6	78	11.432	Not-active	Not pregnant	Non-sclerotized teats
UP 10719	SMD 471	Rectal swab	10/2/2019	0						M	SA	81.6	77	11.564	Not-active	Not scrotal	Not applicable
UP 10720	SMD 472	Rectal swab	10/2/2019	0						M	SA	85.5	92	12.585	Not-active	Not scrotal	Not applicable
UP 10721	SMD 473	Rectal swab	10/2/2019	1	Beta	2	MZ547646	BtCoV/Rousettus/LP/RSA/UP10721/2019		M	SA	86.4	81	10.851	Not-active	Not scrotal	Not applicable
UP 10722	SMD 474	Rectal swab	10/2/2019	0						F	SA	78.1	71	11.640	Not-active	Not pregnant	Non-sclerotized teats
UP 10723	SMD 475	Rectal swab	10/2/2019	0						M	SA	82.1	79	11.720	Not-active	Not scrotal	Not applicable
UP 10790	SMD 310	Rectal swab	4/3/2019	0					Negative/ negative	M	SA	86	92	12.439	Not-active	Not scrotal	Not applicable
UP 10791	SMD 094	Rectal swab	4/3/2019	0					Negative/ negative	M	SA	84.6	83	11.597	Not-active	Not scrotal	Not applicable
UP 10828	SMD 524	Rectal swab	4/3/2019	0					Negative	F	SA	83	80	11.613	Not-active	Not pregnant	Non-sclerotized teats
UP 10829	SMD 525	Rectal swab	4/3/2019	0					Negative	M	SA	84	82	11.621	Not-active	Not scrotal	Not applicable
UP 10844	SMD 048	Rectal swab	4/3/2019	0					Negative/ negative	M	SA	87.3	90	11.809	Not-active	Not scrotal	Not applicable
UP 10845	SMD 206	Rectal swab	4/3/2019	1	Alpha	1	MZ547466	BtCoV/Rousettus/LP/RSA/UP10845/2019	Negative/ positive	F	SA	84	85	12.046	Not-active	Not pregnant	Non-sclerotized teats
UP 10846	SMD 542	Rectal swab	4/3/2019	0						M	SA	84.5	87	12.184	Not-active	Not scrotal	Not applicable
UP 10847	SMD 543	Rectal swab	4/3/2019	0						F	SA	85.8	92	12.497	Not-active	Not pregnant	Non-sclerotized teats
UP 10848	SMD 544	Rectal swab	4/3/2019	0					Negative	F	SA	86.3	87	11.681	Not-active	Not pregnant	Non-sclerotized teats
UP 10858	SMD 346	Rectal swab	4/3/2019	0					Negative/ negative	M	SA	86.8	89	11.813	Not-active	Not scrotal	Not applicable
UP 10859	SMD 227	Rectal swab	4/3/2019	0					Negative/ negative	F	SA	85.2	81	11.159	Not-active	Not pregnant	Non-sclerotized teats
UP 10860	SMD 125	Rectal swab	4/3/2019	0					Negative/ negative	F	SA	82.9	83	12.077	Not-active	Not pregnant	Non-sclerotized teats
UP 10861	SMC 771	Rectal swab	4/3/2019	1	Alpha	1	MZ547467	BtCoV/Rousettus/LP/RSA/UP10861/2019		M	SA	85.3	82	11.270	Not-active	Not scrotal	Not applicable
UP 10863	SMD 198	Rectal swab	4/3/2019	0					Negative/ negative	M	SA	87.2	84	11.047	Not-active	Not scrotal	Not applicable
UP 10864	SMD 056	Rectal swab	4/3/2019	0					Negative/ negative	F	SA	84	77	10.913	Not-active	Not pregnant	Non-sclerotized teats

UP 10875	SMD 564	Rectal swab	4/3/2019	0					Negative	F	SA	87.8	89	11.545	Not-active	Not pregnant	Non-sclerotized teats
UP 10884	SMD 062	Rectal swab	4/3/2019	1	Alpha	1	MZ547468	BtCoV/Rousettus/LP/RSA/UP10884/2019	Negative/ positive	F	SA	83.6	82	11.809	Not-active	Not pregnant	Non-sclerotized teats
UP 10886	SMD 580	Rectal swab	4/3/2019	0					Negative	F	SA	83.3	82	11.733	Not-active	Not pregnant	Non-sclerotized teats
UP 10897	SM 570	Rectal swab	4/3/2019	0						F	A	95.1	111	12.273	Active	Not pregnant	Sclerotized teats
UP 10898	SMD 566	Rectal swab	4/3/2019	0						F	A	95.3	117	12.882	Active	Not pregnant	Sclerotized teats
UP 10900	SMD 067	Rectal swab	4/3/2019	0					Negative/ negative	F	SA	84	74	10.488	Not-active	Not pregnant	Non-sclerotized teats
UP 10906	SMD 592	Rectal swab	4/3/2019	0					Negative	M	SA	81.5	79	11.894	Not-active	Not scrotal	Not applicable
UP 10911	SMD 597	Rectal swab	4/3/2019	0					Negative	F	SA	88	91	11.751	Not-active	Not pregnant	Non-sclerotized teats
UP 10921	SMD 242	Rectal swab	4/3/2019	1	Beta	2	MZ547577	BtCoV/Rousettus/LP/RSA/UP10921/2019	Negative/ positive	F	SA	84.9	90	12.486	Not-active	Not pregnant	Non-sclerotized teats
UP 10922	SMD 174	Rectal swab	4/3/2019	0					Negative/ negative	M	SA	84.5	80	11.204	Not-active	Not scrotal	Not applicable
UP 10923	SMD 375	Rectal swab	4/3/2019	0					Negative/ negative	F	SA	85.5	82	11.217	Not-active	Not pregnant	Non-sclerotized teats
UP 10924	SMD 609	Rectal swab	4/3/2019	0						F	SA	83	83	12.048	Not-active	Not pregnant	Non-sclerotized teats
UP 10925	SMD 610	Rectal swab	4/3/2019	0						F	SA	83.4	76	10.927	Not-active	Not pregnant	Non-sclerotized teats
UP 10926	-	Rectal swab	4/3/2019	0						M	SA	86.6	82	10.934	Not-active	Not scrotal	Not applicable
UP 10936	SMD 250	Rectal swab	4/3/2019	0					Negative/ negative	M	SA	82.4	76	11.193	Not-active	Not scrotal	Not applicable
UP 10945	SMD 628	Rectal swab	4/3/2019	0						F	SA	83.2	79	11.412	Not-active	Not pregnant	Non-sclerotized teats
UP 10946	SMD 630	Rectal swab	4/3/2019	0						F	SA	82.6	75	10.993	Not-active	Not pregnant	Non-sclerotized teats
UP 10947	SMD 140	Rectal swab	4/3/2019	0					Negative/ negative	M	SA	77.4	70	11.685	Not-active	Not scrotal	Not applicable
UP 10948	SMD 306	Rectal swab	4/3/2019	0					Negative/ negative	M	SA	79.6	84	13.257	Not-active	Not scrotal	Not applicable
UP 10949	SMD 631	Rectal swab	4/3/2019	0						F	SA	82	76	11.303	Not-active	Not pregnant	Non-sclerotized teats
UP 10950	SMD 301	Rectal swab	4/3/2019	0					Negative/ negative	F	SA	85	83	11.488	Not-active	Not pregnant	Non-sclerotized teats
UP 10951	SMD 077	Rectal swab	4/3/2019	0					Negative/ negative	M	SA	82.2	79	11.692	Not-active	Not scrotal	Not applicable
UP 10954	SMD 634	Rectal swab	4/3/2019	0					Negative	F	SA	83.5	79	11.331	Not-active	Not pregnant	Non-sclerotized teats
UP 10955	SMD 635	Rectal swab	4/3/2019	0					Negative	F	SA	79	55	8.813	Not-active	Not pregnant	Non-sclerotized teats
UP 10982	SMD 666	Rectal swab	4/3/2019	0					Negative	M	SA	82.6	78	11.432	Not-active	Not scrotal	Not applicable
UP 11003	SMD 409	Rectal swab	4/3/2019	1	Alpha	1	MZ547469	BtCoV/Rousettus/LP/RSA/UP11003/2019	Negative/ positive	F	SA	88	81	10.460	Not-active	Not pregnant	Non-sclerotized teats
UP 11005	SMD 097	Rectal swab	4/3/2019	0					Positive/ negative	M	SA	82.5	72	10.579	Not-active	Not scrotal	Not applicable
UP 11006	SMD 318	Rectal swab	4/3/2019	0					Positive/ negative	F	SA	82.3	77	11.368	Not-active	Not pregnant	Non-sclerotized teats
UP 11007	SMD 088	Rectal swab	4/3/2019	0						F	SA	77.9	60	9.887	Not-active	Not pregnant	Non-sclerotized teats
UP 11008	SMD 688	Rectal swab	4/3/2019	0						F	SA	80.3	69	10.701	Not-active	Not pregnant	Non-sclerotized teats
UP 11010	SMD 228	Rectal swab	4/3/2019	0						F	SA	71.1	74	14.638	Not-active	Not pregnant	Non-sclerotized teats
UP 11011	SMD 194	Rectal swab	4/3/2019	0					Negative/ negative	F	SA	88.4	92	11.773	Not-active	Not pregnant	Non-sclerotized teats
UP 11012	SMD 424	Rectal swab	4/3/2019	0						M	SA	86.2	89	11.978	Not-active	Not scrotal	Not applicable
UP 11013	SMD 132	Rectal swab	4/3/2019	0					Negative/ negative	M	SA	83	86	12.484	Not-active	Not scrotal	Not applicable
UP 11025	SMB 153	Rectal swab	4/3/2019	0						F	SA	83.1	83	12.019	Not-active	Not pregnant	Non-sclerotized teats
UP 11026	SMD 470	Rectal swab	4/3/2019	0					Negative/ negative	F	SA	84.6	74	10.339	Not-active	Not pregnant	Non-sclerotized teats
UP 11030	SMD 254	Rectal swab	4/3/2019	0					Positive/ negative	F	A	90.4	87	10.646	Active	Not pregnant	Non-sclerotized teats
UP 11031	SMD 712	Rectal swab	4/3/2019	0						F	SA	81	75	11.431	Not-active	Not pregnant	Non-sclerotized teats
UP 11032	SMD 713	Rectal swab	4/3/2019	0						M	SA	85.5	76	10.396	Not-active	Not scrotal	Not applicable
UP 11033	SMD 188	Rectal swab	4/3/2019	0					Negative/ negative	M	SA	84.2	87	12.271	Not-active	Not scrotal	Not applicable
UP 11035	SMD 715	Rectal swab	4/3/2019	0					Negative	M	SA	88.2	95	12.212	Not-active	Not scrotal	Not applicable
UP 11047	SMD 260	Rectal swab	4/3/2019	0					Negative/ negative	F	SA	82.9	80	11.641	Not-active	Not pregnant	Non-sclerotized teats
UP 11048	SMD 205	Rectal swab	4/3/2019	0					Negative/ negative	F	SA	83.7	75	10.706	Not-active	Not pregnant	Non-sclerotized teats
UP 11056	SMD 249	Rectal swab	4/3/2019	0					Negative/ negative	M	SA	83.1	78	11.295	Not-active	Not scrotal	Not applicable
UP 11060	SMD 741	Rectal swab	4/3/2019	0					Negative	M	A	89.7	97	12.056	Active	Not scrotal	Not applicable
UP 11077	SMD 128	Rectal swab	4/3/2019	1	Alpha	1	MZ547470	BtCoV/Rousettus/LP/RSA/UP11077/2019	Positive/ positive different viruses	M	SA	83.1	89	12.888	Not-active	Not scrotal	Not applicable
UP 11078	SMD 370	Rectal swab	4/3/2019	0					Negative/ negative	M	SA	83.4	89	12.796	Not-active	Not scrotal	Not applicable
UP 11089	SMD 302	Rectal swab	4/3/2019	0					Positive / negative	F	SA	84.6	90	12.575	Not-active	Not pregnant	Non-sclerotized teats
UP 11090	SMD 096	Rectal swab	4/3/2019	0					Negative/ negative	F	SA	82.7	84	12.282	Not-active	Not pregnant	Non-sclerotized teats
UP 11091	SMD 281	Rectal swab	4/3/2019	0					Negative/ negative	M	SA	83.9	90	12.786	Not-active	Not scrotal	Not applicable
UP 11097	SMD 323	Rectal swab	4/3/2019	0					Negative/ negative	F	SA	81.4	72	10.866	Not-active	Not pregnant	Non-sclerotized teats
UP 11100	SMD 793	Rectal swab	4/3/2019	0						F	A	89.2	122	15.333	Active	Pregnant	Non-sclerotized teats
UP 11109	SMD 462	Rectal swab	4/3/2019	0					Negative/ negative	M	A	93.6	119	13.583	Active	Scrotal	Not applicable
UP 11126	SMD 819	Rectal swab	4/3/2019	0						M	SA	75.4	58	10.202	Not-active	Not scrotal	Not applicable
UP 11131	SMD 824	Rectal swab	4/3/2019	0					Negative	M	SA	82.6	67	9.820	Not-active	Not scrotal	Not applicable
UP 11133	SMD 061	Rectal swab	4/3/2019	0					Negative/ negative	F	SA	81.6	76	11.414	Not-active	Not pregnant	Non-sclerotized teats
UP 11141	SMD 834	Rectal swab	4/3/2019	1	Beta	2	MZ547625	BtCoV/Rousettus/LP/RSA/UP11141/2019	Positive	M	SA	76	66	11.427	Not-active	Not scrotal	Not applicable
UP 11161	SMD 854	Rectal swab	4/3/2019	0					Negative	F	SA	76.2	69	11.883	Not-active	Not pregnant	Non-sclerotized teats
UP 11177	SMD 870	Rectal swab	4/3/2019	0					Negative	M	SA	77	61	10.288	Not-active	Not scrotal	Not applicable
UP 11187	SMD 877	Rectal swab	2/4/2019	0					Negative	F	SA	86.3	99	13.293	Not-active	Not pregnant	Non-sclerotized teats
UP 11191	SMD 881	Rectal swab	2/4/2019	0						F	A	93.4	118	13.527	Active	Pregnant	Non-sclerotized teats
UP 11192	SMD 882	Rectal swab	2/4/2019	0						F	A	94.6	119	13.297	Active	Pregnant	Sclerotized teats
UP 11193	SMD 883	Rectal swab	2/4/2019	0						M	SA	83.9	85	12.075	Not-active	Not scrotal	Not applicable
UP 11202	SMD 892	Rectal swab	2/4/2019	1	Beta	2	MZ547647	BtCoV/Rousettus/LP/RSA/UP11202/2019	Positive	F	SA	82.9	75	10.913	Not-active	Not pregnant	Non-sclerotized teats
UP 11210	SMD 597	Rectal swab	2/4/2019	1	Beta	2	MZ547626	BtCoV/Rousettus/LP/RSA/UP11210/2019	Negative/ positive	F	SA	87.1	82	10.809	Not-active	Not pregnant	Non-sclerotized teats
UP 11212	SMD 901	Rectal swab	2/4/2019	0						F	SA	87.7	91	11.832	Not-active	Not pregnant	Non-sclerotized teats
UP 11213	SMD 902	Rectal swab	2/4/2019	0						F	SA	80.9	72	11.001	Not-active	Not pregnant	Non-sclerotized teats
UP 11220	SMD 580	Rectal swab	2/4/2019	1	Beta	2	MZ547627	BtCoV/Rousettus/LP/RSA/UP11220/2019	Negative/ positive	F	SA	85	88	12.180	Not-active	Not pregnant	Non-sclerotized teats
UP 11222	SMD 122	Rectal swab	2/4/2019	0					Negative/ negative	F	SA	84.1	90	12.725	Not-active	Not pregnant	Non-sclerotized teats

UP 11224	SMD 634	Rectal swab	2/4/2019	0					Negative/ negative	F	SA	85.8	87	11.818	Not-active	Not pregnant	Non-sclerotized teats
UP 11228	SMD 355	Rectal swab	2/4/2019	0						M	SA	85.5	73	9.986	Not-active	Not scrotal	Not applicable
UP 11236	SMD 051	Rectal swab	2/4/2019	0					Negative/ negative	M	SA	86	91	12.304	Not-active	Not scrotal	Not applicable
UP 11237	SMD 854	Rectal swab	2/4/2019	0					Negative/ negative	F	SA	77.6	69	11.458	Not-active	Not pregnant	Non-sclerotized teats
UP 11238	SMD 454	Rectal swab	2/4/2019	0					Negative/ negative	F	SA	88.2	87	11.184	Not-active	Not pregnant	Non-sclerotized teats
UP 11239	SMD 292	Rectal swab	2/4/2019	0						F	SA	82	68	10.113	Not-active	Not pregnant	Non-sclerotized teats
UP 11244	SMD 933	Rectal swab	2/4/2019	0						M	SA	85.6	92	12.556	Not-active	Not scrotal	Not applicable
UP 11245	SMD 525	Rectal swab	2/4/2019	0					Negative/ negative	M	SA	85.9	83	11.248	Not-active	Not scrotal	Not applicable
UP 11246	SMD 934	Rectal swab	2/4/2019	0						F	SA	83.2	82	11.846	Not-active	Not pregnant	Non-sclerotized teats
UP 11247	SMD 700	Rectal swab	2/4/2019	1	Beta	2	MZ547573	BtCoV/Rousettus/LP/RSA/UP11247/2019		F	SA	85.5	79	10.807	Not-active	Not pregnant	Non-sclerotized teats
UP 11248	SMD 935	Rectal swab	2/4/2019	0						M	SA	83.4	83	11.933	Not-active	Not scrotal	Not applicable
UP 11256	SMD 943	Rectal swab	2/4/2019	0						F	SA	85	75	10.381	Not-active	Not pregnant	Non-sclerotized teats
UP 11259	SMD 242	Rectal swab	2/4/2019	0					Negative/ positive/ negative	F	SA	85.1	87	12.013	Not-active	Not pregnant	Non-sclerotized teats
UP 11260	SMD 945	Rectal swab	2/4/2019	0						F	A	92.2	113	13.293	Active	Not pregnant	Sclerotized teats
UP 11261	SMD 188	Rectal swab	2/4/2019	0					Negative/ negative /negative	M	SA	84.5	90	12.605	Not-active	Not scrotal	Not applicable
UP 11266	SMD 951	Rectal swab	2/4/2019	0						M	SA	87.2	97	12.757	Not-active	Not scrotal	Not applicable
UP 11267	SMD 952	Rectal swab	2/4/2019	0						F	SA	81.1	78	11.859	Not-active	Not pregnant	Non-sclerotized teats
UP 11268	SMD 953	Rectal swab	2/4/2019	0					Negative	F	SA	83.4	91	13.083	Not-active	Not pregnant	Non-sclerotized teats
UP 11269	SMD 954	Rectal swab	2/4/2019	0						M	SA	84.4	94	13.196	Not-active	Not scrotal	Not applicable
UP 11271	SMD 956	Rectal swab	2/4/2019	0					Negative	F	SA	81.5	76	11.442	Not-active	Not pregnant	Non-sclerotized teats
UP 11274	SMD 870	Rectal swab	2/4/2019	0					Negative/ negative	M	SA	80.2	75	11.660	Not-active	Not scrotal	Not applicable
UP 11287	SMD 100	Rectal swab	2/4/2019	0					Negative/ negative	M	SA	88.7	95	12.075	Not-active	Not scrotal	Not applicable
UP 11288	SMC 972	Rectal swab	2/4/2019	0					Negative/ negative	F	A	92.9	105	12.166	Active	Not pregnant	Sclerotized teats
UP 11290	SMD 972	Rectal swab	2/4/2019	0					Negative	F	A	89.1	89	11.211	Active	Not pregnant	Non-sclerotized teats
UP 11291	SMD 973	Rectal swab	2/4/2019	0						F	SA	85.1	79	10.909	Not-active	Not pregnant	Non-sclerotized teats
UP 11292	SMD 974	Rectal swab	2/4/2019	0						M	SA	85.1	81	11.185	Not-active	Not scrotal	Not applicable
UP 11293	SMD 975	Rectal swab	2/4/2019	1	Beta	**2**	MZ547628	BtCoV/Rousettus/LP/RSA/UP11293/2019		F	SA	83.4	76	10.927	Not-active	Not pregnant	Non-sclerotized teats
UP 11294	SMD 976	Rectal swab	2/4/2019	0						M	A	89.6	93	11.584	Active	Not scrotal	Not applicable
UP 11295	SMD 977	Rectal swab	2/4/2019	1	Beta	**2**	MZ547629	BtCoV/Rousettus/LP/RSA/UP11295/2019		F	SA	82.7	78	11.405	Not-active	Not pregnant	Non-sclerotized teats
UP 11296	SMD 635	Rectal swab	2/4/2019	0					Negative/ negative	F	SA	81.4	68	10.263	Not-active	Not pregnant	Non-sclerotized teats
UP 11307	SMD 988	Rectal swab	2/4/2019	0						F	SA	83	77	11.177	Not-active	Not pregnant	Non-sclerotized teats
UP 11308	SMD 989	Rectal swab	2/4/2019	0						M	A	93.8	114	12.957	Active	Scrotal	Not applicable
UP 11309	SMD 990	Rectal swab	2/4/2019	0						M	SA	85.6	83	11.327	Not-active	Not scrotal	Not applicable
UP 11310	SMD 991	Rectal swab	2/4/2019	0						M	A	92	106	12.524	Active	Not scrotal	Not applicable
UP 11311	SMD 430	Rectal swab	2/4/2019	1	Beta	2	MZ547630	BtCoV/Rousettus/LP/RSA/UP11311/2019	Negative/ positive	M	SA	86.5	83	11.093	Not-active	Not scrotal	Not applicable
UP 11318	SMD 097	Rectal swab	2/4/2019	0					Positive/ negative/ negative	M	SA	80.5	83	12.808	Not-active	Not scrotal	Not applicable
UP 11326	SMD 592	Rectal swab	2/4/2019	1	Beta	2	MZ547631	BtCoV/Rousettus/LP/RSA/UP11326/2019	Negative/ negative	M	SA	82.7	73	10.674	Not-active	Not scrotal	Not applicable
UP 11327	SMD 834	Rectal swab	2/4/2019	0					Positive / negative	M	SA	79.8	74	11.621	Not-active	Not scrotal	Not applicable
UP 11328	SMD 564	Rectal swab	2/4/2019	0					Negative/ negative	F	SA	87.8	91	11.805	Not-active	Not pregnant	Non-sclerotized teats
UP 11360	SME 015	Rectal swab	14/5/2019	0						M	SA	84.6	82	11.457	Not-active	Not scrotal	Not applicable
UP 11369	SMD 146	Rectal swab	14/5/2019	0					Negative/ negative	M	A	90	95	11.728	Active	Not scrotal	Not applicable
UP 11370	SMD 113	Rectal swab	14/5/2019	0					Positive/ negative	M	A	90.7	99	12.034	Active	Not scrotal	Not applicable
UP 11371	SME 016	Rectal swab	14/5/2019	0						M	SA	87.6	92	11.989	Not-active	Not scrotal	Not applicable
UP 11372	SME 017	Rectal swab	14/5/2019	0						M	A	89.9	101	12.497	Active	Not scrotal	Not applicable
UP 11373	SME 018	Rectal swab	14/5/2019	0						M	SA	84.3	96	13.509	Not-active	Not scrotal	Not applicable
UP 11374	SME 002	Rectal swab	14/5/2019	0						F	SA	81.4	79	11.923	Not-active	Not pregnant	Non-sclerotized teats
UP 11375	SMD 877	Rectal swab	14/5/2019	0					Negative/ negative	F	SA	80.1	75	11.690	Not-active	Not pregnant	Non-sclerotized teats
UP 11376	SME 019	Rectal swab	14/5/2019	0						F	SA	84.2	74	10.438	Not-active	Not pregnant	Non-sclerotized teats
UP 11377	SME 020	Rectal swab	14/5/2019	1	Beta	2	MZ547632	BtCoV/Rousettus/LP/RSA/UP11377/2019		F	A	89.3	93	11.662	Active	Not pregnant	Non-sclerotized teats
UP 11378	SME 021	Rectal swab	14/5/2019	0						F	SA	86.4	80	10.717	Not-active	Not pregnant	Non-sclerotized teats
UP 11379	SME 022	Rectal swab	14/5/2019	0						M	SA	84	71	10.062	Not-active	Not scrotal	Not applicable
UP 11380	SME 023	Rectal swab	14/5/2019	0						M	A	89.3	98	12.289	Active	Not scrotal	Not applicable
UP 11381	SME 024	Rectal swab	14/5/2019	0						M	SA	86.8	91	12.078	Not-active	Not scrotal	Not applicable
UP 11382	SMD 666	Rectal swab	14/5/2019	1	Beta	2	MZ547633	BtCoV/Rousettus/LP/RSA/UP11382/2019	Negative/ positive	M	SA	86.6	82	10.934	Not-active	Not scrotal	Not applicable
UP 11383	SME 025	Rectal swab	14/5/2019	0						M	A	96.9	149	15.869	Active	Scrotal	Not applicable
UP 11384	SME 026	Rectal swab	14/5/2019	0						F	SA	88.4	86	11.005	Not-active	Not pregnant	Non-sclerotized teats
UP 11385	SME 027	Rectal swab	14/5/2019	1	Beta	2	MZ547634	BtCoV/Rousettus/LP/RSA/UP11385/2019		F	A	94.1	114	12.874	Active	Not pregnant	Non-sclerotized teats
UP 11386	SME 028	Rectal swab	14/5/2019	1	Beta	2	MZ547635	BtCoV/Rousettus/LP/RSA/UP11386/2019		F	SA	81.9	76	11.330	Not-active	Not pregnant	Non-sclerotized teats
UP 11393	SMD 047	Rectal swab	14/5/2019	0					Negative/ negative	F	SA	85.7	90	12.254	Not-active	Not pregnant	Non-sclerotized teats
UP 11394	SME 035	Rectal swab	14/5/2019	0						M	SA	83.2	87	12.568	Not-active	Not scrotal	Not applicable
UP 11401	SME 042	Rectal swab	14/5/2019	1	Beta	2	MZ547636	BtCoV/Rousettus/LP/RSA/UP11401/2019		M	A	97.5	155	16.305	Active	Scrotal	Not applicable
UP 11402	SMD 041	Rectal swab	14/5/2019	0					Positive/ negative	M	SA	85.9	83	11.248	Not-active	Not scrotal	Not applicable
UP 11403	SMD 439	Rectal swab	14/5/2019	0					Negative/ negative	F	SA	81.7	92	13.783	Not-active	Not pregnant	Non-sclerotized teats
UP 11404	SMD 544	Rectal swab	14/5/2019	0					Negative/ negative	F	A	90	93	11.481	Active	Not pregnant	Non-sclerotized teats
UP 11405	SMD 524	Rectal swab	14/5/2019	0					Negative/ negative	F	SA	82.9	77	11.204	Not-active	Not pregnant	Non-sclerotized teats
UP 11406	SMD 824	Rectal swab	14/5/2019	0					Negative/ negative	M	SA	82	84	12.493	Not-active	Not scrotal	Not applicable
UP 11407	SME 043	Rectal swab	14/5/2019	0						F	SA	83.1	89	12.888	Not-active	Not pregnant	Non-sclerotized teats
UP 11408	SME 044	Rectal swab	14/5/2019	0						F	SA	86.1	86	11.601	Not-active	Not pregnant	Non-sclerotized teats
UP 11409	SME 045	Rectal swab	14/5/2019	1	Beta	2	MZ547637	BtCoV/Rousettus/LP/RSA/UP11409/2019		F	SA	88.3	92	11.800	Not-active	Not pregnant	Non-sclerotized teats

UP 11410	SME 046	Rectal swab	14/5/2019	0						F	A	89.4	89	11.136	Active	Not pregnant	Non-sclerotized teats
UP 11412	SME 048	Rectal swab	14/5/2019	1	Beta	2	MZ547638	BtCoV/Rousettus/LP/RSA/UP11412/2019		F	A	99	95	9.693	Active	Not pregnant	Non-sclerotized teats
UP 11413	SME 049	Rectal swab	14/5/2019	1	Beta	2	MZ547639	BtCoV/Rousettus/LP/RSA/UP11413/2019		M	SA	87.1	96	12.654	Not-active	Not scrotal	Not applicable
UP 11414	SME 050	Rectal swab	14/5/2019	0						M	SA	86.1	78	10.522	Not-active	Not scrotal	Not applicable
UP 11415	SME 051	Rectal swab	14/5/2019	1	Beta	2	MZ547640	BtCoV/Rousettus/LP/RSA/UP11415/2019		M	A	89.4	92	11.511	Active	Not scrotal	Not applicable
UP 11417	SME 053	Rectal swab	14/5/2019	1	Beta	2	MZ547641	BtCoV/Rousettus/LP/RSA/UP11417/2019		F	A	92.4	101	11.830	Active	Not pregnant	Sclerotized teats
UP 11418	SMD 104	Rectal swab	14/5/2019	0					Negative/ negative	M	SA	88.3	94	12.056	Not-active	Not scrotal	Not applicable
UP 11419	SME 054	Rectal swab	14/5/2019	0						F	A	96.1	133	14.401	Active	Not pregnant	Sclerotized teats
UP 11420	SME 055	Rectal swab	14/5/2019	0						F	A	91.8	124	14.714	Active	Not pregnant	Sclerotized teats
UP 11421	SM 977	Rectal swab	14/5/2019	0						F	A	97	129	13.710	Active	Not pregnant	Sclerotized teats
UP 11422	SME 056	Rectal swab	14/5/2019	0						F	A	97.5	134	14.096	Active	Not pregnant	Sclerotized teats
UP 11431	SME 065	Rectal swab	14/5/2019	0						F	SA	80.3	108	16.749	Not-active	Not pregnant	Non-sclerotized teats
UP 11432	SMD 741	Rectal swab	14/5/2019	0					Negative/ negative	F	SA	84.6	86	12.016	Not-active	Not pregnant	Non-sclerotized teats
UP 11433	-	Rectal swab	14/5/2019	1	Beta	2	MZ547642	BtCoV/Rousettus/LP/RSA/UP11433/2019		F	SA	88.4	96	12.285	Not-active	Not pregnant	Non-sclerotized teats
UP 11434	SMA 157	Rectal swab	14/5/2019	0						F	A	99.1	119	12.117	Active	Not pregnant	Sclerotized teats
UP 11435	SMD 892	Rectal swab	14/5/2019	0					Positive/ negative	F	SA	84.8	78	10.847	Not-active	Not pregnant	Non-sclerotized teats
UP 11436	SMD 972	Rectal swab	14/5/2019	0					Negative/ negative	F	A	89.2	94	11.814	Active	Not pregnant	Non-sclerotized teats
UP 11448	SME 077	Rectal swab	14/5/2019	0						M	A	96.6	159	17.039	Active	Scrotal	Not applicable
UP 11449	SMD 715	Rectal swab	14/5/2019	1	Beta	2	MZ547643	BtCoV/Rousettus/LP/RSA/UP11449/2019	Negative/ positive	M	SA	88.3	101	12.954	Not-active	Not scrotal	Not applicable
UP 11451	SME 079	Rectal swab	14/5/2019	1	Beta	2	MZ547644	BtCoV/Rousettus/LP/RSA/UP11451/2019		M	SA	83.1	79	11.440	Not-active	Not scrotal	Not applicable
UP 11452	SME 080	Rectal swab	14/5/2019	0						F	SA	86.9	89	11.786	Not-active	Not pregnant	Non-sclerotized teats
UP 11459	SMD 045	Rectal swab	14/5/2019	0					Negative/ negative	F	SA	87.4	96	12.567	Not-active	Not pregnant	Non-sclerotized teats
UP 11460	SME 087	Rectal swab	14/5/2019	0						M	SA	88.8	100	12.682	Not-active	Not scrotal	Not applicable
UP 11461	SMD 635	Rectal swab	14/5/2019	0					Negative/ negative /negative	F	SA	82.1	65	9.643	Not-active	Not pregnant	Non-sclerotized teats
UP 11463	SMD 956	Rectal swab	14/5/2019	0					Negative/ negative	F	SA	84.2	76	10.720	Not-active	Not pregnant	Non-sclerotized teats
UP 11464	SMD 953	Rectal swab	14/5/2019	0					Negative/ negative	F	SA	83.5	85	12.191	Not-active	Not pregnant	Non-sclerotized teats
